# Towards a competency framework for wellbeing economy in public health: a scoping review

**DOI:** 10.3389/phrs.2026.1609947

**Published:** 2026-08-05

**Authors:** Pablo Rodríguez-Feria, Katarzyna Czabanowska, Mariana Dyakova, Natalia Giraldo-Noack, Timo Clemens

**Affiliations:** 1 Department of International Health, Care and Public Health Research Institute (CAPHRI), Faculty of Health, Medicine and Life Sciences (FHML), Maastricht University, Maastricht, Netherlands; 2 World Health Organization (WHO) CC for Public Health Leadership and Workforce Development, Maastricht, Netherlands; 3 Department of Health Policy Management, Institute of Public Health, Faculty of Health Sciences, Jagiellonian University, Krakow, Poland; 4 World Health Organization (WHO) Collaborating Centre on Investment for Health and Wellbeing, Public Health Wales, Cardiff, United Kingdom; 5 Hertie School gGmbH, Berlin, Germany

**Keywords:** competencies, competency framework, public health, scoping review, wellbeing economy

## Abstract

**Objective:**

This scoping review aims to develop a competency framework, based on the four wellbeing economy capitals, to support education, training and up-skilling of public health professionals and practitioners.

**Methods:**

The JBI methodological guidance and the PRISMA Extension for scoping reviews were used. We searched Medline via PubMed, EMBASE via Ovid, Global Health via EBSCOHost, and ERIC via EBSCOHost. Data extraction focused on four capitals and was mapped against four actions of wellbeing economy.

**Results:**

The proposed Competency Framework organizes 31 competencies within five domains including: Ethics and professionalism, Determinants of health and wellbeing, Digital transformation and data literacy, Policy, politics and governance, and Building capacity for sustainable wellbeing economy.

**Conclusion:**

Developing a competency framework for wellbeing economy in public health is an important step towards defining the field, providing the basis for training and performance assessment, as well as public health policy and capacity development. It can serve as an actionable tool showing how public health stakeholders and leaders can best improve the wellbeing of their communities and citizens.

## Introduction

There is a growing global movement towards building “wellbeing economies” that place human, societal and ecological capitals at the center of policy making and sustainable economic development. The concept of ‘wellbeing economy’ (WBE), that is, an economy that prioritizes people and planet over profit, is gaining support amongst governments, policymakers, budget holders, businesses, and civil society [1]. The Organization for Economic Cooperation and Development (OECD) defines the Economy of Wellbeing as “capacity to create a virtuous circle in which citizens wellbeing drives economic prosperity, stability and resilience, and *vice versa* and that these good macroeconomic outcomes can sustain wellbeing investments over time” [2].

According to the World Health Organization (WHO) Regional Office for Europe (WHO/EURO), the European Union (EU), World Bank (WB), OECD, academic institutions, and the Wellbeing Economy Alliance (WEAll), humanity must renew its commitment to safeguarding the planet and the prosperity of people, communities and societies. We are not only facing environmental challenges that impact human and ecological health, but also social challenges such as conflict, rising cost of living, declining social cohesion, and growing distrust in institutions [3–8].

To address these challenges, four cross-cutting policy priorities, known as “wellbeing capitals”, have been put forward by WHO, integrating the concept of a WBE into *human, social, planetary, and economic wellbeing* (Supplementary Material 1). Healthy societies that have universal policies for housing and food, lifelong learning and literacy, and maintain a stable ecological balance, are building economies that ensure the prosperity of people, civil society and the planet focusing on equity, inclusion, and sustainability [3]. Although the four capitals have been recognized by many countries as critical and direction setting, they have not been integrated in the WBE research so far [3, 9–11].

Building healthier prosperous societies for all requires a wide range of actors across all sectors to apply the WBE principles and approaches. In fact, WHO/EURO has identified 12 stakeholder groups that should undertake four key actions to promote, implement, and evaluate the WBE approach [3]. The WB, WHO/EURO, and Mason and Büchs emphasize the need for strong competencies to educate and train key stakeholders, as well as the importance of using new digital technologies to support this goal [9–13]. The concept of wellbeing has been developed in the health sciences and in other sciences, emphasizing the immense importance it holds for human beings. WBE benefits from bringing disciplines together, developing new cross-disciplinary concepts, and integrating empirical studies with theoretical frameworks and models which could provide valuable insights into how societies can achieve high wellbeing [14].

Layard and Neve apply the concept in scientific and policy contexts to examine how government actions relate to wellbeing, which then influences voting, and can guide policy and investment choices [15]. Few forward looking countries have already adopted and implementing WBE principles into economic and social systems, such as Scotland, Wales, New Zealand, Finland, Iceland, Costa Rica and Bhutan [16]. They identified several barriers to transforming the economic system, including a lack of clarity regarding key concepts and their practical implications, as well as the slow emergence of benefits [16]. They emphasize that WBE requires cooperation, mobilization, and education among stakeholders. The critical importance and an urgent need for synergetic, coordinated and joined-up understanding, know-how and work across disciplines, sectors and levels of government towards building WBE’s is highlighted by the final report of the WHO Council on the Economics of Health for All, leading to WHO and its Member States adopting a Resolution on the Economics of Health for All in 2024 [17, 18]. This will be followed by a WHO Global Strategy being developed for adoption in 2026, which will need to be implemented by WHO and countries alike–through key stakeholders’ alignment and collaboration across health, economy and finance. WHO/EURO is progressing this agenda through a WBE Initiative focusing on recognized public health priorities for investment with multiple co-benefits for economic sustainability, resilience and social cohesion [19]. These include ageing well, youth mental health and inclusion, and addressing rural-urban disparities, requiring multiple sectors to work together; as well as generating novel sources of funding to enable practical, sustainable change [20].

The public health workforce (PHW) plays a particularly significant role, as it operates across many of the 12 stakeholder groups identified by WHO/EURO, such as academia, ministries, banks, and funding bodies. Several competency frameworks already exist to guide its education and training, such as the WHO-ASPHER Competency Framework for the Public Health Workforce in the European Region and the UK Faculty of Public Health (UK FPH) Core Competences in public health practice [21, 22].

However, these frameworks primarily focus on public health functions and were developed before the emergence of the WBE and its associated capitals [21, 22]. Moreover, education and skills building is essential for applying WBE principles and its capitals across the PHW [16]. Therefore, the aim of this scoping review is to develop and propose a competency framework based on four WBE capitals to support the education and training of the PHW.

## Methods

We conducted a scoping review identifying competencies related to the new concept of WBE as defined by the WHO/EURO and its Member States [1, 3–10, 23]. Additionally, a scoping review identifies and analyzes knowledge gaps, such as the absence of competencies that align with the four actions in the WBE agenda [1, 3–10, 23].

The JBI methodological guidance for scoping reviews and the PRISMA Extension for Scoping Reviews guideline were used (Supplementary Material 2) [24–27]. JBI proposed the mnemonic PCC (Population, Concept, and Context) and in this review, the population was attributed to public health, reflecting the health of people and the planet; the concept of interest was the WBE and its capitals; and the context comprises competency-based education and training frameworks.

### Protocol and registration

The protocol was registered on the Open Science Framework platform, which was recommended by JBI, and can be accessed at the following link: https://osf.io/5uxpb/?view_only=6b16bd0e570345c8836e2e58fdb7894a. It had one amend from the original protocol.

### Eligibility criteria

PCC, types of sources of evidence, and limits were considered in establishing the eligibility criteria (Supplementary Material 3). For example, inclusion criteria included literature related to public health for both humans and the planet; literature that defined the WBE and identified at least one wellbeing capital; literature that included a methodology; and articles or grey literature that were available in full. Additionally, systematic reviews, rapid reviews, and umbrella reviews registered on platforms in accordance with international ethical standards were included. Finally, literature published in English was considered, with no restrictions on the date of publication.

### Information sources

Four strategies were used to identify literature for this review. First, the databases used included Medline via PubMed, EMBASE via Ovid, Global Health via EBSCOHost, and ERIC via EBSCOHost. The final database search was conducted on January 15, 2025. Second, as the authors reviewed the literature retrieved from the databases, they identified multilateral organizations that had contributed to the WBE. This search was conducted by one person and began on February 1, 2025.

1. WHO–European Wellbeing Economy Initiative: The News, Publications, and Documents sections were reviewed.
2. WHO–Institutional Repository for Information Sharing (IRIS): A search was conducted using the term “wellbeing economy” in the general search field.
3. WEAll: The News and Resources sections were explored.
4. OECD: The phrase “wellbeing economy” was searched using quotation marks. When a country was mentioned, the titles under the Further Information section were reviewed.
5. WB: Within the Research and Publications section, the term “wellbeing” was used for the search.

Third, handsearching was performed on the literature identified through the previous two mechanisms. Backward searching involved reviewing the reference lists of selected studies, while forward searching was conducted by entering the titles of selected publications (in quotation marks) into Google Scholar to find subsequent citations. Fourth, the corresponding authors of each included source were contacted to inquire about any additional literature related to the PCC framework. All four mechanisms adhered to the established eligibility criteria.

### Search

JBI recommended a two-step process to optimize the search strategy across the selected databases. In the first phase, two databases were selected, and an initial search was conducted. In the second phase, the search strategy was refined using the identified keywords in titles and abstracts, indexed terms such as MeSH and Emtree, Boolean operators (e.g., *OR*, *AND*), and truncation techniques. Supplementary Material 4 contained the search strategy for each database.

### Selection of sources of evidence

The literature obtained from the first mechanism was entered into Rayyan for de duplication [28]. A pilot test of ten titles and abstracts was conducted by two authors, after which these were independently reviewed by both authors. Subsequently, one author read the full text of each piece of literature and applied the eligibility criteria to either include or exclude it. A second author verified the first author’s decisions.

For the second, third, and fourth type of information sources, one author initially reviewed the titles of the documents. If the author considered the document may contain relevant competencies, the full text was read. A second author verified the inclusion criteria for the selected literature. Conflicts were resolved by the two authors, and in case of disagreement, a third author was consulted. The reasons for excluding literature after full review were documented.

### Data charting process and data items

A pilot test was conducted using five selected units of analysis, and items were extracted by one author and reviewed by another author. We extracted the next data: (1) reference, (2) contact author, (3) aim, (4) focus: WBE or its capitals, (5) framework: title, (6) framework: structure, (7) interpretation & conceptualization: frameworks and domains, (8) tables: Frameworks (domains, subdomains and/or competencies), (9) financial support and (10) conflict of interest (Supplementary Materials 5, 6).

### Synthesis of results

The steps for synthesizing the results were based on the cross-walking process methodology, which has been used to study competencies in public health professional education [29]. First, one author extracted each competency and domain separately into a Word document, and this extraction was confirmed by a second author. Second, one author analyzed the verbs and the complete descriptions of the competencies to eliminate duplicates, identify emerging competencies, and rewrite them, and this process was reviewed by three authors.

Third, each competency was mapped against one or all four actions of the WBE. The mapping was done by two co-authors, and two tables were created. The first table includes the competencies linked to at least three actions, while the second table includes competencies linked to one or two actions. This process helped identify actions which were not supported by the competencies based on the literature. If the authors determined a knowledge gap, they reviewed the competencies again from the beginning of the data extraction process to address it. If it was not possible to fill the gap based on the review, new competencies were proposed. Fourth, the list of competencies was rewritten and refined. The domains were proposed to organize. The authors reviewed the draft framework.

## Results

**FIGURE 1:**
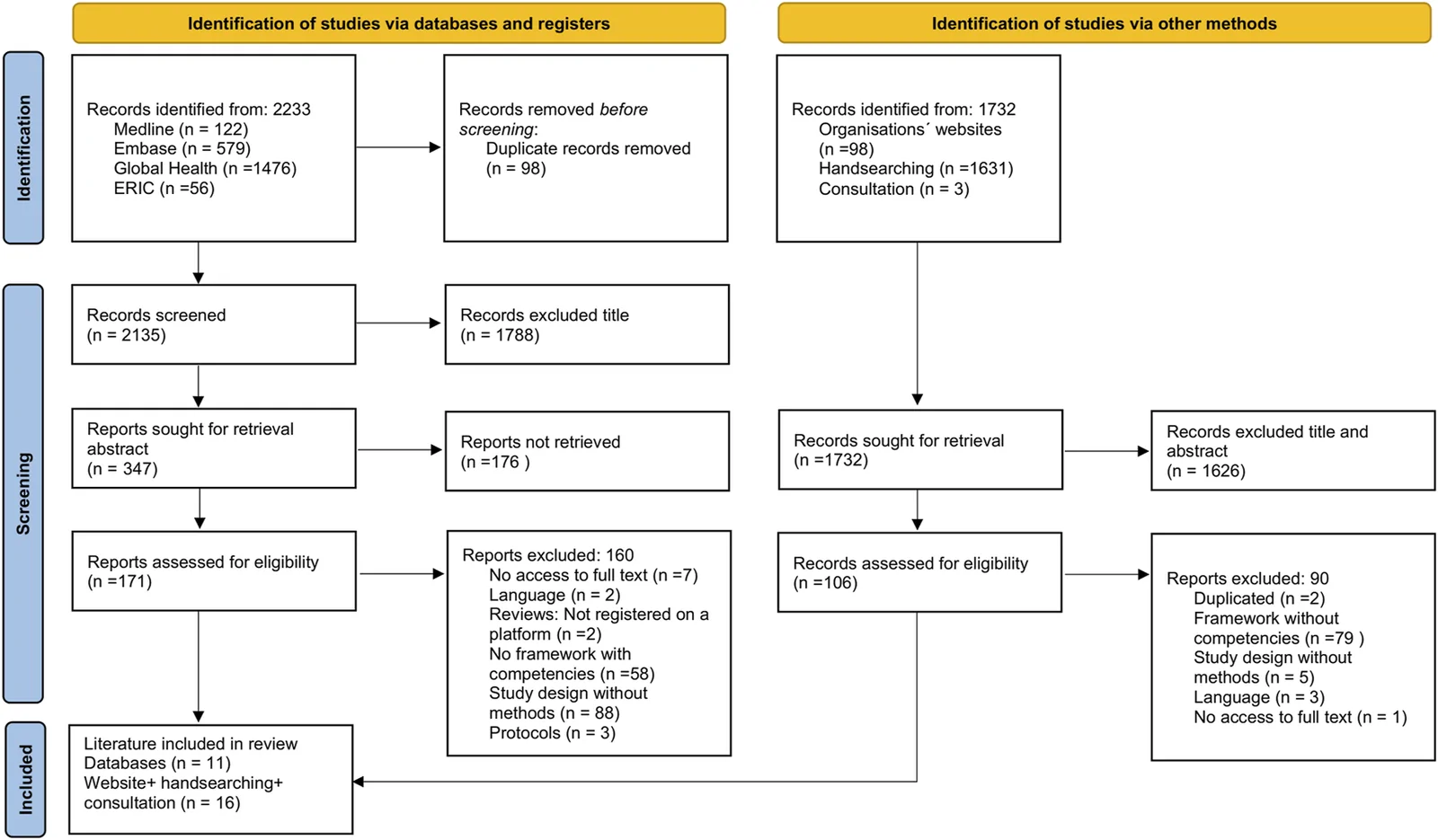
PRISMA 2020 flow diagram for new systematic reviews which included searches of databases, registers and other sources (scoping review, global perspective, 2013–2025).

A total of 3,965 titles were identified through the four mechanisms (Figure 1). Twenty-seven documents were identified, representing 23 units of analysis that included the WBE and/or one of the four capitals, 38 frameworks, 54 domains, and 423 competencies (Table 1 and Supplementary Materials 5, 6). Supplementary Material 7 presents the reasons to include or exclude literature after full-text reading.

**TABLE 1:** Characterisation of literature that has been included in this scoping review (scoping review, global perspective, 2013–2025).

References	Unit of analysis	Reference	Mechanism used to collect literature	Focus: Wellbeing economy and at least one capital
1	1	Organisation for economic cooperation and Development. (2025). Working Party on artificial intelligence governance assessing potential future artificial intelligence risks, benefits and policy imperatives perspectives from the OECD expert group on AI futures	Website: Multilateral organization	Human wellbeing Social wellbeing Economic wellbeing Planetary wellbeing
2	2	Armijo, E. and Asnake, T. (2025). The effects of early childhood development (ECD) programs on the socio-emotional wellbeing of children and caregivers in Refugee and Forced Displacement Settings. Education working Paper; no. 6, November 2024. © Washington, DC: World bank. http://hdl.handle.net/10986/42756 License: CC BY-NC 3.0 IGO	Website: Multilateral organization	Human wellbeing Social wellbeing
3	3	Lee, J., & Ž. Žarnic. (2024). The impact of digital technologies on wellbeing: Main insights from the literature *OECD Papers on Wellbeing and Inequalities*, No. 29, OECD Publishing, Paris, https://doi.org/10.1787/cb173652-en	Website: Multilateral organization	Human wellbeing Social wellbeing Economic wellbeing Planetary wellbeing
4	4	Sangha, KK., Dinku, Y., costanza, R., & Poelina, A. (2024). A comprehensive analysis of wellbeing frameworks applied in Australia and their suitability for Indigenous peoples. *International journal of qualitative studies on health and wellbeing*, 19 (1), 2321646. https://doi.org/10.1080/17482631.2024.2321646	Database	Human wellbeing Social wellbeing Economic wellbeing Planetary wellbeing
5	5	Burtscher, M., S. Piano., & B. Welby. (2024). Developing skills for digital government: A review of good practices across OECD governments, *OECD Social, Employment and Migration Working Papers*, No. 303, OECD Publishing, Paris, https://doi.org/10.1787/f4dab2e9-en	Website: Multilateral organization	Human wellbeing Social wellbeing Economic wellbeing Planetary wellbeing
6		Vuorikari, R., Kluzer S., &Punie, Y. (2022). DigComp 2.2: The digital competence framework for citizens - with new examples of knowledge, skills and attitudes, EUR 31006 EN, publications Office of the european Union, Luxembourg, ISBN 978-92-76-48882-8, doi: 10.2760/115376, JRC128415	Handsearching	
7	6	Del Rio, M., Lasley, P., Tallon, L., Kauth, J.-M., Bare, G., McCurdy, L.E., & etzel, R.A. (2024). Performance indicators corresponding to the critical competencies in children’s environmental health. Journal of environmental health. *Journal of Environmental Health*,86 (10):24-29	Database	Human wellbeing Planetary wellbeing
8		Del Rio, M., Lasley, P., Tallon, L., Kauth, J.-M., Bare, G., McCurdy, L.E., & etzel, R.A. (2023). Critical competencies in children’s environmental health. *Journal of Environmental Health*, 85 (6), 26–29	Handsearching	
9	7	Doshmangir, L., Alipouri Sakha, M., Mostafavi, H., Kabiri, N., Ghaffarifar, S., & Takian, A. (2024). Essential core competencies for health policy graduates: a multi-method consensus type study. *Health research policy and systems*, 22 (1), 136. https://doi.org/10.1186/s12961-024-01221-8	Database	Human wellbeing
10	8	Powell, N., Dalton, H., Lawrence-Bourne, J., & Perkins, D. (2024). Co-creating community wellbeing initiatives: What is the evidence and how do they work? *International Journal of Mental Health Systems*, 18 (1). https://doi.org/10.1186/s13033-024-00645-7	Database	Human wellbeing Social wellbeing Economic wellbeing Planetary wellbeing
11	9	Sebong, P. H., Pardosi, J., Goldman, R. E., Suryo, A. P., Susianto, I. A., & Meliala, A. (2024). Identifying Physician public health competencies to address Healthcare needs in Underserved, border, and outer Island areas of Indonesia: A rapid Assessment. Teaching and learning in medicine, 1–12. Advance online publication. https://doi.org/10.1080/10401334.2024.2353573	Database	Human wellbeing Social wellbeing Economic wellbeing
12	10	Scottish government. (2024). Wellbeing economy toolkit: supporting place based economic strategy and policy development. https://www.gov.scot/binaries/content/documents/govscot/publications/advice-and-guidance/2022/11/wellbeing-economy-toolkit-supporting-place-based-economic-strategy-policy-development/documents/wellbeing-economy-toolkit-supporting-place-based-economic-strategy-policy-development/wellbeing-economy-toolkit-supporting-place-based-economic-strategy-policy-development/govscot%3Adocument/wellbeing-economy-toolkit-supporting-place-based-economic-strategy-policy-development.pdf	Handsearching	Wellbeing economy as a unit
13		Scottish government. n.d. Scotland’s national performance framework our purpose, Values and national outcomes https://nationalperformance.gov.scot/	Handsearching	
14		Scottish government. n.d. Local Wellbeing economy Monitor: Guidance. h ttps://view.officeapps.live.com/op/view.aspx?src=https%3A%2F%2Fwww.gov.scot%2Fbinaries%2Fcontent%2Fdocuments%2Fgovscot%2Fpublications%2Fadvice-and-guidance%2F2022%2F11%2Fwellbeing-economy-toolkit-supporting-place-based-economic-strategy-policy-development%2Fdocuments%2Fwellbeing-economy-monitor-guidance-updated-february-2024%2Fwellbeing-economy-monitor-guidance-updated-february-2024%2Fgovscot%253Adocument%2Fwellbeing-economy-monitor-guidance-updated-february-2024.docx&wdOrigin=BROWSELINK	Handsearching	
15	11	Simon, J., Parisi, S., Wabnitz, K., Simmenroth, A., & Schwienhorst-Stich, E.-M. (2023). Ten characteristics of high-quality planetary health education—results from a qualitative study with educators, students as educators and study deans at medical schools in Germany. Frontiers in public health, 11. https://doi.org/10.3389/fpubh.2023.1143751	Handsearching	Human wellbeing Social wellbeing Economic wellbeing Planetary wellbeing
16	12	World health organization european region. (2023). Country deep dive on the wellbeing economy: Iceland. Copenhagen: WHO regional Office for europe. Licence: CC BY-NC-SA 3.0 IGO WHO-EURO-2023-7415-47181-69111-eng.pdf	Website: Multilateral organization	Wellbeing economy as a unit
17	13	Organisation for economic cooperation and development. (2023), *OECD Skills Outlook 2023: Skills for a Resilient Green and Digital Transition*, OECD Publishing, Paris. https://doi.org/10.1787/27452f29-en	Handsearching	Human wellbeing Social wellbeing Economic wellbeing Planetary wellbeing
18	14	Nykiforuk, C. I. J., Belon, A. P., de Leeuw, E., Harris, P., Allen-Scott, L., Atkey, K., Glenn, N. M., Hyshka, E., Jaques, K., Kongats, K., Montesanti, S., Nieuwendyk, L. M., Pabayo, R., Springett, J., & Yashadhana, A. (2023). An action-oriented public health framework to reduce financial strain and promote financial wellbeing in high-income countries. International Journal for Equity in Health, 22 (1). https://doi.org/10.1186/s12939-023-01877-8	Database	Human wellbeing Social wellbeing Economic wellbeing
19	15	Porcelli, A., D’Onise, K., & Pontifex, K. (2023). Public health partner authorities-how a health in all policies approach could support the development of a wellbeing economy. Health Promotion Journal of Australia: Official Journal of Australian Association of Health Promotion Professionals, 34 (3), 671–674. https://doi.org/10.1002/hpja.738	Database	Wellbeing economy as a whole
20	16	Betley, E. C., Sigouin, A., Pascua, P., Cheng, S. H., MacDonald, K. I., Arengo, F., Aumeeruddy-Thomas, Y., Caillon, S., Isaac, M. E., Jupiter, S. D., Mawyer, A., Mejia, M., Moore, A. C., Renard, D., Sébastien, L., Gazit, N., & Sterling, E. J. (2023). Assessing human wellbeing constructs with environmental and equity aspects: A review of the landscape. People and Nature, 5 (6), 1756–1773. https://doi.org/10.1002/pan3.10293	Database	Human wellbeing Social wellbeing Planetary wellbeing Economic wellbeing
21	17	Hayman, D. T. S., Barraclough, R. K., Muglia, L. J., McGovern, V., Afolabi, M. O., N’Jai, A. U., Ambe, J. R., Atim, C., McClelland, A., Paterson, B., Ijaz, K., Lasley, J., ahsan, Q., Garfield, R., chittenden, K., Phelan, A. L., & Lopez Rivera, A. (2023). Addressing the challenges of implementing evidence-based prioritisation in global health. BMJ Global Health, 8(6). https://doi.org/10.1136/bmjgh-2023-012450	Database	Human wellbeing Social wellbeing Planetary wellbeing Economic wellbeing
22	18	Capetola, T., Noy, S., & Patrick, R. (2022). Planetary health pedagogy: preparing health promoters for 21st‐century environmental challenges. Health Promotion Journal of Australia, 33(Suppl 1), 17–21. https://doi.org/10.1002/hpja.641	Database	Human wellbeing Social wellbeing Planetary wellbeing
23	19	Bergstrom Katy., & Ozler, Berk. (2021). Improving the wellbeing of adolescent Girls in developing Countries. Policy research working Paper; No. 9827. © World bank, Washington,DC. https://hdl.handle.net/10986/36478License CC BY 3.0 IGO	Handsearching	Human wellbeing Social wellbeing Economic wellbeing Planetary wellbeing
24	20	Jordan, M. A. (2021). The role of the health coach in a global Pandemic. Global advances in health and Medicine, 10. https://doi.org/10.1177/21649561211039456	Database	Human wellbeing Social wellbeing Economic wellbeing Planetary wellbeing
25	21	World health organization. Regional Office for Europe. (2020). Health and wellbeing in the voluntary national reviews of the 2030 agenda for sustainable development in the WHO european region 2016–2020. World health organization. Regional Office for Europe. https://iris.who.int/handle/10665/340007. License: CC BY-NC-SA 3.0 IGO.	Website: Multilateral organization	Human wellbeing Social wellbeing Economic wellbeing Planetary wellbeing
26	22	Commonwealth of Australia. (2017). National strategic framework for aboriginal and Torres strait Islander peoples’ mental health and social and emotional Wellbeing (2017–2023). Department of the Prime Minister and cabinet, the australian Government.https://www.niaa.gov.au/sites/default/files/documents/publications/mhsewb-framework_0.pdf	Handsearching	Human wellbeing Social wellbeing
27	23	World health organization. Regional Office for Europe. (2013). Health 2020: a european policy framework supporting action across government and society for health and wellbeing (short version). World health organization. Regional Office for Europe. https://iris.who.int/handle/10665/131300	Website: Multilateral organization	Human wellbeing Social wellbeing Economic wellbeing Planetary wellbeing

**FIGURE 2:**
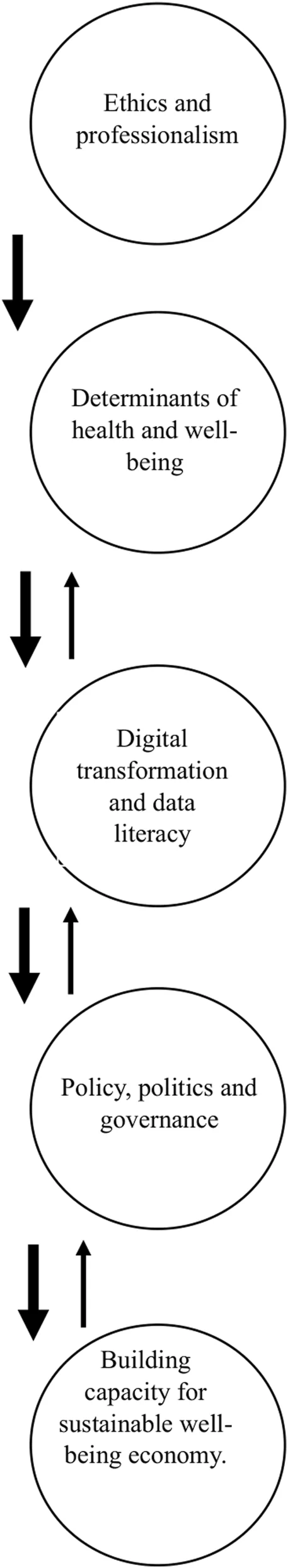
The dynamism of the framework for Wellbeing economy in public health (scoping review, global perspective 2013–2025).

The proposed competency framework for the WBE for Public Health has five domains and 31 competencies, 30 of which are a result of the review, and one was proposed by the researchers (Figure 2; Table 2).

**TABLE 2:** Competency Framework for Wellbeing Economy in Public Health (scoping review, global perspective, 2013–2025).

Domain 1 Ethics and professionalism		Domain 2 Determinants of health and wellbeing		Domain 3 Digital transformation and data literacy		Domain 4 Policy, politics and governance		Domain 5 Building capacity for sustainable wellbeing economy	
Competencies									
1	Is aware of one’s own behavior and values regarding humanity	6	Understands and acts on wider determinants of health including climate and environmental changes	14	Distinguishes and uses diverse data sources to interpret and evaluate health information and digital content	19	Contributes to developing systems to strengthen collaboration, communication, and coordination across sectors and stakeholders for societal wellbeing	25	Acts as a leader, entrepreneur and agent of change to solve problems affecting societal health and wellbeing
2	At all times acts in an open, transparent way according to the rule of law	7	Actively engages in protection of our environment	15	Sorts facts from misinformation, disinformation, and avoids misleading evidence in research	20	Engages with multiple governing sectors from local, national, regional, and international	26	Engages in a lifelong process of learning and growth
3	Understands people’s cultural differences and diversity by examining unconscious bias and judgmental assumptions	8	Actively engages in improving housing security	16	Addresses gaps in digital literacy and integrates them into education and training programs	21	Makes informed judgments about complex wellbeing and health policy issues	27	Uses system thinking and critical thinking to understand and address key drivers of wellbeing outcomes and their interrelations
4	Challenges power differentials in respect for citizens, communities and humanity	9	Actively engages in strengthening access to healthy foods and reducing food insecurity	17	Engages and empowers citizens and communities to participate in the wellbeing economy through digital media	22	Contributes to/or secures sustainable funding to health and wellbeing programs and services	28	Understands and promotes economic evaluation methods to capture wider social benefits of health programs, healthcare interventions, and policies
5	Makes evidence-informed decisions	10	Actively engages in strengthening employment security by addressing workplace fairness	18	Promotes public understanding and awareness of artificial intelligence technologies	23	Engages in health and wellbeing policy development and implementation	29	Actively engages in expanding provision, regulation, and funding of care and social care, education, and transportation services
		11	Promotes high school enrollment, retention, and literacy			24	Actively engages in developing adaptive global health security frameworks, incorporating continuous monitoring, evaluation, and improvement	30	Engages in improving regulation, oversight, and funding of macroeconomic systems and policies for wellbeing goals
		12	Facilitates access to quality primary care					31	Understands and can make use of tools to steer economic investment for wellbeing goals
	​	13	Assess and promotes long-term impacts on the economy on neighborhood-level advantage						

### Ethics and professionalism: domain 1

This domain includes five competencies. *Ethics and professionalism* elaborate on what behaviors a person ought to display. These include self-awareness, transparency, understanding of other people’s cultural backgrounds and life experiences along with power differential challenges, and knowing how to make evidence informed decisions. For example, the competencies state “Is aware of one’s own behavior and values regarding humanity” and ‘At all times acts in an open, transparent way according to the rule of law.

### Determinants of health and wellbeing: domain 2

This domain relates to an understanding of external influences on people’s health related to their environment, economic and social context and how they can be improved to help people. Eight competencies make up this domain, such as: “Understands and acts on wider determinants of health including climate and environmental changes,” “Actively engages in strengthening employment security by addressing workplace fairness,” and “Assesses and promotes the long-term impacts of the economy on neighborhood-level advantage.”

### Digital transformation and data literacy: domain 3

*Digital transformation and data literacy* emphasizes the use of technology to interpret health information, prevent mis and disinformation and engage with others effectively to foster health and wellbeing. This domain has five competencies, including: “Addresses gaps in digital literacy and integrates them into education and training programs,” “Engages and empowers citizens and communities to participate in the wellbeing economy through digital media,” and “Promotes public understanding and awareness of artificial intelligence technologies.”

### Policy, politics and governance: domain 4

*Domain 4* relates to working with others in systems that strengthen collaboration, communication and coordination across sectors and actors and contribute to advancing global health and wellbeing policy. The domain has six competencies, such as: “Makes informed judgments about complex wellbeing and health policy issues,” and “Actively engages in developing adaptive global health security frameworks, incorporating continuous monitoring, evaluation, and improvement.”

### Building capacity for sustainable WBE: domain 5

It involves the life-long development of competencies, and the work needed to be done through education and post-graduate training. It includes seven competencies, such as: “Acts as a leader, entrepreneur, and agent of change to solve problems affecting societal health and wellbeing,” “Uses systems thinking and critical thinking to understand and address key drivers of wellbeing outcomes and their interrelations,” “Actively engages in expanding provision, regulation, and funding of care and social care, education, and transportation services,” and “Understands and can make use of tools to steer economic investment toward wellbeing goals.”

The proposed framework offers a comprehensive and integrated model with a dynamic purpose, drawing on frameworks developed by individuals affiliated with the WHO, OECD, WB, universities, national governments, and the European Commission.

## Discussion

### Main finding of this study

The Competency Framework for the WBE in Public Health is aligned with the four capitals, a perspective not incorporated into existing frameworks, such as the UK FPH and WHO–ASPHER [21, 22]. The proposed framework aims to equip the PHW to participate effectively in and influence the global, European and national WBE initiatives and approaches, including implementing WHO commitments, strategies and recommendations that place health and equity at the centre of policies and investments.

It also addresses the education gap, which has been identified in the literature regarding the competencies needed to apply and mobilize WBE, as well as the gap for applying wellbeing to political science [15, 16]. Because the framework is designed for PHW operating within the organizations identified by the WHO, such as Ministries of Health and senior health sector leadership, national and regional governments, economic and finance policymakers, banks, public health institutions, professional associations, research bodies, universities, and the WHO itself—it may also be relevant and adaptable to other scientific disciplines.

### What is already known on this topic & what this study adds

National and local initiatives have been developed to implement WBE principles, approaches and its associated capitals. A prominent example is Wales, which has adopted and implementing advanced legislation, such as the Wellbeing of Future Generations Act 2015 and the Social Partnership and Public Procurement Act 2023. Similarly, the Government of New Zealand introduced the Wellbeing Budget in 2019, which focuses on improving child wellbeing, building a productive nation, and transforming the economy, among other objectives [30–36].

Various competency frameworks can contribute to the education and training of professionals involved in implementing WBE. For example, both the UK FPH Core competences in public health practice and the WHO-ASPHER Competency Framework align with our proposed framework with their intent to develop competencies aimed at achieving optimal wellbeing [21, 22]. The key distinction, however, is that our suggested framework is explicitly centered on the WBE and its associated capitals. WHO-ASPHER Competency Framework does not include domains and subdomains specifically dedicated to the WBE, but it does contain competencies related to wellbeing, such as: *“*Builds, maintains, and effectively uses strategic alliances, coalitions, professional networks, and partnerships to plan, generate evidence, and implement programmes and services that share common goals and priorities to improve the health and wellbeing of populations” and “Advocates for healthy public policies and services that promote and protect the health and wellbeing of individuals and communities” [21]. In contrast, the UK FPH framework includes two of its ten KA that directly relate to wellbeing: “Use of public health intelligence to survey and assess a population’s health and wellbeing” and “Assessing the evidence of effectiveness of interventions, programmes, and services intended to improve the health or wellbeing of individuals or populations” [22].

The first and second actions relate to using investment, spending, and resources to improve the four wellbeing capitals in a fair and equitable manner, recognizing that investing in wellbeing generates co-benefits across sectors. Douglas et al. and Ashton et al. have also emphasized the importance of policy levers, as they are critical to ensuring recognition for using frameworks such as Health Impact Assessment (HIA) and Social Return on Investment (SROI). These tools allow for the evaluation of potential effects on health and social, economic, and environmental wellbeing, as well as the prioritization of health, equity, and sustainability in policies in countries like Wales and New Zealand [30, 31].

Equity is made possible through values, and our framework begins with domain 1 of ethics and professionalism, where the PHW must uphold values such as fairness, transparency, and the prevention of bias and judgmental assumptions to effectively carry out these first two actions. Developing these competencies aligns closely with the Inner Development Goals (IDGs) initiative, which promotes “inner growth” as a “prerequisite for sustainable outer change”, connecting personal with societal wellbeing and driving collective shift towards “regenerative cultures and economies” [37]. Similarly, the UK FPH framework includes KA #9, “Professional personal and ethical development,” which enables the PHW to shape and evaluate their own personal and professional growth by understanding their behaviors and attitudes and their impacts. It also emphasizes the ability to modify behavior and practice within the framework of relevant professional codes [22]. The WHO-ASPHER Competency Framework aligns with this call as well, emphasizing in its subdomain *Professional Development and Reflective Ethical Practice* the importance of values that support the PHW in performing their roles [21].

Next, our framework in domains 2 and 4 emphasizes the importance of the determinants of health and wellbeing, as well as the relationship between policy, politics, and governance. The final domain 5 focuses on building capacity for the WBE, where five competencies (27–31) highlight the significance of investment, spending, and resources for, along with the benefits of, investing in wellbeing. These domains align well with the fundamental pillars of building economics of health for all, including valuing, financing, innovating and strengthening public sector capacity toward achieving health and wellbeing for all [18]. The WHO-ASPHER Competency Framework aligns too as it includes subdomains on governance and resource management, and leadership and systems thinking, which aim to equip the PHW with competencies in managing resources within systems related to the WBE and its capitals, as well as understanding the broader benefits that WBE approaches bring to other systems [21]. However, the UK FPH framework does not include KA with action verbs related to investing, spending, or resource management for wellbeing, nor does it emphasize systems thinking or critical thinking [22].

The third action focuses on delivering public health goods and policies to create conditions for healthy lives for all, including accessible high-quality services and housing. Our second domain closely relates to social determinants of health (SDH), emphasizing housing in competency 8, which states, “actively engages in improving housing security,” and competency 12, which “facilitates access to quality primary care.” Similarly, the fifth domain includes competency 29, which “actively engages in expanding provision, regulation, and funding of care and social care, education, and transportation services.” Unlike our framework, the UK FPH framework does not specifically mention housing; however, housing is a SDH, and this action can be approximated by KA #5, which covers Health Improvement, Determinants of Health, and Health Communication. Likewise, the WHO-ASPHER Competency Framework includes competencies related to the importance of SDH, such as: “Contributes to or leads community-based health needs assessments, ensuring that these assessments consider biological, social, economic, cultural, political, and physical determinants of health and wider determinants such as deprivation.”

Recognizing that the solutions for producing wellbeing lie with different sectors of government and society and therefore engaging a broad range of actors, including the civil society, as part of implementing the solutions through dialogue, the fourth action emphasizes the importance of collaborative work among various actors in government and society to promote wellbeing. The WHO Resolution on the Economics of Health for All urges Member States to “recognize the importance of putting in place multisectoral capacities and mechanisms … calling for shifts, including equipping and enabling engagement between all relevant sectors including the health and financial sectors…” It highlights the need for health leadership, advocacy, negotiation and diplomacy skills to facilitate cross-sector synergies, common goals and priorities for implementing WBE principles, approaches and measures of success [21]. All three frameworks include competencies that foster collaboration across different actors through various actions, notably leadership competencies. Leadership is recognized in all three frameworks as essential for enabling people to work together to create change aimed at improving wellbeing. This is reflected in our fifth domain through competency 25, in the UK FPH framework’s KA #4: *Strategic Leadership and Collaborative Working for Health*, and in the WHO-ASPHER Competency Framework’s subdomain *Leadership and Systems Thinking* [21, 22]. All three frameworks also highlight the importance of communication among stakeholders. Our framework emphasizes communication and collaboration between government and other actors within the domains of *Digital Transformation and Data Literacy* and *Policy, Politics, and Governance*. Similarly, the WHO-ASPHER Competency Framework includes the subdomains *Collaboration and Partnerships* and *Communication, Culture, and Advocacy*, which focus on communication and collaboration across diverse actors [21]. The UK FPH framework also mentions KA related to communication and working with multiple actors, such as KA #4: *Strategic Leadership and Collaborative Working for Health* and KA #5: *Health Improvement, Determinants of Health, and Health Communication*.

### Limitations of this study

This review has certain limitations, as it includes only literature published in English. However, its comprehensiveness is strengthened by the absence of the publication date restriction and the inclusion of both grey and indexed literature. This limitation could be addressed by combining the scoping review with another methodology such as expert panels or a Delphi technique which could enable the identification of individuals and organizations that do not publish in English but possess and they are interested and have expertise in the topic.

The review provides a solid foundation for the development and implementation of competencies that should be performed by the public health and wider professionals involved in the WBE. The review does not allow for prioritizing the most important competencies for each stakeholder therefore, it needs to be complemented by a consensus study to enable a reality check of the proposed competencies, allowing participating actors to prioritize, propose new ones based on their experience, and determine the relationship of these competencies with the domains proposed in our framework. The development of the WBE competency framework can support education and training of the PHW as well as individual and organizational training needs assessment in diverse public health, government and academic organizations [38].

### Conclusion

The development of the competency framework for WBE in public health is an important step towards defining the field, providing the basis for training and performance assessment as well as public health policy, advocacy and diplomacy development. It can serve as an actionable tool showing how public health stakeholders, professionals, practitioners and leaders can best engage and work across sectors, government levels and borders to protect, improve and promote the wellbeing of communities and citizens while building more resilient, sustainable and prosperous economies for all.
