# Supplementary material for: Towards a competency framework for wellbeing economy in public health: a scoping review
Source: Public Health Rev. 2026 Aug 5;47:1609947. doi: 10.3389/phrs.2026.1609947 (PMC13500908; doi:10.3389/phrs.2026.1609947)
Supplement: Supplementary file 1 [file Supplementaryfile1.docx]

**Supplementary material 1.** Operational definitions, interested actions, and four actions

Table 1 Operational definitions.

| # | Concepts | Definitions | References |
| --- | --- | --- | --- |
| 1 | Competency | The abilities of a person to integrate knowledge, skills and attitudes in their performance of tasks for the context. Competencies are durable, trainable and, through the expression of behaviours, measurable. | World Health Organization. Global competency and outcomes framework for the essential public health functions. World Health Organization. 2024. <https://iris.who.int/handle/10665/376577>. License: CC BY-NC-SA 3.0 IGO |
| 2 | Economic well-being | Workforce well-being increases performance, decreases staff turnover, and has positive outcomes for employees, employers and the overall economy | Health in the well-being economy. Background paper: working together to achieve healthy, fairer, prosperous societies across the WHO European Region. Copenhagen: WHO Regional Office for Europe; 2023. Licence: CC BY-NC-SA 3.0 IGO. |
| 3 | Human well-being | People’s health and their subjective well-being are closely linked; both are drivers of economic prosperity. | Health in the well-being economy. Background paper: working together to achieve healthy, fairer, prosperous societies across the WHO European Region. Copenhagen: WHO Regional Office for Europe; 2023. Licence: CC BY-NC-SA 3.0 IGO. |
| 4 | One health | It is an integrated, unifying approach that aims to sustainably balance and optimize the health of humans, animals, plants and ecosystems. It recognizes the health of humans, domestic and wild animals, plants and the wider environment (including ecosystems) are closely linked and interdependent | FAO, UNEP, WHO, and WOAH. 2022. One Health Joint Plan of Action (2022-2026). Working together for the health of humans, animals, plants and the environment. Rome. https://doi.org/10.4060/cc2289en |
| 5 | Planetary well-being | The environment is key to human well-being and economic prosperity | Health in the well-being economy. Background paper: working together to achieve healthy, fairer, prosperous societies across the WHO European Region. Copenhagen: WHO Regional Office for Europe; 2023. Licence: CC BY-NC-SA 3.0 IGO. |
| 6 | Social well-being | Trust, participation and social cohesion make significant contributions to health and well-being, including health equity, as well as being vital to prosperous and resilient societies. | Health in the well-being economy. Background paper: working together to achieve healthy, fairer, prosperous societies across the WHO European Region. Copenhagen: WHO Regional Office for Europe; 2023. Licence: CC BY-NC-SA 3.0 IGO. |
| 7 | Well-being economy as a goal | It is an economy which secures well-being  for all people today and for future generations, and includes the  well-being of the planet as an overarching goal. | Health in the well-being economy. Background paper: working together to achieve healthy, fairer, prosperous societies across the WHO European Region. Copenhagen: WHO Regional Office for Europe; 2023. Licence: CC BY-NC-SA 3.0 IGO. |
| 8 | Well-being economy as an approach | It is a combination of ways of working  that contribute to achieving the above goals (equity, inclusion and  sustainability). | Health in the well-being economy. Background paper: working together to achieve healthy, fairer, prosperous societies across the WHO European Region. Copenhagen: WHO Regional Office for Europe; 2023. Licence: CC BY-NC-SA 3.0 IGO. |

***Table 2* Stakeholders that should advocate for well-being economy.**

| # | Stakeholders provided by the WHO |
| --- | --- |
| 1 | Ministry of Health and health sector senior managers. |
| 2 | National and regional governments. |
| 3 | Economic and finance policymakers and ministers. |
| 4 | Public health institutions and health professional associations |
| 5 | Development institutions and international financial institutions. |
| 6 | Central Banks. |
| 7 | Civil society. |
| 8 | Research funders and donors. |
| 9 | Investors. |
| 10 | Research institutions and universities. We are here |
| 11 | Employers and businesses. |
| 12 | WHO |

***Table 3 Four actions* for well-being economy**

| 1 | Action 1 | Using public and private investment, spending and resources to improve human, social, planetary and economic well-being in a way that is fair and equitable | Health in the well-being economy. Background paper: working together to achieve healthy, fairer, prosperous societies across the WHO European Region. Copenhagen: WHO Regional Office for Europe; 2023. Licence: CC BY-NC-SA 3.0 IGO. |
| --- | --- | --- | --- |
| 2 | Action 2 | Recognizing that investing in well-being delivers co-benefits across sectors, and therefore optimizing co-benefits from any decision or investment while minimizing any negative impacts | Health in the well-being economy. Background paper: working together to achieve healthy, fairer, prosperous societies across the WHO European Region. Copenhagen: WHO Regional Office for Europe; 2023. Licence: CC BY-NC-SA 3.0 IGO. |
| 3 | Action 3 | Delivering public health goods and policies that create the conditions for healthy lives for all and enable people to thrive, such as accessible and high-quality health services, and secure and affordable housing | Health in the well-being economy. Background paper: working together to achieve healthy, fairer, prosperous societies across the WHO European Region. Copenhagen: WHO Regional Office for Europe; 2023. Licence: CC BY-NC-SA 3.0 IGO. |
| 4 | Action 4 | Recognizing that the solutions for producing well-being lie with different sectors of government and society and therefore engaging a broad range of stakeholders, including the population, as part of implementing the solutions through dialogue. | Health in the well-being economy. Background paper: working together to achieve healthy, fairer, prosperous societies across the WHO European Region. Copenhagen: WHO Regional Office for Europe; 2023. Licence: CC BY-NC-SA 3.0 IGO. |
