# Supplementary material for: Towards a competency framework for wellbeing economy in public health: a scoping review
Source: Public Health Rev. 2026 Aug 5;47:1609947. doi: 10.3389/phrs.2026.1609947 (PMC13500908; doi:10.3389/phrs.2026.1609947)
Supplement: Supplementary file 3 [file Supplementaryfile3.docx]

**Supplementary material 3.** Eligibility criteria

| PCC + types of sources of evidence | Inclusion criteria | Exclusion criteria |
| --- | --- | --- |
| P | - Literature related to public health for humans and the planet. - An approach to planetary health includes the concepts of One Health, Planetary Health, and Ecohealth. | - Literature is not related to public health. - Literature is not related to the approaches to the health of people or the planet: One Health, Planetary Health, and Ecohealth. |
| C | - Literature that defines well-being economy and establishes at least one well-being capital. - Literature that defines well-being economy but does not establish any well-being capital - Literature that defines at least one well-being capital. | - Literature that does not provide a definition/interpretation of well-being economy capital or any of its capitals. |
| C | - The frameworks have a methodology. - The frameworks clearly mention their competencies. | - No framework. - The frameworks do not have a methodology. - The frameworks do not mention all competencies in the consulted literature. |
| Types of sources of evidence | - The article or grey literature can be accessed in full (body of the article, tables, figures, and appendices). - Grey or indexed literature that has a methodology to create the framework. - Systematic reviews, rapid reviews, and umbrella reviews that are registered on a platform. | - Literature or grey literature that cannot be accessed in full (body of the article, tables, figures, and appendices). - Literature that does not have a methodology. - Literature reviews: systematic reviews, rapid reviews, and umbrella reviews that are not registered on a platform such as PROSPERO. - Literature reviews that do not establish methodological guidelines or reporting guidelines. For example, JBI guidelines for methodology and PRISMA for scoping reviews as reporting guidelines. |
| Limits | - Literature published in English. | - Literature published in a language other than English. |
