# Supplementary material for: Towards a competency framework for wellbeing economy in public health: a scoping review
Source: Public Health Rev. 2026 Aug 5;47:1609947. doi: 10.3389/phrs.2026.1609947 (PMC13500908; doi:10.3389/phrs.2026.1609947)
Supplement: Supplementary file 4 [file Supplementaryfile4.docx]

**Supplementary material 4.** Search strategy

First step: two databases

**Table 1. Search strategy for Medline via PubMed.**

| PCC criteria | Words |
| --- | --- |
| Population | 1. Public Health [ ti,ab] 2. Public Health[ Mesh] 3. #1 OR #2 |
| Concept | 4. Well* economy [ ti,ab] 5. Economic well* [ ti,ab] 6. #4 OR #5 |
| Context | 7. Competenc*[ ti,ab] 8. Framework*[ ti,ab] 9. Competency-Based Education[ Mesh] 10. #7 OR #8 OR #9 |
| Sum | 11. #3 AND #6 AND #10 |
| Results and date | 35 results, 16 10^th^ 2024. |

**Table 2. Search strategy for EMBASE via Ovid**

| PCC criteria | Words |
| --- | --- |
| Population | 1. Public Health [ ti] 2. Public Health [ ab] 3. Public health [sh] 4. #1 OR #2 OR #3 |
| Concept | 5. Well?being economy [ ti] 6. Well?being economy [ ab] 7. Economic well* [ ti] 8. Economic well* [ab] 9. economic well-being [sh] 10. #5 OR #6 OR #7 OR #8 OR #9 |
| Context | 11. Competenc*[ ti] 12. Competenc*[ ab] 13. Framework*[ ti] 14. Framework*[ ab] 15. professional competence [sh] 16. #11 OR #12 OR #13 OR #14 OR #15 |
| Sum | 17. #4 AND #10 AND #16 |
| Results and date | 9 results, 16 19^th^ 2024. |

**Table 1: Search strategy for Medline via PubMed**

| PCC mnemotechnic | Words |
| --- | --- |
| Population: Well-being economy for people and the planet. | 1. Public Health [ ti,ab] 2. Planetary health[ ti,ab] 3. Ecohealth [ ti,ab] 4. One health[ ti,ab] 5. Public Health [ Mesh] 6. One health[Mesh] 7. # 1 OR # 2 OR # 3 OR # 4 OR # 5 OR # 6 |
| Concept: Competencies to perform well-being economy | 8. Well&being [ ti,ab] 9. Well&being economy [ ti,ab] 10. Well&being capitals [ ti,ab] 11. Economic well&being [ ti,ab] 12. Financial well&being[ ti,ab] 13. Well&offness[ ti,ab] 14. Socio-economic wellbeing [ ti,ab] 15. Human well&being [ ti,ab] 16. Planetary well&being[ ti,ab] 17. Social well&being [ ti,ab] 18. # 8 OR # 9 OR # 10 OR # 11 OR # 12 OR # 13 OR # 14 OR # 15 OR # 16 OR # 17 |
| Context | 19. Education [ ti,ab] 20. Training [ ti,ab] 21. Professional development [ ti,ab] 22. Organi&ational development [ ti,ab] 23. Framework*[ ti,ab] 24. Model*[ ti,ab] 25. Competenc*[ ti,ab] 26. Knowledge [ ti,ab] 27. Atittud* [ ti,ab] 28. Skill* [ ti,ab] 29. value* [ ti,ab] 30. abilit*[ ti,ab] 31. aptitude*[ ti,ab] 32. expertise[ ti,ab] 33. proficiency[ ti,ab] 34. Competency-Based Education[ Mesh] 35. Professional Competence[ Mesh] 36. Education, Professional [ Mesh] 37. # 19 OR # 20 OR # 21 OR # 22 OR # 23 OR # 24 OR # 25 OR # 26 OR # 27 OR # 28 OR # 29 OR # 30 OR # 31 OR # 32 OR # 33 OR # 34 OR # 35 OR # 36 |
| Sum | 38. # 7 AND # 18 AND # 37 |
| String | **(((((((Public Health[Title/Abstract]) OR (Planetary health[Title/Abstract])) OR (Ecohealth[Title/Abstract])) OR (One health[Title/Abstract])) OR (Public Health[MeSH Terms])) OR (One health[MeSH Terms])) AND ((((((((((Well&being[Title/Abstract]) OR (Well&being economy[Title/Abstract])) OR (Well&being capitals[Title/Abstract])) OR (Economic well&being[Title/Abstract])) OR (Financial well&being[Title/Abstract])) OR (Well&offness[Title/Abstract])) OR (Socio-economic wellbeing[Title/Abstract])) OR (Human well&being[Title/Abstract])) OR (Planetary well&being[Title/Abstract])) OR (Social well&being[Title/Abstract]))) AND ((((((((((((((((((Education[Title/Abstract]) OR (Training[Title/Abstract])) OR (Professional development[Title/Abstract])) OR (Organi&ational development[Title/Abstract])) OR (Framework*[Title/Abstract])) OR (Model*[Title/Abstract])) OR (Competenc*[Title/Abstract])) OR (Knowledge[Title/Abstract])) OR (Atittud*[Title/Abstract])) OR (Skill*[Title/Abstract])) OR (value*[Title/Abstract])) OR (abilit*[Title/Abstract])) OR (aptitude*[Title/Abstract])) OR (expertise[Title/Abstract])) OR (proficiency[Title/Abstract])) OR (Competency-Based Education[MeSH Terms])) OR (Professional Competence[MeSH Terms])) OR (Education, Professional[MeSH Terms]))** |
| Results | 122, 15^th^ January 2025 |

**Table 2. Search strategy for EMBASE via Ovid**

| PCC criteria | Words |
| --- | --- |
| Population: Well-being economy for people and the planet. | 1. Public Health [ ti] 2. Public Health [ ab] 3. planetary health[ ti] 4. planetary health[ ab] 5. Ecohealth [ ti] 6. Ecohealth [ ab] 7. One Health[ ti] 8. One Health [ ab] 9. Public health [sh] 10. planetary health[sh] 11. One Health[sh] 12. #1 OR #2 OR #3 OR #4 OR #5 OR #6 OR #7 OR #8 OR #9 OR #10 OR #11 |
| Concept: Competencies to perform well-being economy | 13. Well?being economy [ ti] 14. Well?being economy [ ab] 15. Well?being capitals [ ti] 16. Well?being capitals [ ab] 17. Economic well?being [ ti] 18. Economic well?being [ab] 19. financial well?being[ ti] 20. financial well?being[ab] 21. Well?offness[ti] 22. Well?offness[ab] 23. Socio-economic well?being [ ti] 24. Socio-economic well?being [ ab] 25. Human well?being [ ti] 26. Human well?being [ab] 27. Planetary well?being[ti] 28. Planetary well?being[ab] 29. Social well?being [ti] 30. Social well?being [ab] 31. wellbeing[sh] 32. #13 OR #14 OR #15 OR #16 OR #17 OR #18 OR #19 OR #20 OR #21 OR #22 OR #23 OR #24 OR #25 OR #26 OR #27 OR #28 OR #29 OR #30 OR #31 |
| Context | 33. Competency[ ti] 34. Competency[ ab] 35. Competencies[ ti] 36. Competencies[ ab] 37. Framework?[ ti] 38. Framework?[ ab] 39. professional competence [sh] 40. conceptual framework[sh] 41. #33 OR #34 OR #35 OR #36 OR #37 OR #38 OR #39 OR #40 OR #41 |
| Sum | 42. #12 AND #32 AND #41 |
| String | **#** \| **Query** \| **Results from 15 Jan 2025**; 1 \| Public Health.ti. or Public Health.ab. or planetary health.ti. or planetary health.ab. or Ecohealth.ti. or Ecohealth.ab. or One Health.ti. or One Health.ab. or Public health.sh. or planetary health.sh. or One Health.sh. \| 564,334; 2 \| Well?being economy.ti. or Well?being economy.ab. or Well?being capitals.ti. or Well?being capitals.ab. or Economic well?being.ti. or Economic well?being.ab. or financial well?being.ti. or financial well?being.ab. or Well?offness.ti. or Well?offness.ab. or Socio-economic well?being.ti. or Socio-economic well?being.ab. or Human well?being.ti. or Human well?being.ab. or Planetary well?being.ti. or Planetary well?being.ab. or Social well?being.ti. or Social well?being.ab. or wellbeing.sh. \| 93,276; 3 \| Competency.ti. or Competency.ab. or Competencies.ti. or Competencies.ab. or Framework?.ti. or Framework?.ab. or professional competence.sh. or conceptual framework.sh. \| 611,386; 4 \| 1 and 2 and 3 \| 579 |
| Results and date | 579 results, 15^th^ January 2025. |

**Table 3. Global health via EBSCOHost**

| PCC criteria | Words |
| --- | --- |
| Population: Well-being economy for people and the planet. | 1. Public Health [ ti] 2. Public Health [ ab] 3. planetary health[ ti] 4. planetary health[ ab] 5. Ecohealth [ ti] 6. Ecohealth [ ab] 7. One Health[ ti] 8. One Health [ ab] 9. Public health [su] 10. #1 OR #2 OR #3 OR #4 OR #5 OR #6 OR #7 OR #8 OR #9 |
| Concept: Competencies to perform well-being economy | 11. Well* economy [ ti] 12. Well* economy [ ab] 13. Well* capitals [ ti] 14. Well* capitals [ ab] 15. Economic well* [ ti] 16. Economic well* [ab] 17. Human well* [ ti] 18. Human well* [ab] 19. Planetary well* [ti] 20. Planetary well* [ab] 21. Social well* [ti] 22. Social well* [ab] 23. #11 OR #12 OR #13 OR #14 OR #15 OR #16 OR #17 OR #18 OR #19 OR #20 OR #21 OR #22 |
| Context | 24. Competenc*[ ti] 25. Competenc*[ ab] 26. Framework*[ ti] 27. Framework*[ ab] 28. Knowledge [ti] 29. Knowledge [ab] 30. Atittud* [ ti] 31. Atittud* [ab] 32. Skill* [ ti] 33. Skill* [ab] 34. value* [ ti] 35. value* [ ab] 36. competency based education[su] 37. professional competence [su] 38. #24 OR #25 OR #26 OR #27 OR #28 OR #29 OR # 30 OR # 31 OR # 32 OR # 33 OR # 34 OR # 35 OR # 36 OR # 37 |
| Sum | 39. #10 AND #23 AND #38 |
| String |  |
| Results and date | 1476 results, 15^th^ January 2025. |

**Table 6. ERIC via EBSCOHost**

| PCC criteria | Words |
| --- | --- |
| Population: Well-being economy for people and the planet. | 1. Public Health [ ti] 2. Public Health [ ab] 3. planetary health[ ti] 4. planetary health[ ab] 5. Ecohealth [ ti] 6. Ecohealth [ ab] 7. One Health[ ti] 8. One Health [ ab] 9. Public health [su] 10. #1 OR #2 OR #3 OR #4 OR #5 OR #6 OR #7 OR #8 OR #9 |
| Concept: Competencies to perform well-being economy | 11. Well* economy [ ti] 12. Well* economy [ ab] 13. Well* capitals [ ti] 14. Well* capitals [ ab] 15. Economic well* [ ti] 16. Economic well* [ab] 17. Human well* [ ti] 18. Human well* [ab] 19. Planetary well* [ti] 20. Planetary well* [ab] 21. Social well* [ti] 22. Social well* [ab] 23. #11 OR #12 OR #13 OR #14 OR #15 OR #16 OR #17 OR #18 OR #19 OR #20 OR #21 OR #22 |
| Context | 24. Competency framework*[ ti] 25. Competency framework*[ab] 26. Knowledge [ ti] 27. Knowledge[ab] 28. Atittude*[ ti] 29. Atittude*[ab] 30. Skill*[ ti] 31. Skill*[ab] 32. Value*[ ti] 33. Value*[ab] 34. competency based education[su] 35. professional competence [su] 36. #24 OR #25 OR #26 OR #27 OR #28 OR #29 OR # 30 OR # 31 OR # 32 OR # 33 OR # 34 OR # 35 |
| Sum | 37. # 10 AND #23 AND #36 |
| String |  |
| Results and date | 56, 15^th^ January 2025. |
