# Supplementary material for: Towards a competency framework for wellbeing economy in public health: a scoping review
Source: Public Health Rev. 2026 Aug 5;47:1609947. doi: 10.3389/phrs.2026.1609947 (PMC13500908; doi:10.3389/phrs.2026.1609947)
Supplement: Supplementary file 5 [file Supplementaryfile5.docx]

**Supplementary material 5.** Towards a Competency Framework for Public Health and Wellbeing Economy: Data Extraction

**Extraction: Template**

1. Reference.
2. Contact author.
3. Aim.
4. Focus: Well-being economy or its capitals.
5. Framework: title.
6. Framework: Structure.
7. Interpretation & conceptualization: Frameworks and domains
8. Tables: Frameworks (domains, subdomains and/or competencies).
9. Financial support.
10. Conflict of interest.

**First: Unit of analysis.**

1. **Reference:** Organisation for Economic Cooperation and Development.(2025). Working Party on Artificial Intelligence Governance Assessing potential future artificial intelligence risks, benefits and policy imperatives Perspectives from the OECD Expert Group on AI Futures.
2. **Contact author:** Not provided.
3. **Aim: “**This initiative aims to equip governments with insights to craft forward-looking AI (artificial intelligence) policies. This report discusses research and expert insights on prospective AI benefits, risks, and policy imperatives. While offering guidance for policymakers, decision-makers are encouraged to remain aware of uncertainties, actively seek diverse perspectives and vigilantly monitor the societal implications of AI innovations.”
4. **Focus:** “Through ranking and synthesis of these, as discussed in Annex A, it put forth ten priority benefits that warrant policy focus: 1. accelerated scientific progress, such as through devising new medical treatments; 2. better economic growth, productivity gains and living standards; 3. reduced inequality and poverty, aided through poverty reduction efforts and improved agriculture; 4. better approaches to address urgent and complex issues, including mitigating climate change and advancing other Sustainable Development Goals (SDGs); 5. better decision-making, sense-making and forecasting through improved analysis of present events and future predictions; 6. improved information production and distribution, including new forms of data access and sharing; 7. better healthcare and education services, such as tailored health interventions and tutoring; 8. improved job quality, including by assigning dangerous or unfulfilling tasks to AI; 9. empowered citizens, civil society and social partners, including through strengthened participation; 10. improved institutional transparency and governance, instigating monitoring and evaluation.”
   a. Human well-being.
   b. Social well-being.
   c. Economic well-being.
   d. Planetary well-being.
5. **Framework(title).** The OECD skills for Artificial Intelligence.
6. **Framework (Structure).** Two competencies.
7. **Framework (Interpretation/ conceptualization):** Note: interpretations for the competencies are not provided.
8. **Table 1.** The OECD skills for Artificial Intelligence.

**( TOTAL FRAMEWORKS: 1) and** ( **TOTAL DOMAINS: 0)**

| **#** | **Competencies** | **Overall competencies** |
| --- | --- | --- |
| 1 | The skills needed to develop, adopt, and use artificial intelligence. | *1* |
| 2 | Artificial intelligence literacy skills. | *2* |

9. **Financial support:** Not provided
10. **Conflict of interest:** Not provided

**Second: Unit of analysis.**

1. **Reference:** Armijo, E., & Asnake, T. (2025). The Effects of Early Childhood Development (ECD) Programs on the Socio-emotional Well-being of Children and Caregivers in Refugee and Forced Displacement Settings. Education Working Paper; No. 6, November 2024. © Washington, DC: World Bank. http://hdl.handle.net/10986/42756 License: [CC BY-NC 3.0 IGO](https://creativecommons.org/licenses/by-nc/3.0/igo).”
2. **Contact author:** The World Bank, Email: [AskEd@worldbank.org](mailto:AskEd@worldbank.org)
3. **Aim:** “This paper examines the effect of Early Childhood Development (ECD) programs on the psychosocial and socio emotional well-being of forcibly displaced caregivers and children between the ages of 0 and 8 years through a systematic literature review and key informant interviews”.
4. **Focus:** “psychosocial and socio emotional well-being”. Two capitals were identified:
   a. Human well-being (Mental health & well‑being and early childhood development)
   b. Social well-being: (Living in safety & free from violence)
5. **Framework (Title):** Armijo´s framework.
6. **Framework (Structure):** One domain and competencies.
7. **Framework (Interpretation/ conceptualization):**
   a. “Socioemotional well-being is a state characterized by positive emotions, positive relationships, and the ability to manage challenging emotions and situations. The components of socioemotional well being for young children include”:
      i. “Self-regulation: The ability to manage emotions and behaviors, such as calming oneself down when upset or staying focused on a task. Social competence: The ability to form and maintain positive relationships with others, such as making friends and sharing”.
      ii. “Emotional understanding: The ability to understand and respond to the emotions of others, such as empathy and compassion”.
   b. Psychosocial Well-being: Note (the document does not define it).
8. **Table**: Armijo´s framework.

**(TOTAL FRAMEWORKS:2) and ( Total domains 1)**

| # | Domain | **Overall competencies** |
| --- | --- | --- |
| **Domain: Socioemotional and Psychosocial Well-being** | | |
|  | Competencies |  |
| 1 | Resilience. | *3* |
| 2 | Empathy. | *4* |
| 3 | Cooperation. | *5* |
| 4 | Self-awareness. | *6* |
| 5 | Self-regulation. | *7* |
| 6 | Social competence. | *8* |
| 7 | Problem-solving. | *9* |
| 8 | Emotional regulation. | *10* |

9. **Financial support:** Not provided.
10. **Conflict of interest:** Not provided.

**Third: Unit of analysis.**

1. **Reference:** Lee, J., & Ž. Žarnic. (2024). The impact of digital technologies on well-being: Main insights from the literature *OECD Papers on Well-being and Inequalities*, No. 29, OECD Publishing, Paris, <https://doi.org/10.1787/cb173652-en>.
2. **Contact author:** The Organization for Economic Cooperation and Development ,well-being@oecd.org.
3. **Aim:** “This paper reviews the literature to better understand their impact on individual and societal well-being, building on a field of research that, while still in its infancy, is growing rapidly”.
4. **Focus:** “Some of the literature focuses on specific well-being outcomes, such as quality and quantity of sleep, eye strain, depression and anxiety, perceived social isolation, and attention-deficit or hyperactivity disorder”. “Policy work, on the other hand, refers to digital well-being in a broader context, emphasising the wide range of implications that digital technologies can have for people’s life and societal well-being. For instance, (OECD, 2019[6]) considers the impacts of technology through a prism of the OECD Well-Being Framework (Figure 1), assessing how it is affecting eleven key dimensions of people’s well-being (i.e. income and wealth, jobs and earnings, housing, health status, education and skills, work-life balance, civic engagement and governance, social connections, environmental quality, personal security and subjective well-being)”. The four capitals were identified
   a. Human well-being: Mental health & well‑being.
   b. Social well-being: Social cohesion & embracing diversity and building trust in others & in institutions.
   c. Economic well-being: Living wage.
   d. Planetary well-being: Good air & water quality and healthy & sustainable living environment.
5. **Framework(title):** Lee et al´s framework
6. **Framework (Structure):** One domain and three competencies
7. **Framework (Interpretation/ conceptualization):**
   a. “Digital literacy: to establish trust based on people’s ability to verify the credibility of online content and information sources”.
   b. “The spread of mis- and dis-information poses risks to the well-being of people and society, and can contribute to polarisation, jeopardise the implementation of policies, and undermine trust in democratic institutions and processes”.
   c. “Critical digital literacy: referring to both the ability to evaluate online content and the knowledge of the potential benefits and limitations of Internet for civic life, can facilitate civic engagement.”
8. **Table:** Lee et al´s framework

**(TOTAL FRAMEWORKS:3) and ( TOTAL DOMAINS: 2)**

| **#** | **Domain and competencies** | **Overall competencies** |
| --- | --- | --- |
| **Domain: Digital literacy** | | |
| Competency  1 | The ability to sort fact from fiction. | *11* |
| Competency  2 | To recognise mis- and dis-information. | *12* |
| Competency  3 | Critical digital literacy. | *13* |

9. **Financial support:** Not provided
10. **Conflict of interest:** Not provided

**Fourth: Unit of analysis.**

1. **Reference:** Sangha, KK., Dinku, Y., Costanza, R., & Poelina, A. (2024). A comprehensive analysis of well-being frameworks applied in Australia and their suitability for Indigenous peoples. *International journal of qualitative studies on health and well-being*, *19*(1), 2321646. <https://doi.org/10.1080/17482631.2024.2321646>
2. **Contact author:** Kamaljit K Sangha, Kamaljit.Sangha@cdu.edu.au
3. **Aim:** “We review the existing key wellbeing frameworks applied in Australia both for the wider public and Indigenous peoples. The aim is to provide a comprehensive overview of various applied frameworks, along with a critical analysis of domains or dimensions comprising those frameworks, and to analyse the role of nature in those frameworks.”
4. **Focus:** “covering social, economic, health, cultural and spiritual dimensions of human living”. “On an individual scale, wellbeing includes having good health and being well-off in key aspects of life such as health, family life, work, housing, culture and leisure. At a broader societal scale, wellbeing comprises the social, cultural, economic, natural and polity environments that are all interconnected, com-plex and dynamic.”
   a. Human well-being.
   b. Social well-being.
   c. Economic well-being.
   d. Planetary well-being.
5. **Framework(title).** The Australian Treasury’s wellbeing framework.
6. **Framework (Structure).** Five domains. Note: we interpretate them as competencies rather than domains.
7. **Framework (Interpretation/ conceptualization):** Note: extracted from: [Treasury's Wellbeing Framework | Treasury.gov.au](https://treasury.gov.au/publication/economic-roundup-issue-3-2012-2/economic-roundup-issue-3-2012/treasurys-wellbeing-framework)
   a. Competency 1^st^: The set of opportunities available to people. This includes not only the level of goods and services that can be consumed, but good health and environmental amenity, leisure and intangibles such as personal and social activities, community participation and political rights and freedoms.
   b. Competency 2^nd^: The *distribution* of those opportunities across the Australian people. In particular, that all Australians have the opportunity to lead a fulfilling life and participate meaningfully in society.
   c. Competency 3^rd^: The distribution of those opportunities across the Australian people. In particular, that all Australians have the opportunity to lead a fulfilling life and participate meaningfully in society.
   d. Competency 4^th^: The overall level and allocation of *risk* borne by individuals and the community. This includes a concern for the ability, and inability, of individuals to manage the level and nature of the risks they face.
   e. Competency 5^th^: The *complexity* of the choices facing individuals and the community. Our concerns include the costs of dealing with unwanted complexity, the transparency of government and the ability of individuals and the community to make choices and trade-offs that better match their preferences.
8. **Table: The Australian Treasury’s wellbeing framework.**

**(TOTAL FRAMEWORKS:4) and (TOTAL DOMAINS:2)**

| **#** | **Competencies** | **Overall competencies** |
| --- | --- | --- |
| 1 | Level of opportunity and freedom that people enjoy: capacity for people to choose the lives they want to live. | *14* |
| 2 | Level of consumption possibilities: people’s command over resources to obtain goods and services to satisfy their needs and wants. | *15* |
| 3 | Distribution of those consumption possibilities: spread of all aspects of consumption possibilities across the population, including across different groups in society, across different geographic regions and across generations. | *16* |
| 4 | Level of risk that people are required to bear: intrinsic uncertainty in possible outcomes that is present in almost all decisions. In the broadest conceptual sense, risk impacts on all individuals and is everywhere in the economy and in society. | *17* |
| 5 | Level of complexity that people are required to deal with. Proliferation of the number of considerations, and the interconnections between those considerations for many economic and broader social decisions. | *18* |

9. **Financial support:** This work was conducted under a fellowship offered by the Australian Studies Institute, Australian National University, Canberra.
10. **Conflict of interest:** No potential conflict of interest was reported by the author(s).

**Fifth: Unit of analysis.**

1. **References:**
   a. Burtscher, M., S. Piano., & B. Welby. (2024). Developing skills for digital government: A review of good practices across OECD governments, *OECD Social, Employment and Migration Working Papers*, No. 303, OECD Publishing, Paris, <https://doi.org/10.1787/f4dab2e9-en>.
   b. European Commission: Joint Research Centre, Vuorikari, R., Kluzer, S., & Punie, Y. (2022). *DigComp 2.2, The Digital Competence framework for citizens : with new examples of knowledge, skills and attitudes*, Publications Office of the European Union. <https://data.europa.eu/doi/10.2760/115376>
2. **Contact author:**
   a. Reference a: Magdalena Burtscher: magdalena.burtscher@oecd.org, Stefano Piano: [stefano.piano@oecd.org](mailto:stefano.piano@oecd.org)
   b. Reference b: Name: Yves Punie, Yves.Punie@ec.europa.eu
3. **Aim:** “Digital technologies are having a profound impact on economies, labour markets and societies. They also have the potential to transform government, by enabling the implementation of more accessible and effective services. To support a shift towards digital government, investment is needed in developing the skills of civil servants. This paper reviews good practices across OECD countries to foster skills for digital government.”
4. **Focus:** “Digital technologies are having a profound impact on economies, labour markets and societies. They also have the potential to transform government, by enabling the implementation of more accessible and effective services. To support a shift towards digital government, investment is needed in developing the skills of civil servants. This paper reviews good practices across OECD countries to foster skills for digital government”. “he overall conclusion from this body of work is that governments should proactively invest in skills, training, and education to make the most of the digital transformation, rather than mitigate the impact of technology on workers, society, and economies. A comprehensive adult learning strategy is needed to adapt to a changing world of work and to ensure that all individuals, particularly the most vulnerable, have adequate opportunities for upgrading their skills throughout their careers. Within such a strategy, it is important to address digital skills gaps. Individuals with stronger digital skills are better able to access digital services and use digital technologies in the workplace, which contributes to better career prospects and higher levels of well-being. Digital skills can also play a major role in supporting the green transition, for example by facilitating the acquisition of environmental competences”. Four capitals are included:
   a. Human well-being.
   b. Social well-being.
   c. Economic well-being.
   d. Planetary well-being.
5. **Framework(title).** Note: this review offers diverse frameworks
6. **Framework (Structure).**
   a. European Digital Competence Framework for Citizens ( DigComp): Five domains and 21 competencies.
   b. Syllabus project in Italy provides a particularly interesting application of DigCom: Five domains and 11 competencies.
   c. The Model of Digital Skills by the Government Digital Academy in Denmark: Four domains, 13 subdomains, and 32 competencies. Note: the document does not provide abbreviations list: IT is not defined in the document.
7. **Framework (Interpretation/ conceptualization):**
   a. Information and data literacy: Note (no definition provided).
   b. Communication & collaboration: Note (no definition provided).
   c. Digital content creation: Note (no definition provided).
   d. Safety: The last two areas are “transversal” as safety and problem solving apply to any type of activity carried out using digital technology.
   e. Problem solving: The last two areas are “transversal” as safety and problem solving apply to any type of activity carried out using digital technology.
   f. Data, information, and digital documents: Note (no definition provided).
   g. Communication and sharing: Note (no definition provided).
   h. Safety: Note (no definition provided).
   i. Online services: Note (no definition provided).
   j. Digital transformation: Note (no definition provided).
   k. Strategy and business development: Note (no definition provided).
   l. Projects & further development: Note (no definition provided).
   m. Data & security: Note (no definition provided).
   n. Governance and co-operation: Note (no definition provided).
8. **Table : Frameworks	(TOTAL FRAMEWORKS:8) and (TOTAL DOMAINS:16)**
   a. **Table a. DigComp framework.**

| **#** | **Domains and competencies** | **Overall competencies** |
| --- | --- | --- |
| Domain 1: Information and data literacy. | |  |
| Competency  1 | Browsing, searching, filtering data, information & content. | *19* |
| Competency  2 | Evaluating data, information, and digital content. | *20* |
| Competency  3 | Managing data, information, and digital content. | *21* |
| **Domain 2: Communication & collaboration.** | | |
| Competency  4 | Interacting through digital technologies. | *22* |
| Competency  5 | Sharing through digital technologies. | *23* |
| Competency  6 | Engaging citizenship through digital technologies. | *24* |
| Competency  7 | Collaborating through digital technologies. | *25* |
| Competency  8 | Netiquette. | *26* |
| Competency  9 | Managing digital identity. | *27* |
| **Domain 3: Digital content creation.** | | |
| Competency  10 | Developing digital content. | *28* |
| Competency  11 | Integrating and re-elaborating digital content. | *29* |
| Competency  12 | Copyright and licences. | *30* |
| Competency  13 | Programming. | *31* |
| **Domain 4: Safety.** | | |
| Competency  14 | Protecting devices. | *32* |
| Competency  15 | Protecting personal data and privacy. | *33* |
| Competency  16 | Protecting health and well-being. | *34* |
| Competency  17 | Protecting the environment. | *35* |
| **Domain 5: Problem solving.** | | |
| Competency  18 | Solving technical problems. | 36 |
| Competency  19 | Identifying needs and technological responses. | 37 |
| Competency  20 | Creatively using digital technology. | 38 |
| Competency  21 | Identifying digital competence gaps. | 39 |

b. **Table b: Syllabus project in Italy provides a particularly interesting application of DigCom**

| **#** | **Domains and competencies** | **Overall competencies** |
| --- | --- | --- |
| **Domain 1: Data, information, and digital documents.** | | |
| Competency  1 | Manage data, information, and digital content. | 40 |
| Competency  2 | Produce, evaluate, and manage computerised documents. | 41 |
| Competency  3 | Learn about Open Data. | 42 |
| **Domain 2: Communication and sharing.** | | |
| Competency  4 | Communicate and share within the administration. | 43 |
| Competency  5 | Communicate and share with citizens, businesses, and other public administrations. | 44 |
| **Domain 3: Safety.** | | |
| Competency  6 | Protect devices. | 45 |
| Competency  7 | Protect personal data and privacy. | 46 |
| **Domain 4: Online services.** | | |
| Competency  8 | Know the concept digital identity and its use to access digital public services. | 47 |
| Competency  9 | Deliver online services. | 48 |
| **Domain 5: Digital transformation.** | | |
| Competency  10 | Know the goals of digital transformation. | 49 |
| Competency  11 | Know the emerging technologies for digital transformation. | 50 |

c. **Table C. DigComp 2.2:** **Key Competences for Lifelong Learning**

| **#** | **Competencies** | **Overall competencies** |
| --- | --- | --- |
| 1 | Literacy competence: the abilities to distinguish and use differ ent types of sources, to search for, collect and process information. | 51 |
| 2 | Cultural awareness and expression. | 52 |
| 3 | Entrepreneurship. | 53 |
| 4 | Civic competence. | 54 |
| 5 | Personal, social, and learning to learn. | 55 |
| 6 | Digital competence. | 56 |
| 7 | Science, technology, engineering, and mathematical. | 57 |

d. **Table d: The Model of Digital Skills by the Government Digital Academy in Denmark**

| **#** | **Domains, subdomains, and competencies** | **Overall competencies** |
| --- | --- | --- |
| **Domain 1: Strategy and business developmen** | | |
| **Subdomain 1:**  **Strategy formulation.** | | |
| Competency 1 | Formulate strategies and action plans. | 58 |
| Competency 2 | Determine financial implications and potentials. | 59 |
| **Subdomain 2:**  **Legislation and technology.** | | |
| Competency 3 | Ensure the link between legislation and digitalisation. | 60 |
| Competency 4 | Know the potential of new and existing technologies. | 61 |
| Competency 5 | Know the common public IT solutions and digital public architecture. | 62 |
| **Subdomain 3:**  **Identification of potential and need.** | | |
| Competency 6 | Involve users and stakeholders by continuously identifying and testing needs. | 63 |
| Competency 7 | Determine the digital potential of the core public functions. | 64 |
| Competency 8 | Map and model business processes. | 65 |
| Competency 9 | Integrate business and IT. | 66 |
| **Domain 2: Projects & further development.** | | |
| **Subdomain 4:**  **Specification of requirements.** | | |
| Competency 10 | Formulate user stories, acceptance criteria and requirements specification. | 67 |
| Competency 11 | Use, evaluate and implement different forms of tenders and contracts. | 68 |
| Competency 12 | Assess implications for administrative law. | 69 |
| **Subdomain 5:**  **Development and optimisation.** | | |
| Competency 13 | Be able to implement government IT projects. | 70 |
| Competency 14 | Ensure the quality of digital solutions. | 71 |
| Competency 15 | Apply relevant development and operational methods. | 72 |
| Competency 16 | Be able to further develop and optimise operations. | 73 |
| **Subdomain 6:**  **Organisational implementation.** | | |
| Competency 17 | Practice change management. | 74 |
| Competency 18 | Ensure continuous operation. | 75 |
| **Domain 3: Data & security.** | | |
| **Subdomain 7:**  **Cyber and information security.** | | |
| Competency 19 | Act cyber safely. | 76 |
| Competency 20 | Act in accordance with GDPR. | 77 |
| Competency 21 | Acting in accordance with ISO 27001. | 78 |
| **Subdomain 8:**  **Use of data.** | | |
| Competency 22 | Use data to develop core public functions. | 79 |
| Competency 23 | Create an overview of data and data flows. | 80 |
| **Subdomain 9:**  **Data ethics and administration.** | | |
| Competency 24 | Know ethical issues and assess legal issues when working with data. | 81 |
| **Domain 4: Governance and co-operation.** | | |
| **Subdomain 10:**  **Supplier co-operation and management.** | | |
| Competency 25 | Be able to manage and collaborate with suppliers. | 82 |
| Competency 26 | Know the IT market and be able to assess its offerings. | 83 |
| **Subdomain 11:**  **Financial management.** | | |
| Competency 27 | Manage IT costs. | 84 |
| Competency 28 | Manage project finances. | 85 |
| Competency 29 | Ensure the attainment of returns. | 86 |
| **Subdomain 12:**  **Governance and portfolio management.** | | |
| Competency 30 | Map and analyse the systems landscape. | 87 |
| Competency 31 | Manage the IT portfolio through its life cycle. | 88 |
| Competency 32 | Ensure adequate governance, IT organisation and skills. | 89 |

9. **Financial support:**
   a. **Reference a:** The paper is based on a joint project on “Strengthening the administrative capacity of the National Institute for Social Security in Italy (INPS)" between the OECD, the European Commission and INPS.”
   b. **Reference b:** This publication is a Science for Policy report by the Joint Research Centre (JRC), the European Commission’s science and knowledge service.
10. **Conflict of interest:**
    a. **Reference a:** Not provided.
    b. **Reference b:** Not provided.

**Sixth: Unit of analysis.**

1. **References:**

- Del Rio, M., Lasley, P., Tallon, L., Kauth, J.-M., Bare, G., McCurdy, L.E., & Etzel, R.A. ( 2024).Performance indicators corresponding to the critical competencies in children's environmental health. Journal of Environmental Health. *Journal of Environmental Health*,86(10):24-29.
- Del Rio, M., Lasley, P., Tallon, L., Kauth, J.-M., Bare, G., McCurdy, L.E., & Etzel, R.A. (2023). Critical competencies in children’s environmental health. *Journal of Environmental Health*, 85(6), 26–29.

2. **Contact author:** Michelle Del Rio, Email: midelrio@iu.edu.
3. **Aim**: “Despite the value of children’s environmental health, there are still gaps in workforce training for those interested in children’s environmental health. These gaps in knowledge and training highlight the need for improved ways to build the capacity of children’s environmental health professionals. Our work focused on creating a set of competencies for public health professionals interested in children’s environmental health careers as a way to meet the demand for children’s environmental health specialists.”
4. **Focus:** “Competency in children’s environmental health allows for the development of interventions that can prevent the long-term and irreversible health outcomes that result from early environmental toxic exposures. Health e• ects that are thought to be at least partially infl uenced by early exposures include cardiovascular disease, diabetes, cancer, autism, attention-defi cit/hyperactivity disorder (ADHD), lower IQ, Parkinson’s disease, and Alzheimer’s disease.”
   a. Human well-being.
   b. Planetary well-being.
5. **Framework(title)**: Critical Competencies in Children’s Environmental Health.
6. **Framework (Structure):** 12 competencies.
7. **Framework (Interpretation/ conceptualization):** Not provided
8. **Table** Critical Competencies in Children’s Environmental Health.
11. **(TOTAL FRAMEWORKS: 9 ) and (TOTAL DOMAINS:16)**

| **#** | **Competencies** | **Overall competencies** |
| --- | --- | --- |
| 1 | Assess a children’s environmental health concern, risk, or potential exposure in a community and develop a briefing paper. | *90* |
| 2 | Present information to stakeholders about children’s environmental health threats and prevention methods. | *91* |
| 3 | Develop, implement, and evaluate a community-based intervention to mitigate a children’s environmental health threat. | *92* |
| 4 | Increase children’s exposure to healthy natural environments. | *93* |
| 5 | Monitor and report child health indicators to the state or local public health department. | *94* |
| 6 | Communicate to the media promoting children’s environmental health through traditional and nontraditional outlets (e.g., social media). | *95* |
| 7 | Identify how climate change and environmental exposures (e.g., pesticides) affect children’s health (short and long term). | *96* |
| 8 | Be able to recognize or assess structural and systemic harms (e.g., built environment, climate change, risks associated with exposure) on children’s health. | *97* |
| 9 | Identify federal, state, and local regulations as they relate to children’s health and the environment. | *98* |
| 10 | Prepare and present testimony about children’s health and the environment before local and state legislators. | *99* |
| 11 | Identify actions and evaluate yearly progress toward the reduction of greenhouse gas emissions and the carbon footprint of an organization (i.e., state or local health department). | *100* |
| 12 | Design environmental health guidelines that account for children’s unique vulnerabilities and long-term susceptibility to health effects. | *101* |

9. **Financial support:** Not provided.
10. **Conflict of interest:** Not provided.

**Seventh: Unit of analysis.**

1. **References:** Doshmangir, L., Alipouri Sakha, M., Mostafavi, H., Kabiri, N., Ghaffarifar, S., & Takian, A. (2024). Essential core competencies for health policy graduates: a multi-method consensus type study. *Health research policy and systems*, *22*(1), 136. <https://doi.org/10.1186/s12961-024-01221-8>.
2. **Contact author:** Leila Doshmangir Doshmangirl@tbzmed.ac.ir; Minoo Alipouri Sakha sakhaminoo@gmail.com; alipourisakha.d@iums.ac.ir Hakimeh Mostafavi hakimeh_mostafavi@yahoo.com Neda Kabiri ne.kabiry@gmail.com.
3. **Aim**: “The objective of this study is to identify the skills and competencies required for health policy graduates to prepare them for a spectrum of future roles, including both academic and non-academic positions. The findings presented herein may assist policy-makers and educational planners in Iranian higher education institutions in developing competency-based health policy curricula, thus enabling them to respond to the evolving societal needs pertaining to the sustainable development of health in Iran, and possibly in other settings.”
4. **Focus:** “In light of the multi-faceted challenges confronting health systems worldwide and the imperative to advance towards development goals, the contribution of health policy graduates is of paramount importance, facilitating the attainment of health and well-being objectives”.
   a. Human well-being.
5. **Framework(title)**: The core competencies for health policy doctoral graduates. Note: Authors identified other frameworks based on a review of literature.
6. **Framework (Structure):** Each framework has a different structure.
7. **Framework (Interpretation/ conceptualization):** Note: Domains were not defined.
   a. Domain 1: Health policy research: Note (no definition provided).
   b. Domain 2: Policy analysis: Note (no definition provided).
   c. Domain 3: Educational competencies: Note (no definition provided).
   d. Domain 4:Decision-making: Note (no definition provided).
   e. Domain 5: Multidisciplinary work: Note (no definition provided).

8. **Table. (TOTAL FRAMEWORKS: 19) and (TOTAL DOMAINS:21)**
   a. Table a. The core competencies for health policy doctoral graduates

| **#** | **Domains, subdomains and competencies** | **Overall competencies** |
| --- | --- | --- |
| **Domain 1: Health policy research.** | | |
| **Subdomain 1:**  **Identification, access and evaluation of evidence to identify research gaps in health and healthcare systems.** | | |
| Competency 1 | Exploring databases and journals to retrieve relevant articles and documents. | 102 |
| Competency 2 | Reviewing documents by critically appraising scientific work and determine gaps for further investigation. | 103 |
| Competency 3 | Assessing status quo to identify research needs and priorities. | 104 |
| Competency 4 | Developing hypotheses and appropriate interventions. | 105 |
| Competency 5 | Formulating research questions relevant to health policy. | 106 |
| Competency 6 | Interpreting results and identifying strengths and limitations of research. | 107 |
| **Subdomain 2:**  **Designing and implementation of basic and advanced research in health policy.** | | |
| Competency 7 | Transforming research question into a well-designed study. | 108 |
| Competency 8 | Evaluating strengths and weaknesses of experimental, quasi-experimental and observational studies. | 109 |
| Competency 9 | Ability to use advanced research methods in the design, implementation and interpretation of independent research projects. | 110 |
| Competency 10 | Using applied knowledge of qualitative and quantitative paradigms in the design of research, including data collection and analysis techniques such as content analysis. | 111 |
| Competency 11 | Using applied knowledge of inferential and descriptive statistical analysis and its application in health-related research. | 112 |
| Competency 12 | Ability to prepare and analyse big data, use conceptual frameworks to conduct research related to health policy. | 113 |
| Competency 13 | Capability to select and implement appropriate analytical techniques from advanced epidemiological, statistical, economic, qualitative and survey methods (e.g. structured systematic review, meta synthesis and meta-analysis, causal models, logistic and multiple linear regression, general linear models, longitudinal and multilevel models, cost-effectiveness analysis, cost–benefit analysis and cost utility analysis) for a specific research question. | 114 |
| Competency 14 | Ability to develop and evaluate research proposals and policy summaries, interpret the results of studies related to health policy and present findings in both research and operational contexts. | 115 |
| Competency 15 | Capability to determine, evaluate and specify most appropriate sources of data for a specific research question; select valid and reliable data, analyse the validity and reliability of data; and collect valid and reliable qualitative and quantitative data. | 116 |
| Competency 16 | Ability to use information technology, both in data gathering and analysis. | 117 |
| **Subdomain 3:**  **Application of ethical principles to research.** | | |
| Competency 17 | Consider social and cultural values and prioritize societal needs. | 118 |
| Competency 18 | Avoiding scientific distortion and misleading evidence in research endeavours. | 119 |
| Competency 19 | Observing ethical principles in treatment of subjects to achieve research objectives. | 120 |
| **Subdomain 4:**  **Translation and effective presentation of findings.** | | |
| Competency  20 | Presenting study findings, orally or in written form, to various ranges of stakeholders. | 121 |
| Competency 21 | Ability to publish research evidence. | 122 |
| **Subdomain 5:**  **Identifying and examining the policies and problems.** | | |
| Competency 22 | Determine, describe and analyse health-related problems by critically comparing and applying the theories of policy process in the study of health-related problems | 123 |
| Competency 23 | Having a good understanding of public health paradigms (national and international) | 124 |
| Competency 24 | Understanding the process and evolution of national health policies and how they are related to the policies of other health systems around the world | 125 |
| **Domain 2: Policy analysis** | | |
| **Subdomain 6:**  **Analysing and interpreting policies.** | | |
| Competency 25 | Ability to describe the stages of the policy process, including problem identification, policy formulation, implementation and evaluation. | 126 |
| Competency 26 | Identify, describe, analyse and evaluate issues and policies related to the health care system and public health using tools and models of policy-making and policy analysis. | 127 |
| Competency 27 | Identify the key sectors, institutions and stakeholders involved in the policy process at the national, regional and local levels. | 128 |
| Competency 28 | Critique the policy process, including the central role of rules, regulations and lawsuits; the role of stakeholders; the difference between national laws and local policies; and the effect of academic research on the policy process. | 129 |
| Competency 29 | Capability to collect and analyse data for health policy analysis; apply ethical principles to health policy-making; extract and interpret the results of policy formulation process, identify inputs and deter mine the effectiveness of agendas; and determine and predict potential outcomes of health policy strategies. | 130 |
| **Domain 3: Educational competencies.** | | |
| **Subdomain 7:**  **Education- theory and practice.** | | |
| Competency 30 | Ability to perform educational needs assessment; use curriculum review models; apply teaching principles, techniques and styles; implement teaching and learning principles; develop lesson plans and course plans; develop educational content; manage and lead educational activities; perform strategic and operational planning in education; use and develop modern technologies in education; effectively communicate and motivate learners; assess teaching performance; assess the performance of learners; evaluate curricula; manage courses and classrooms; apply concepts related to other specialized disciplines; and review curricula and educational scholarships. | 131 |
| **Domain 4: Decision-making.** | | |
| **Subdomain 8:**  **Leadership and management.** | | |
| Competency 31 | Ability to successfully manage organizations and promote public health; lead the process of determining priorities and allocating resources necessary for achieving organizational goals. | 132 |
| Competency 32 | Ability to interpret and aggregate the findings from qualitative and quantitative data in operational projects and programmes of the organization. | 133 |
| Competency 33 | Ability to determine goals and objectives of programmes; and use evidence to inform decision making. | 134 |
| Competency 34 | Considering current and future trends (financial, social, political and health-related trends) in developing and monitoring strategic plans for the organization. | 135 |
| Competency 35 | Adopting methods to minimize internal and external barriers to pro vision of services by analysing the barriers, and the ways forward. | 136 |
| Competency 36 | Capable to select best options among policies, programmes and services; and manage programmes within the boundaries of current and future budgets. | 137 |
| Competency 37 | Effectively manage the implementation of policies and procedures of government agencies overseeing the organizations. | 138 |
| Competency 38 | Developing performance management systems– Interacting with other health-related organizations at the national, regional and global levels. | 139 |
| Competency 39 | Collecting relevant information to select policy options, pro grammes and strategic plans. | 140 |
| Competency 40 | Participate in the development of new strategies and policies that help adjust or improve current policies. | 141 |
| Competency 41 | Developing evidence-based policies using scientific data and consider all ethical and legal considerations as well as the interests of various stakeholders. | 142 |
| Competency 42 | Use the results of applied research and evaluation methods to implement strategic public health and healthcare plans more effective. | 143 |
| Competency 43 | Develop strategic plans to improve public health (describe measurable outcomes, make decisions about necessary changes in policy and appoint groups and individuals responsible for implementing these plans). | 144 |
| Competency 44 | Ability to apply the principles of strategic planning, program development and performance measurement to public health initiatives that utilize effective partnerships between organizations and com munities– Develop policies to expand international cooperation. | 145 |
| **Subdomain 9:**  **Evaluation.** | | |
| Competency 45 | Assess the health needs of a population and provide strategies for solving health problems at various organizational and societal levels. | 146 |
| Competency 46 | Use appropriate and accurate methods to predict the outcomes of health policies as well as general policies that affect health, including biological and epidemiological statistics, mathematical techniques and simulations. | 147 |
| Competency 47 | Analyse key concepts such as health indicators, patient demand for healthcare services and health financing, with emphasis on the critique of the effects of alternative financing methods and organization of healthcare services on costs, quality, access and public health. | 148 |
| Competency 48 | Evaluate the main components of the policy-making process that affect the functions of health systems, including: financing, stewardship, resource generation and delivery of healthcare services. | 149 |
| Competency 49 | Use proper and accurate empirical methods to evaluate the financial, political, legal and/or geographical factors influencing health. | 150 |
| Competency 50 | Perform cost-effectiveness and cost–benefit analysis of innovative healthcare initiatives and public health policies. | 151 |
| Competency 51 | Assess the performance and composition of institutions, actors and processes in the development of public health policies. | 152 |
| Competency 52 | Evaluate the role of public health policy-making at the national, regional and international levels. | 153 |
| Competency 53 | Evaluate the organization and financing of public health and medical services, and critique their impact on accessibility, utility and quality of healthcare, costs and outcomes. | 154 |
| Competency 54 | Critically evaluate the effect of health policies on organization, provision and financing of public health services. | 155 |
| Competency 55 | Analyse the access and utilization of healthcare services, costs, outcomes and performance of the health system using the results of applied research. | 156 |
| Competency 56 | Analyse financial interactions within the health system and their impact on organizations and stakeholders. | 157 |
| Competency 57 | Evaluate structures, duties, powers and responsibilities in public health organizations. | 158 |
| Competency 58 | Collect information for evaluating strategic policies and plans. | 159 |
| Competency 59 | Analyse the applications of strategic policies, plans and programmes. | 160 |
| Competency 60 | Use financial analysis methods in decision-making about strategic policies, plans, and programmes. | 161 |
| Competency 61 | Assess the financial, political, legal and geographical feasibility of policies, plans and programmes. | 162 |
| Competency 62 | Ability to identify, develop and select strategic policies, plans and programmes; use health informatics in evaluation of strategic policies, plans and programmes; and employ appropriate and accurate techniques in evaluation of policy option to recommend appropriate policy action. | 163 |
| **Domain 5: Multidisciplinary work.** | | |
| **Subdomain 10:**  **Consultation.** | | |
| Competency 63 | To create teams to achieve strategic goals and plans; write a memorandum of understanding where goals are clearly defined; effectively communicate to promote public health; create opportunities for cooperation with different organizations with the goal of promoting public health. | 164 |
| Competency 64 | To establish bilateral relations with stake holders, policymakers and related groups; interact with other health organizations at the local, regional and international levels. | 165 |
| Competency 65 | To cooperate with government agencies that have the power and authority to influence public health. | 166 |
| Competency 66 | To use evidence-based models of communication and negotiation to disseminate findings; establish compelling and information-based communications with various health policy audiences and professionals. | 167 |
| Competency 67 | To consult other health specialists and policymakers to formulate innovative public plans and programmes. | 168 |
| **Subdomain 11:**  **Advocacy.** | | |
| Competency 68 | To advocate for the use of evidence in decision-making; engage in policies and programmes outside the health sector; provide public health financing mechanisms. | 169 |
| Competency 69 | To justify budgets and programmes; obtain information for the formulation of contracts and agreements. | 170 |
| Competency 70 | To negotiate contracts and agreements. | 171 |
| Competency 71 | To explain financial analysis techniques (cost-effectiveness, cost–benefit, cost-utility and return on capital) for strategic policies, plans and programmes. | 172 |

b. Johns Hopkins University´s framework.

| **#** | **Competencies** | **Overall competencies** |
| --- | --- | --- |
| 1 | Public health policy process. | 173 |
| 2 | Critical comparison of policy-making theories. | 174 |
| 3 | Knowing the main stakeholders in policymaking of national, regional and local levels. | 175 |
| 4 | Criticizing the policy-making process. | 176 |
| 5 | Assessing organization of public health services. | 177 |
| 6 | Critical analysis of literature and gap analysis for further research | 178 |
| 7 | Knowing research methodologies. | 179 |
| 8 | Assessing health policies using statistical analysis, epidemiology and surveys. | 180 |
| 9 | Knowledge translation. | 181 |

c. University of Kansas´ framework

| **#** | **Competencies** | **Overall competencies** |
| --- | --- | --- |
| 1 | Healthcare-related research skills. | 182 |
| 2 | Ability to define significant research questions. | 183 |
| 3 | Designing studies, data analysis, data collection and managing data. | 184 |
| 4 | Knowledge translation. | 185 |

d. The George Washington University´s framework

| **#** | **Competencies** | **Overall competencies** |
| --- | --- | --- |
| 1 | Critical assessment of political, legal, philosophical, economical and social aspects of health policies . | 186 |
| 2 | Critical recognition of theoretical knowledge in support of health policy research, choosing appropriate research designs. | 187 |
| 3 | Understanding and analysis of health systems and payment models and provision and assessment on health inequalities. | 188 |
| 4 | Critical assessment of political issues. | 189 |
| 5 | Policy analysis. | 190 |
| 6 | Research abilities in health policies. | 191 |

e. Harvard University´s framework.

| **#** | **Competencies** | **Overall competencies** |
| --- | --- | --- |
| 1 | Knowledge of theories of political participation, legislative organization, formation of interest groups and political communication. | 192 |
| 2 | Applied topics including public opinion, political ideology, public health law and media. | 193 |
| 3 | Knowledge in the field of interaction between the executive, legislative and judicial branches; and the role of federalism, including state and local government, in influencing health policy outcomes. | 194 |
| 4 | Research methods include quantitative statistical methods suitable for large-scale databases, survey methods and qualitative or mixed methods. | 195 |
| 5 | Teaching. | 196 |
| 6 | Conducting research on healthcare policies. | 197 |
| 7 | Collaboration with government, professional groups in research projects related to public policies in the fields of public health and health services. | 198 |

f. McMaster University´s.

| **#** | **Competencies** | **Overall competencies** |
| --- | --- | --- |
| 1 | Health human resources, economic evaluation of policy options, public and private financing of health care, social investments in health production. | 199 |
| 2 | Analysis of the political aspects of health policy including the effects of political institutions, actors, values and ideas that operate in the spheres of state and global power. | 200 |
| 3 | Analysis of social influences leading to the production of health (and disease), the organization of services and health systems and policies related to them. (Production and use of information to inform policies, political economy of health and its production, social factors of health). | 201 |
| 4 | Methods of research and analysis of health policies by qualitative and quantitative methods and by theoretical and practical methods. | 202 |
| 5 | Integration of theoretical knowledge and practical skills to solve health policy problems. | 203 |

g. Swansea University´s framework

| **#** | **Competencies** | **Overall competencies** |
| --- | --- | --- |
| 1 | Creating, interpreting, analysing and developing knowledge through research. | 204 |
| 2 | Dissemination of new knowledge obtained through original research– Applying research skills and thematic theory in research. | 205 |
| 3 | Ideation, design and implementation of projects with the aim of creating new knowledge or programmes in health policies. | 206 |
| 4 | Making informed judgments about complex health policy issues. | 207 |
| 5 | Adhering to the correct ethical principles in research, respecting the integrity of individuals and in accordance with the codes of professional conduct. | 208 |
| 6 | Appropriate response to unforeseen problems in project design by making appropriate modifications. | 209 |
| 7 | Communicate complex research findings clearly, effectively to expert audiences using a variety of media and appropriate events, including conference presentations, seminars and workshops. | 210 |
| 8 | Correct selection, interpretation and application of relevant techniques for research and advanced scientific research. | 211 |
| 9 | Creating networks and bases of research and development in this field– Advanced research skills. | 212 |
| 10 | Use of information in research. | 213 |
| 11 | Exercising personal responsibility and initiative in complex and unpredictable situations in professional environments. | 214 |

h. Temple University´s framework.

| **#** | **Competencies** | **Overall competencies** |
| --- | --- | --- |
| 1 | Familiarizing with health policy theories, comparative health policy, public health policies and legal issues, current and emerging issues in public health and health professions. | 215 |
| 2 | Research method: biostatistics, and research methods in health policies. | 216 |
| 3 | Advocacy for public health. | 217 |

i. The Pennsylvania State University´s framework.

| **#** | **Competencies** | **Overall competencies** |
| --- | --- | --- |
| 1 | Research on the organization and delivery of health services. | 218 |
| 2 | Health care financing and policy: topics covered include various arrangements for financing and paying for healthcare, current issues in healthcare payment and reimbursement and other public healthcare finance issues. | 219 |
| 3 | Research methods in health services research. | 220 |
| 4 | Health data analysis for research. | 221 |

j. University of Illinois´s framework.

| **#** | **Competencies** | **Overall competencies** |
| --- | --- | --- |
| 1 | Knowledge in the field of health services research and its role in the public policy process and providing theoretical frameworks, data sources, research methods and basic findings on the major issues of health services research, access, quality and cost, financing and effectiveness of health services. | 222 |
| 2 | The ability to apply the philosophy of science and theories and models related to health services research. | 223 |
| 3 | Organization, development and financing of the health service system and its components and the definition of health and its broader determinants. | 224 |
| 4 | Advanced statistical methods suitable for studying secondary data. | 225 |
| 5 | Cost–benefit analysis in the evaluation of health programmes and policies. | 226 |
| 6 | Program evaluation and policy analysis including secondary analysis of available datasets. | 227 |
| 7 | Designing public health research. | 228 |
| 8 | Economic evaluation of healthcare interventions. | 229 |

9. **Financial support:** This study was funded by Tabriz University of Medical Sciences, Tabriz, Iran (No: 59805& 67490)
10. **Conflict of interest:** The authors declare no competing interests.

**Eight: Unit of analysis.**

1. **References:** Powell, N., Dalton, H., Lawrence-Bourne, J., & Perkins, D. (2024). Co-creating community wellbeing initiatives: what is the evidence and how do they work? *International Journal of Mental Health Systems*, *18*(1). <https://doi.org/10.1186/s13033-024-00645-7>.
2. **Contact author:** Hazel Dalton, hdalton@csu.edu.au.
3. **Aim**: The research questions are as follows:1. Can community wellbeing initiatives, with wellbeing as a stated objective, be identified that have some implementation success as measured by (i) duration of existence (at least two years) and (ii) have published evidence regarding the initiative (e.g. peer-reviewed article)? 2. For the chosen community wellbeing initiatives: a What was the context for initiation? b. Which stakeholders were involved and what were their roles in the successful implementation? c. What was done to promote community wellbeing by these initiatives? d. How was momentum sustained and progress measured? e. What were the implementation and outcome lessons that may be used by other communities?
4. **Focus:** “Wellbeing has multiple aspects including: physical, mental, intellectual, social, emotional and spiritual components. As discussed above, in this review, we use the wellbeing definition as postulated in Ryan’s theory of self-determination, which stands in the positive mental health domain”. “Moreover, place is where things happen, such as natural disasters, acute economic insults such as the closure of local enterprises, and suicide clusters”. “Community is where people live, it is the environment that shapes their wellbeing. In short, “community” concerns the level of analysis and “well-being” describes the scope of analysis”
   a. Human well-being.
   b. Social well-being.
   c. Economic well-being.
   d. Planetary well-being.
5. **Framework(title)**: Framework for community wellbeing initiatives – key principles and processes.
6. **Framework (Structure):** The framework has eight domains ( principles) and competencies that are distributed into three steps: initiation and planning, implementation, continuation and sustainability.
7. **Framework (Interpretation/ conceptualization):**
   a. Community alignment – align with community needs, strengths, and history – adapt to context: The community initiatives included were sensitive to the context in which they operated. During initiation and planning, the collection of subjective and objective data enabled a contextual understanding of community need.
   b. Community ownership – encourage bottom-up energy to build community ownership: The first step to generate community ownership, was to keep the community voice and vision as the anchor point for all planning and leadership.
   c. Engaged community – include community in strategy and action to build trust: Five initiatives brought the community together to discuss the future, which was key to their planning and visioning, and may have played a role in engaging community members in leadership or working group positions.
   d. Shared purpose – establish based on a collective understanding of wellbeing: A shared vision that reflected the community voice and aligned with the local context was an important factor in the organisation of initiatives.
   e. Collaborative action – form and maintain relationships and partnerships: The selected initiatives had a locally based, collaborative leadership team.
   f. Transparent communication – openly communicate with community and partners, including promotion activities: Active communication between the initiative and the broader community was used to engage the community for initial discussion; to involve members as leaders, volunteers, or champions to advertise the purpose and vision of the initiative; to publicise the plan; to advertise sponsored or organised activities; and to list key community contacts for support or involvement.
   g. Continuous learning – monitor and evaluate activities: Each initiative adapted over time as they learned how to operate and be effective.
   h. Resource management – secure and use resources flexibly: Several of the initiatives were described from the perspective of the funders, making it challenging to assess the financial resource dimension. Some initiatives were established only as funding was secured; others secured funding as they went along. Funding was often discussed, including receipt or application for funding and how relationships with funders were managed.
8. **Table.** Framework for community wellbeing initiatives – key principles and processes

**(TOTAL FRAMEWORKS: 20) and (TOTAL DOMAINS:29)**

| # | Domain, subdomains and competencies | **Overall competencies** |
| --- | --- | --- |
| **Domain 1: community alignment.** | | |
| **Subdomain 1: Initiation and planning.** | | |
| Competency 1 | Discover what people want. | 230 |
| Competency  2 | Discover what data say. | 231 |
| **Subdomain 2: Initiation and planning.** | |  |
| Competency  3 | Support aligned partner activities. | 232 |
| Competency  4 | Avoid duplication. | 233 |
| **Subdomain 3: continuation and sustainability.** | | |
| Competency  5 | Review if needs and assets have changed. | 234 |
| **Domain 2: community ownership.** | | |
| **Subdomain 1: Initiation and planning.** | | |
| Competency  6 | Keep community central in planning and leadership through representation. | 235 |
| **Subdomain 2: Initiation and planning.** | | |
| Competency  7 | Build capacity of champions and volunteers to empower community. | 236 |
| **Subdomain 3: continuation and sustainability.** | | |
| Competency  8 | Increase the opportunities for community to contribute in ways that suit them. | 237 |
| **Domain 3: Engage community.** | | |
| **Subdomain 1: Initiation and planning.** | | |
| Competency 9 | Bring community together to discuss future. | 238 |
| **Subdomain 2: Initiation and planning.** | | |
| Competency 10 | Use a variety of activities and networks to engage community. | 239 |
| **Subdomain 3: continuation and sustainability.** | | |
| Competency 11 | Tangible action and change will bring in people. | 240 |
| **Domain 4: Shared purpose.** | | |
| **Subdomain 1: Initiation and planning.** | | |
| Competency 12 | Form a shared worthwhile vision. | 241 |
| Competency 13 | Codesign clear goals and plans. | 242 |
| Competency 14 | Commit to the long-term. | 243 |
| **Subdomain 2: Initiation and planning.** | | |
| Competency 15 | Match activities to the overall purpose. | 244 |
| **Subdomain 3: Continuation and sustainability.** | | |
| Competency 16 | Be comfortable that the long-term purpose can sustain the initiative through highs and lows. | 245 |
| **Domain 5: Collaborative action.** | | |
| **Subdomain 1: Initiation and planning.** | | |
| Competency 17 | Establish collaborative leadership and governance. | 246 |
| Competency 18 | Seek assistance as needed. | 247 |
| **Subdomain 2: Initiation and planning.** | | |
| Competency 19 | Leverage parent strengths in collaborative activities | 248 |
| **Subdomain 3: continuation and sustainability.** | | |
| Competency 20 | Advocate for the community with governance | 249 |
| **Domain 6: Transparent communication.** | | |
| **Subdomain 1: Initiation and planning.** | | |
| Competency 21 | Use media and networks to build awareness and bring in community. | 250 |
| **Subdomain 2: Initiation and planning.** | | |
| Competency 22 | Develop a coherent narrative. | 251 |
| Competency 23 | Communicate links between activities and purpose. | 252 |
| **Subdomain 3: continuation and sustainability.** | | |
| Competency 24 | Celebrate short-term success. | 253 |
| Competency 25 | Keep promoting the long-term vision. | 254 |
| **Domain 7: Continuous learning.** | | |
| **Subdomain1: Initiation and planning.** | | |
| Competency 26 | Record how decisions and actions are made. | 255 |
| **Subdomain 2: Initiation and planning.** | | |
| Competency 27 | Monitor and evaluate activities. | 256 |
| Competency 28 | Learn from success and failure. | 257 |
| Competency 29 | Solve problems. | 258 |
| Subdomain:3 continuation and sustainability. | |  |
| Competency 30 | Review progress and evolve. | 259 |
| **Domain 8: Resource management.** | | |
| **Subdomain1: Initiation and planning.** | | |
| Competency 31 | Bring in people and organisations with appropriate networks and resources. | 260 |
| Competency 32 | Apply for funding. | 261 |
| **Subdomain 2: Initiation and planning.** | | |
| Competency 33 | Bring in people with sets of skills as champions and volunteers. | 262 |
| Competency 34 | Use small grants for specific activities. | 263 |
| **Subdomain 3: continuation and sustainability.** | | |
| Competency 35 | Use history of action and success to secure further. | 264 |
| Competency 36 | Funding and partner commitment. | 265 |

9. **Financial support:** We acknowledge seed funding for the scoping review which formed the foundational concepts of this paper was provided by the NSW Mental Health Commission. From 2018 to 2021, NP, HD, JLB and DP were employed by the University of Newcastle Centre for Rural and Remote Mental Health, with core funding support from NSW Health, Mental Health Branch. HD acknowledges support for completing and publishing this paper via a grant from the Commonwealth of Australia, represented by the Department of Health (Grant Activity 4-DGEJZ1O/4-CW7UT14).
10. **Conflict of interest:** The authors declare no competing interests.

**Nineth: Unit of analysis.**

1. **References:** Sebong, P. H., Pardosi, J., Goldman, R. E., Suryo, A. P., Susianto, I. A., & Meliala, A. (2024). Identifying Physician Public Health Competencies to Address Healthcare Needs in Underserved, Border, and Outer Island Areas of Indonesia: A Rapid Assessment. Teaching and learning in medicine, 1–12. Advance online publication. <https://doi.org/10.1080/10401334.2024.2353573>.
2. **Contact author:** Perigrinus h. Sebong, falconperin_consultan@yahoo.com.
3. **Aim**: “the present study aims to identify the knowledge and skills that doctors require to practice successfully in remote areas of Indonesia, particularly in the DTPK areas.”
4. **Focus:** “Public health and medical education are complementary, as both seek to improve the health and well-being of individuals and communities.” “To overcome these conditions, Indonesian medical schools have emphasized the vital linkage between medical education and the public health landscape, i.e., the socio-demographic and geo graphic variation that influences healthcare systems and outcomes. Most are integrating community- based education and health systems topics into their curricula, including social determinants of health, healthcare policy and financing, interprofessional education, patient-centered care, quality improvement, and systems thinking”
   a. Human well-being.
   b. Social well-being.
   c. Economic well-being.
5. **Framework(title)**: Sebong et al´framework.
6. **Framework (Structure):** list of competencies.
7. **Framework (Interpretation/ conceptualization):** Competencies are not conceptualized.
8. **Table.** Sebong et al´ framework.

**(TOTAL FRAMEWORKS: 21 ) and (TOTAL DOMAINS:29)**

| **#** | **Competencies** | **Overall competencies** |
| --- | --- | --- |
| 1 | Appropriate response to natural emergencies with inadequate resources. | 266 |
| 2 | Advocate for and promote healthcare coordination in mitigation plans and during disasters. | 267 |
| 3 | Knowledgeable on disease surveillance systems at the border areas, particularly community-based surveillance, and surveillance from another institution (e.g., veterinary agency and immigration). | 268 |
| 4 | to recognize and consider the fundamental factors required for an effective local health facilities strategy, such as medical logistics planning, program monitoring, and evaluation. | 269 |
| 5 | to capture messages or related issues in the community regarding the routine health program implementation at the Puskesmas. | 270 |
| 6 | Leadership to preventive and promotional approaches within interprofessional collaboration. | 271 |
| 7 | Management functions, especially those relevant to program-based health services. | 272 |
| 8 | Allocating resources and strategic management. | 273 |
| 9 | Systems thinking. | 274 |
| 10 | Advocate with evidence-based support. | 275 |
| 11 | Exposure to national health systems and knowing how to apply systems thinking to solve clinical and health problems. | 276 |

9. **Financial support:** No external funding supported this research or the manuscript’s preparation.
10. **Conflict of interest:** No potential conflict of interest was reported by the author(s).

**Tenth: Unit of analysis.**

1. **References:**
   a. Scottish Government. (2024). Wellbeing economy toolkit: supporting place based economic strategy and policy development. <https://www.gov.scot/binaries/content/documents/govscot/publications/advice-and-guidance/2022/11/wellbeing-economy-toolkit-supporting-place-based-economic-strategy-policy-development/documents/wellbeing-economy-toolkit-supporting-place-based-economic-strategy-policy-development/wellbeing-economy-toolkit-supporting-place-based-economic-strategy-policy-development/govscot%3Adocument/wellbeing-economy-toolkit-supporting-place-based-economic-strategy-policy-development.pdf>
   b. Scottish Government. n.d. Scotland’s National Performance Framework Our Purpose, Values and National Outcomes <https://nationalperformance.gov.scot/>
   c. Scottish Government. n.d. Local Wellbeing Economy Monitor: Guidance. <https://view.officeapps.live.com/op/view.aspx?src=https%3A%2F%2Fwww.gov.scot%2Fbinaries%2Fcontent%2Fdocuments%2Fgovscot%2Fpublications%2Fadvice-and-guidance%2F2022%2F11%2Fwellbeing-economy-toolkit-supporting-place-based-economic-strategy-policy-development%2Fdocuments%2Fwellbeing-economy-monitor-guidance-updated-february-2024%2Fwellbeing-economy-monitor-guidance-updated-february-2024%2Fgovscot%253Adocument%2Fwellbeing-economy-monitor-guidance-updated-february-2024.docx&wdOrigin=BROWSELINK>
2. **Contact author:** Scottish Government, Email: NSET@gov.scot.
3. **Aim**: “To provide an illustration of this toolkit in practice, a case study from our Wellbeing Economy Pilot project with Clackmannanshire Council has been included”.
4. **Focus:** “The Scottish Government is working to make the transition to a wellbeing economy that is based on four principles: prosperity, equality, sustainability and resilience.”

Well-being economy as a unit.

5. **Framework(title)**: Scotland’s National Performance Framework.
6. **Framework (Structure):** the framework contains 11 competencies.
7. **Framework (Interpretation/ conceptualization):** The competencies are not defined.
8. **Table.** Scotland’s National Performance Framework.

**(TOTAL FRAMEWORKS: 22 ) and (TOTAL DOMAINS:29)**

| **#** | **Competencies** | **Overall competencies** |
| --- | --- | --- |
| 1 | Taking an open, transparent, participatory approach to developing strategy and policy, involving those impacted by the policy, empowering citizens and diverse communities. | 277 |
| 2 | Setting a clear purpose and vision for economic policy and activity, focused on collective wellbeing. | 278 |
| 3 | Establishing clear outcomes and metrics for measuring if and how our economy is delivering wellbeing for people, place and planet. | 279 |
| 4 | Taking an evidence-based (qualitative and quantitative). | 280 |
| 5 | Whole-systems view to understand the key drivers of wellbeing outcomes, how they interrelate, and which have the greatest impact. | 281 |
| 6 | Adopting a preventative approach by focusing interventions on upstream parts of the system to avoid negative impacts on outcomes downstream and building long term resilience. | 282 |
| 7 | Embedding inclusion, equality and fairness into economic policy from the outset.  . | 283 |
| 8 | Working with and empowering communities and citizens to gain a greater stake in the economy and removing barriers to participation. | 284 |
| 9 | Monitoring, evaluating and encouraging continuous learning, being open to innovative and experimental approaches. | 285 |
| 10 | To treat people with kindness, dignity and compassion, to respect the rule of law, and to act in an open, transparent way. | 286 |
| 11 | To value, enjoy, protect, and enhance our environment. | 287 |

**9. Financial support:** Not provided.

**10. Conflict of interest:** Not provided.

**Eleventh: Unit of analysis.**

1. **References:** Simon, J., Parisi, S., Wabnitz, K., Simmenroth, A., & Schwienhorst-Stich, E.-M. (2023). Ten characteristics of high-quality planetary health education—Results from a qualitative study with educators, students as educators and study deans at medical schools in Germany. Frontiers in Public Health, 11. <https://doi.org/10.3389/fpubh.2023.1143751>.
2. **Contact author:** EvaMaria Schwienhorst, Stich schwienhor_e@ukw.de.
3. **Aim**: “The climate and ecological crises are considered fundamental threats to human health. Healthcare workers in general and doctors in particular can contribute as change agents in mitigation and adaptation. Planetary health education (PHE) aims to harness this potential. This study explores perspectives among stakeholders involved in PHE at German medical schools on the characteristics of high-quality PHE and compares them to existing PHE frameworks.”
4. **Focus:** “An ethical discussion of issues such as gender-based discrimination, racism, sexism, capitalism, ), and modern medical treatments and their impact on the environment (VS34) should be included. “Social justice” in terms of a “global justice perspective and the different regional impacts of environmental change also need to be discussed”. “We believe that the potential of Indigenous knowledge systems for addressing the ecological crises as well as sensitization of learners to the shared drivers of these crises and the marginalization of Indigenous communities and others groups-which can be subsumed under the labels of settler-colonialism, Eurocentrism and extractive economic practices- should be part of PHE.”
   a. Human well-being.
   b. Social well-being.
   c. Economic well-being.
   d. Planetary well-being.
5. **Framework(title)**: Transformative competencies including practical skills.
6. **Framework (Structure):** A framework with ten domains, but only four contains competencies. The other domains are: Inter- and trans disciplinarity, Special role of student, Need for curricular integration, Innovative and proven didactic methods, and education as a driver of innovation.
7. **Framework (Interpretation/ conceptualization):**
   a. Complexity and system thinking : Many participants stressed the importance of a solid foundation of factual knowledge that takes the complexity of the topic into account and at the same time introduces a perspective of systems thinking.
   b. Ethical dimension: An ethical discussion of issues such as gender-based discrimination, racism, sexism, capitalism, and modern medical treatments and their impact on the environment should be included. “Social justice” in terms of a “global justice perspective” and the different regional impacts of environmental change also need to be discussed.
   c. Responsibility of health professionals: A reflection of one’s own position in society as well as options for action or even agency, including the role of a change agent as a healthcare professional, should be part of planetary health education (PHE).
   d. Transformative competencies including practical skills: By acquiring knowledge about possible actions and reflecting about their own options for action.
   e. Space for reflection and resilience building: Learners need enough space for guided and unguided reflection inside and outside the classroom.
8. **Table. Transformative competencies including practical skills**

**(TOTAL FRAMEWORKS: 23 ) and (TOTAL DOMAINS:34)**

| **#** | **Domains and Competencies** | **Overall competencies** |
| --- | --- | --- |
| **Domain 1: Complexity and system thinking.** | | |
| Competency 1 | “Factual knowledge” on climate and environmental changes, knowledge on environmental factors as health determinants, such as urban planning and the psychosocial environment and a historical context of the development of human societies to date were considered important. | 288 |
| Competency 2 | Students should be enabled to understand and classify the multiple complex interrelationships of the system and the interaction of these interrelationships with human health (VS17 19) as well as to consider patients in their entire environment. | 289 |
| **Domain 2: Ethical dimension.** | | |
| Competency 3 | to reflect from a climate justice perspective. | 290 |
| Competency 4 | to think globally and act locally. | 291 |
| **Domain 3: Responsibility of health professionals.** | | |
| Competency 5 | recognize and learn to use their special role as agents in the health care system. | 292 |
| **Domain 4: Transformative competencies including practical skills.** | | |
| Competency 6 | Become change agents. | 293 |
| Competency 7 | Gain confidence in their own agency. | 294 |
| Competency 8 | Motivating the implementation of own ideas. | 295 |
| Competency 9 | Science competencies, including searching, finding, correctly classifying, and evaluating sources of scientific evidence is relevant in order to be able to communicate knowledge properly and also to apply it to one’s own actions. | 296 |
| Competency 10 | To acquire and practice communicative skills ) by talking to other groups of society. | 297 |
| **Domain 5: Space for reflection and resilience building.** | | |
| Competency 11 | to reflect and discuss freely and to share frustrating experiences. | 298 |
| Competency 12 | to question one’s own behavior and values, to promote “systemic thinking, critical thinking” to “broaden the horizon” and to reflect on the learning journey. | 299 |
| Competency 13 | To incorporate resilience building for learners as they are confronted with dire future scenarios. | 300 |

**9. Financial support:** This publication was supported by the OpenAccessPublication Fund of the University of Wüerzburg.

**10. Conflict of interest:** The authors declare that the research was conducted in the absence of any commercial or financial relationships that could be construed as a potential conflict of interest.

**Twelfth: Unit of analysis.**

1. **References:** World Health Organization European Region. (2023). Country deep dive on the well-being economy: Iceland. Copenhagen: WHO Regional Office for Europe. Licence: CC BY-NC-SA 3.0 IGO [WHO-EURO-2023-7415-47181-69111-eng.pdf](https://iris.who.int/bitstream/handle/10665/369105/WHO-EURO-2023-7415-47181-69111-eng.pdf?sequence=1).
2. **Contact author:** World Health Organization Regional Office for Europe, Email: eurocontact@who.int.
3. **Aim**: “the aim to demonstrate concrete country experiences in advancing and implementing well-being economies”.
4. **Focus:** well-being economy as a unit.
5. **Framework(title)**: WHO´s competencies for well-being economy: Iceland.
6. **Framework (Structure):** Note: the document does not provide a reference for the competencies.
7. **Framework (Interpretation/ conceptualization):** Note: the document does not provide a reference for the competencies.
8. **Table.** WHO´s competencies for well-being economy: Iceland.

**(TOTAL FRAMEWORKS: 24 ) and (TOTAL DOMAINS:34)**

| **#** | **Competencies** | **Overall competencies** |
| --- | --- | --- |
| 1 | Socioemotional learning. | 301 |
| 2 | Democracy and citizenship. | 302 |
| 3 | Health literacy. | 303 |

**9. Financial support:** Not provided

**10. Conflict of interest:** Not provided

**Thirteenth: Unit of analysis.**

1. **References:** Organisation for Economic Cooperation and Development. (2023), *OECD Skills Outlook 2023: Skills for a Resilient Green and Digital Transition*, OECD Publishing, Paris. <https://doi.org/10.1787/27452f29-en>.
2. **Contact author:** Not provided
3. **Aim**: “This edition of the Skills Outlook brings a range of new, important insights into changes in the demand for skills resulting from the green and digital transition and the role skills policies play in ensuring resilience.”
4. **Focus:** “Climate change and policy to combat it, as well as digital transformation, are the defining challenges of our time. The urgency to address climate change has never been more apparent. The consequences of rising temperatures are profound and unforgiving. Extreme weather events, raging wildfires, biodiversity loss, natural disasters and the resulting food and water insecurity are becoming increasingly commonplace. These changes are not isolated. They trigger a domino effect of economic disruption, adverse health effects, heightened conflict and forced migration. At the same time, progress in artificial intelligence (AI) and, in particular, the onset of generative AI is such that, in some areas, its output has become indistinguishable from that of humans, and in several domains, AI’s capabilities are well beyond what humans can do. The AI revolution is redefining our workplaces, societies and knowledge exchange mechanisms. While AI systems bear the promise of supporting scientific discoveries that could enhance the health, productivity and well-being of many, they could also challenge many jobs and even be used to spread false and misleading information, eroding public trust, threatening security and social cohesion.”
   a. Human well-being.
   b. Social well-being.
   c. Economic well-being.
   d. Planetary well-being.
5. **Framework(title)**: Skills for a resilient green and digital transition.
6. **Framework (Structure):** Some tables have domains and competencies.
7. **Framework (Interpretation/ conceptualization):**
   a. Resilient green and digital transition: Reorienting the economy so that economic growth occurs within planetary boundaries and ensuring that rapid technological developments do not create new social and economic vulnerability may change the set of tasks individuals will have to perform. Such change is likely to require individuals to rely on a renewed set of skills in their work and everyday life. As a result, education and training systems will have to adapt to align skills development opportunities with emerging skills needs in labour markets and society more widely.
   b. The green transition and how skills policies can promote resilience: Reorienting the economy to create an environmentally sustainable future requires the adoption of environmentally sustainable production and consumption practices.
   c. The digital transition and how skills policies can promote resilience: Recent technological innovations have profoundly changed how information is collected, stored, used, generated and exchanged. Supporting further technological innovations while ensuring that technological developments enhance the economic well-being of broad populations rather than only a few, and addressing the possible negative effects of new technologies on health, well-being and social cohesion.
   d. Strengthen a wider set of skills and attitudes AI professionals need to be able to develop ethical and trustworthy AI systems: The continuous evolution of AI systems brings with it different skills needed by AI workers to develop, adapt, and maintain AI applications, as well as different attitudes populations should have to live and work in the age of AI.
   e. Health literacy: Health literacy encompasses the knowledge, motivation and skills required to access, comprehend, assess and apply information to make informed decisions regarding healthcare, disease prevention and the enhancement of overall well-being over the life course.
   f. Socio-emotional and communication skills: These skills are critical to building a resilient green and digital transition because these are uniquely human skills and, therefore, not easily automatable even with advances in Artificial intelligence in the near future. Moreover, the collective effort needed to promote the green transition rests on the capacity of communities to negotiate conflict and pool collective expertise to solve complex systemic problems.
   g. Raising awareness about the role of metacognitive skills: metacognitive skills allow individuals to recognise the role of heuristics and biases in shaping decision making and make more informed use of knowledge and information. Metacognitive skills, in fact, empower individuals by helping them characterise knowledge and knowledge generation processes and can help individuals effectively work alongside others – whether humans or machines. They help individuals and communities solve complex problems and develop reasonable risk mitigation strategies both at individual and societal levels.
8. **Tables. (TOTAL FRAMEWORKS: 28) and (TOTAL DOMAINS:37)**
   a. Skills for a resilient green and digital transition

| **#** | **Domains and competencies.** | **Overall competencies** |
| --- | --- | --- |
|  |  |  |
| **Domain 1: Health literacy.** | | |
| Competency  1 | Ability to search and obtain health information ( Access). | 304 |
| Competency  2 | Ability to evaluate and interpret health information (Appraise). | 305 |
| Competency  3 | Ability to make sense of the health information they have at their disposal ( Understand). | 306 |
| Competency  4 | Ability to communicate and use such information to make an informed decision in order to maintain and improve health. | 307 |
| **Domain 2: socio-emotional and communication skills.** | | |
| Competency  5 | the capacity to collaborate with others, manage emotions, persevere in difficult circumstances and effectively communicate with diverse groups enable individuals to effectively navigate their social environments and make responsible decisions. | 308 |
| **Domain 3: Raising awareness about the role of metacognitive skills.** | | |
| Competency  6 | the ability to think about and regulate one’s own thinking processes, monitor and evaluate one’s own learning and understanding, and plan, set goals, and adjust strategies to meet those goals. | 309 |

b. The green transition and how skills policies can promote resilience

| **#** | **Competencies** | **Overall competencies** |
| --- | --- | --- |
| 1 | Strengthening the environmental sustainability competence of individuals. | 310 |
| 2 | Ensuring that achieving ambitious climate objectives does not lead to labour market vulnerability. | 311 |
| 3 | Developing the range of skills and attitudes needed to adapt to new environmental conditions. | 312 |

c. The digital transition and how skills policies can promote resilience

| **#** | **Domains and Competencies** | **Overall competencies** |
| --- | --- | --- |
| **Domain 1 :Identifying emerging skills needs to more effectively operate in information-rich societies and work alongside emerging generative Artificial intelligence (AI) systems.** | | |
| Competency 1 | Integrating media literacy in education and training programmes. | 313 |
| Competency 2 | the ability to think about and regulate one’s own thinking processes, monitor and evaluate one’s own learning and understanding, and plan, set goals, and adjust strategies to meet those goals. | 314 |
| Competency 3 | Fostering public understanding and awareness of AI technologies and the skills to operate alongside emerging technologies. | 315 |
| **Domain 2: Promoting health literacy to protect communities from infodemics.** | | |
| Competency 4 | Promoting the development of health literacy throughout the lifecycle. | 316 |
| Competency 5 | Improving the accessibility of health-related information by enhancing the communication skills of healthcare providers. | 317 |
| Competency 6 | Addressing healthcare providers’ biases through de-bias training. | 318 |
| Competency 7 | Promoting the administration of health literacy surveys. | 319 |

d. Strengthen a wider set of skills and attitudes AI professionals need to be able to develop ethical and trustworthy AI systems

| **#** | **Competency** | **Overall competencies** |
| --- | --- | --- |
| 1 | Promoting the development of skills to develop, adapt and maintain AI tools and applications. | 320 |
| 2 | Including ethical aspects of AI development and use in education and training programmes leading to AI jobs. | 321 |

**9. Financial support:** Co-funded by the European Union

**10. Conflict of interest:** Not provided

**Fourteenth: Unit of analysis.**

1. **References:** Nykiforuk, C. I. J., Belon, A. P., de Leeuw, E., Harris, P., Allen-Scott, L., Atkey, K., Glenn, N. M., Hyshka, E., Jaques, K., Kongats, K., Montesanti, S., Nieuwendyk, L. M., Pabayo, R., Springett, J., & Yashadhana, A. (2023). An action-oriented public health framework to reduce financial strain and promote financial wellbeing in high-income countries. International Journal for Equity in Health, 22(1). <https://doi.org/10.1186/s12939-023-01877-8>.
2. **Contact author:** Candace I. J. Nykiforuk, candace.nykiforuk@ualberta.ca.
3. **Aim**: “This paper presents the methodology used to develop the Framework, identifies the financial strain and financial wellbeing definitions that are well-aligned with a social deter minants of health (SDoH) agenda, describes the Framework components, specifies target users, and explains the purpose of, and how to use, the Framework”
4. **Focus:** “Perceived financial security impacts physical, mental, and social health and overall wellbeing at community and population levels. Public health action on this dynamic is even more critical now that the COVID‑19 pandemic has exacerbated financial strain and reduced financial wellbeing.”
   a. Human well-being.
   b. Social well-being.
   c. Economic well-being.
5. **Framework(title)**: Action‑Oriented Public Health Framework on Financial Wellbeing and Financial Strain.
6. **Framework (Structure):** The framework has domains, competencies and definitions.
7. **Framework (Interpretation/ conceptualization):**
   a. Government (All Levels): This domain targets structural actions that can be taken by governments through the governing systems of public and private sectors. It refers to macro economic, public, and social policies as well as underlying power structures.
   b. Organizational & Political Culture: This domain targets those processes that affect the delivery and sustainability of government, organizational, and community actions. It involves consideration of organizational culture and power dynamics.
   c. Socioeconomic & Political Context: This domain targets social and political actions. It encompasses changes to the political and community landscape that, together, shape the availability of resources, opportunities for poverty reduction, possibilities for growth of the middle-class, and improvements in the distribution of power at the societal level.
   d. Social & Cultural Circumstances: This domain is about political, community, organizational, and individual actions that shape or recognize social and cultural contexts, hierarchies of power, and people’s social backgrounds and identities (e.g., immigration status, gender, sexual orientation, race/ethnicity) that accumulate to impact their financial circumstances.
   e. Life Circumstances: This domain targets political, community, organizational, and individual actions that impact people’s complex life circumstances, multiple roles, and power relationships (e.g., individual agency and power within a household) that come together – positively or negatively – to shape their financial situation.
8. **Table.** Action‑Oriented Public Health Framework on Financial Wellbeing and Financial Strain.

**(TOTAL FRAMEWORKS: 29) and (TOTAL DOMAINS:42)**

| **#** | **Domains and competencies.** | **Definitions** | **Overall competencies** |
| --- | --- | --- | --- |
| **Domain 1: Government (All Levels)** | | | |
| Compe-tency 1 | Improve Regulation, Oversight, and Funding of Macro‑Economic Systems and Policies. | Increase effectiveness and impact of government regulation and oversight of financial sector, housing market, and employment and labour market. Ensure adequacy of funds for sustainable actions | 322 |
| Compe-tency 2 | Expand Provision, Regulation, and Funding to Care, Education, and Transportation Services. | Ensure governments oversee, regulate, and provide guaranteed level of adequate funding to quality essential care (e.g., childcare, health care), education, and transportation services | 323 |
| Compe-  tency 3 | Prioritize Redistribution‑Based & Universal‑Type Policies. | Build and improve equity‑based policies that redistribute wealth or span the socioeconomic spectrum. Such policies (e.g., progressive taxation, uni versal basic income, and raising minimum retirement pension) dispropor tionately benefit people who experience disadvantage | 324 |
| Compe-  tency 4 | Provide Sustainable Funding to Programs and Services. | Ensure continued and appropriate amount of public financial assistance to support operations and service delivery infrastructure of organizations and governments, targeting areas that directly or indirectly impact people’s f inancial circumstances | 325 |
| **Domain 2: Organizational & Political Culture.** | | | |
| Compe-  tency 5 | Simplify Access to Benefits & Services. | Remove barriers and bureaucratic ‘red tape’ that limit people’s access to benefits, programs, and services, including communication barriers (e.g., low literacy levels), strict contingencies (e.g., work‑for‑welfare), restrictive eligibility criteria, and onerous assessments (e.g., to qualify for disability benefits). | 326 |
| Compe-  tency 6 | Budget for Wellbeing. | Create budgets that prioritize long‑term human wellbeing over financial outcomes alone (e.g., balancing budgets through austerity measures that negatively impact health and overall wellbeing). | 327 |
| Compe -tency 7 | Assess and Measure Long‑Term Impacts. | Use measures of human wellbeing to understand the long‑term impacts of policies, programs, and services (e.g., social impact) Take a long‑term approach to evaluation (e.g., cost–benefit analysis). | 328 |
| **Domain 3: Socioeconomic & Political Context.** | | | |
| Compe-  tency 8 | Expand Access to Financial Services & Products. | Increase access to mainstream and alternative financial services and products that are inclusive, culturally appropriate, affordable (e.g., low‑fee or no‑fee), flexible in terms of contracts and transactions, and responsive to people’s needs and circumstances.  Facilitate access to information about mainstream and alternative financial services and products. | 329 |
| Compe-  tency 9 | Strengthen Employment Security (Income and Benefits). | Improve access to stable, well‑paid, and regulated jobs with employee benefits programs for all workers. | 330 |
| Compe-  tency 10 | Enhance Quality Education. | Facilitate access to education and training to improve people’s long‑term income prospects. | 331 |
| Compe-  tency 11 | Improve Housing Security. | Strengthen affordable housing policies, including high quality options for public housing.  Increase access to diverse affordable and supportive housing options in order to provide people with dignified choices that fit their needs. | 332 |
| Compe-tency 12 | Promote Neighbourhood‑Level Advantage. | employment, safety, and security (e.g., addressing high exposure to the criminal justice system or providing meaningful supports for poorly funded public amenities).  Target family, community, and neighbourhood through multi‑level initiatives to improve local services and supports. | 333 |
| **Domain 4: Social & Cultural Circumstances.** | | | |
| Compe-tency 13 | Include Cultural Values of Financial Practices & Ways of Living. | Recognize and respect the complexity and diversity of cultural values attributed to financial resources (e.g., money, goods) and financial transactions.  Build initiatives that recognize the symbolic and economic values of different ways of being and doing (e.g., pay for informal caregiving) | 334 |
| Compe-tency 14 | Address Stigma & Discrimination (e.g., systemic racism and ableism). | Build initiatives to explicitly reduce stigma and discrimination of groups who experience cumulative disadvantage across the lifespan (e.g., racialized people) and intersecting challenges (e.g., Indigenous woman experiencing disability) in financial services, job markets, and school or workplaces.  Address financial abuse and barriers to both financial independence and intergenerational wealth‑building that disadvantaged groups have systematically experienced. | 335 |
| Compe-tency 15 | Foster Connection & Belonging. | Enhance community capacity, empowerment, and connections through community‑led or participatory approaches promoting social capital and social cohesion. | 336 |
| **Domain 5: Life Circumstances.** | | | |
| Compe-tency 16 | Develop around People’s Everyday Realities. | Remove barriers to enrolment and participation in financial strain and financial wellbeing related initiatives (e.g., access to childcare, transportation costs) .  Ensure the timing and content of the initiatives are tailored to the target populations.  Consider people’s values, life stages, life demands, and daily roles and responsibilities. | 337 |
| Compe-tency 17 | Consider Diverse Household Financial Circumstances. | Create initiatives that are appropriate to people’s current financial circumstances, particularly for people experiencing poverty and facing unmet basic needs (e.g., food insecurity, energy insecurity, housing insecurity) Set realistic, achievable goals (e.g., building savings only after basic needs are addressed). | 338 |

**9. Financial support:** This prject was supported by Canadian Institute of Health Research (CIHR; 2020 COVID‑19 Rapid Research Funding Opportunity; grant #172694). The funder of the study had no role in research design, data collection, data analysis, and interpretation of the findings. The research proposal underwent peer review by the funding body.

**10. Conflict of interest:** The authors declare no competing interests.

**Fifteenth: Unit of analysis.**

1. **References:** Porcelli, A., D’Onise, K., & Pontifex, K. (2023). Public health partner authorities-How a health in all policies approach could support the development of a wellbeing economy. Health Promotion Journal of Australia : Official Journal of Australian Association of Health Promotion Professionals, 34(3), 671–674. <https://doi.org/10.1002/hpja.738>.
2. **Contact author:** Amy Porcelli, amy.porcelli@sa.gov.au.
3. **Aim**: “The literature describes a series of key actions required to sup-port the development of a wellbeing economy. In summary, these include:
   a. Defining wellbeing in the local context and determining what matters to stakeholders and the community.
   b. Developing and communicating a vision for what wellbeing looks like.
   c. Measuring wellbeing and tracking progress to improving wellbeing outcomes.
   d. Aligning priorities, goals and resources to improve wellbeing outcomes
   e. Identifying and prioritising wellbeing economy activities and behaviours”.
4. **Focus:** Well-being economy as a whole.
5. **Framework(title)**: Skills and knowledge required to develop a wellbeing economy.
6. **Framework (Structure):** The framework has domains and competencies.
7. **Framework (Interpretation/ conceptualization):** Note: The article does not define the domains or the competencies.
8. **Table.** Skills and knowledge required to develop a wellbeing economy.

**(TOTAL FRAMEWORKS: 30) and (TOTAL DOMAINS:45)**

| **#** | **Domains and competencies** | **Overall competencies** |
| --- | --- | --- |
| **Domain 1: Soft skills.** | | |
| Competency 1 | Interpersonal skills. | 339 |
| Competency 2 | Development and maintenance of relationships. | 340 |
| **Domain 2: Professional skills.** | | |
| Competency 3 | Stakeholder analysis. | 341 |
| Competency 4 | Community engagement. | 342 |
| Competency 5 | Communication. | 343 |
| Competency 6 | Systems thinking. | 344 |
| Competency 7 | Policy development. | 345 |
| **Domain 3: Subject matter knowledge.** | | |
| Competency 8 | Determinants of health. | 346 |
| Competency 9 | Population health/health promotion approaches. | 347 |
| Competency 10 | Knowledge of the policy environment. | 348 |
| **Domain 4: Technical expertise.** | | |
| Competency 11 | Data/evidence collation and analysis. | 349 |
| Competency 12 | Evaluation design and implementation. | 350 |
| Competency 13 | Economics. | 351 |

**9. Financial support:** N/A.

**10. Conflict of interest:** The authors declare no conflicts of interest.

**Sixteenth : Unit of analysis.**

1. **References:** Betley, E. C., Sigouin, A., Pascua, P., Cheng, S. H., MacDonald, K. I., Arengo, F., Aumeeruddy-Thomas, Y., Caillon, S., Isaac, M. E., Jupiter, S. D., Mawyer, A., Mejia, M., Moore, A. C., Renard, D., Sébastien, L., Gazit, N., & Sterling, E. J. (2023). Assessing human well-being constructs with environmental and equity aspects: A review of the landscape. People and Nature, 5(6), 1756–1773. <https://doi.org/10.1002/pan3.10293>.
2. **Contact author:** Erin C. Betley, ebetley@amnh.org.
3. **Aim**: “In this paper, we report on the results of a search to identify well-being constructs, published in peer-reviewed and grey literature, that consider multiple interacting dimensions of human well-being, including an environmental dimension, as well as equity. To inform future efforts to leverage well-being for public policy, we then assess these well-being constructs through the following research questions: (a) How are well-being constructs that consider environmental dimensions and equity being developed, measured and applied to decision-making? (b) How do well-being constructs with environmental and equity aspects consider environmental dimensions of well-being, and to what extent do these constructs illuminate interrelationships between human well-being and environmental dimensions? and (c) How do well-being constructs with environmental and equity aspects consider equity?”
4. **Focus: “**multiple interacting dimensions of human well-being, including an environmental dimension, as well as equity” “ equity and interrelationships between humans and the environment.” “Well-being is at the core of public policy efforts to engender just, equitable and sustainable futures in a rapidly changing world, especially amidst the increasing frequency of climatic, environmental and economic shocks that affect human well-being. But what constitutes living well, or a good life? While answers to this question vary among individuals and social groups throughout the world, researchers and policymakers have drawn from over 50 years of theory and scholarship on well-being to develop measures that can span mental and physical health, social, cultural, spiritual, economic and environmental dimensions, among others.”
   a. Human well-being.
   b. Social well-being.
   c. Planetary well-being.
   d. Economic well-being.
5. **Framework(title)**: Betley et al´s framework.
6. **Framework (Structure):** A list of competencies.
7. **Framework (Interpretation/ conceptualization):**
   a. Well-being constructs (including frameworks and methods)
8. **Table.** Betley et al´s framework.

**(TOTAL FRAMEWORKS: 31) and (TOTAL DOMAINS:45)**

| **#** | **Competencies.** | **Overall competencies** |
| --- | --- | --- |
| 1 | Sharing different ways that wellbeing is defined and sharing knowledge and expertise about development, measurement and implementation of well-being focused approaches. | 352 |
| 2 | To only assess the discrete dimensions of a construct; rather frameworks must leverage interdisciplinary approaches to assess interactions among the dimensions of well-being, both in concept and in practice, that may produce trade-offs as well as synergies. | 353 |
| 3 | To explore the implications of different ways to measure collective well-being (from aggregation of individualized measurements to broader measures of community or collective well-being) for different components of equity, in particular recognitional equity. | 354 |
| 4 | Constructs should be developed to explicitly centre equity and equity-related categories of social differentiation such as gender when it is present in their efforts (rather than implying it). | 355 |
| 5 | To validate well-being constructs, in part to help ensure equity. | 356 |
| 6 | To share how validated constructs have been integrated into planning, monitoring and/or decision-making. | 357 |
| 7 | To share information on guidelines or metrics relating to equity when the construct is being implemented while accounting for the multidimensionality and intersectional complexity of equity within particular social and cultural contexts. | 358 |
| 8 | To assess the implementation of well-being constructs for improved monitoring, evaluation and learning. | 359 |
| 9 | To assess well-being frameworks and further exploration of how well-being-focused approaches can advance equity in all its dimensions (distributional, procedural, contextual, and recognitional) and will support public policy aimed towards enhancing well-being amidst increasing frequency of global shocks that affect human well-being. | 360 |

**9. Financial support:** Support for this project also comesfrom the Gordon and Betty Moore Foundation, Lynette and RichardJaffe and the Jaffe Family Foundation. SC received support from theInSHS-CNRS program ‘Soutien à la Mobilité Internationale-2018’.Partial support for this work comes from PICS (Projet Internationalde Coopération Scientifique) funded by CNRS-INEE (Institutécologie et environnement) for ‘The Institutional Dynamics ofConversion to Organic Wine Production: A Comparative Analysisin Occitanie, France’, a collaboration between the University ofToronto—Department of Geography and CNRS— Centre d'EcologieFonctionnelle et Evolutive**.**

**10. Conflict of interest:** The authors declare no conflict of interest

**Seventeenth: Unit of analysis.**

1. **References:** Hayman, D. T. S., Barraclough, R. K., Muglia, L. J., McGovern, V., Afolabi, M. O., N’Jai, A. U., Ambe, J. R., Atim, C., McClelland, A., Paterson, B., Ijaz, K., Lasley, J., Ahsan, Q., Garfield, R., Chittenden, K., Phelan, A. L., & Lopez Rivera, A. (2023). Addressing the challenges of implementing evidence-based prioritisation in global health. BMJ Global Health, 8(6). <https://doi.org/10.1136/bmjgh-2023-012450>
2. **Contact author:** Dr David T S Hayman; D.T.S.Hayman@massey.ac.nz.
3. **Aim**: “Global health requires evidence- based approaches to improve health and decrease inequalities. In a roundtable discussion between health practitioners, funders, academics and policy- makers, we recognised key areas for improvement to deliver better- informed, sustainable and equitable global health practices.”
4. **Focus:** Determinants of health and One Health “Multiple factors determine the health of individuals and communities, including location and environment, genetics, income, educational access and other social factors.”
   a. Human well-being.
   b. Social well-being.
   c. Planetary well-being.
   d. Economic well-being.
5. **Framework(title)**: Hayman et al´s framework.
6. **Framework (Structure):** A list of competencies with domains and competencies.
7. **Framework (Interpretation/ conceptualization):**
   a. Determinants of disease and One Health: Multiple factors determine the health of individuals and communities, including location and environment, genetics, income, educational access and other social factors. One Health provides an important overarching framework to work within. While not all global health issues, such as vaccine preventable diseases like measles are seen as direct One Health issues, most health security threats are. One Health aims to reduce the overall threats to global health and its security, through reducing the risk of emergence at its source.
   b. Adaptive and function- based frameworks: Note: not defined.
   c. Evidence-based decision-making: Evidence from research outside the assessment tools, however, must also be considered, for instance operational and implementation research can help decision- making to maximise strategic value of services and systems.
   d. Engagement at multiple levels: Global health systems are complex, where one capacity has direct and indirect impacts on the effectiveness of another, and these may be influenced at local, national, regional and international levels
   e. Communication and misinformation: Prioritisation involves consideration of social context, including engagement of communities, local media, social cohesion and misinformation management. Misinformation, particularly but not only on social media… Transparency in knowledge and information sharing on platforms that are easily accessible to the public may help build trust.
   f. Diversity at the decision- making table: Prioritisation itself needs a good governance model. To create ‘stronger and more inclusive health emergency preparedness, response and resilience architecture’, prioritisation needs to operate within marginal economies, be accountable, transparent, participatory, equitable, consistent with the rule of law and evidence based.
   g. Regional and collaborative entities: Frequently missing in prioritisation discussions is the importance of regional entities. Individual states can benefit from collaborative action with international and NGO groups, including private sector companies, to optimise collective efforts for information sharing, planning, surveillance, financial support, access to equipment and pharmaceuticals, technical training and capacity building.
8. **Table.** Hayman et al´s framework.

**(TOTAL FRAMEWORKS: 32) and (TOTAL DOMAINS:52)**

| **#** | **Domains and competencies** | **Overall competencies** |
| --- | --- | --- |
| **Domain 1: Determinants of disease and One Health.** | | |
| Competency  1 | Consider the determinants of health and mitigating these to reduce threats and improve health. | 361 |
| Competency  2 | Develop systems that strengthen collaboration, communication, capacity building and coordination equally across all sectors responsible for addressing health concerns at the human–animal environment interface. | 362 |
| **Domain 2: Adaptive and function- based frameworks.** | | |
| Competency  3 | Focus on sustainable, cost- effective outcome measures based on function, rather than specific stand- alone capacities. | 363 |
| Competency  4 | Develop adaptive global health security frameworks, incorporating continuous monitoring, evaluation and improvement. | 364 |
| **Domain 3: Evidence-based decision-making.** | | |
| Competency  5 | Draw modelling data from diverse sources and scales and model functional prioritisations for multiple scales. | 365 |
| **Domain 4: Engagement at multiple levels.** | | |
| Competency  6 | Engage with senior government during prioritisation processes. | 366 |
| Competency  7 | Engage with multiple governing sectors from local, national, regional and international. | 367 |
| **Domain 5: Communication and misinformation.** | | |
| Competency  8 | Have transparency in data ownership, modelling, collaboration and information sharing. | 368 |
| Competency  9 | Have respectful and open engagement at all levels of interaction. | 369 |
| Competency  10 | Enhance ‘storytelling’ to the public with improved clarity, visualisation and actionable targets. | 370 |
| **Domain 6: Diversity at the decision- making table.** | | |
| Competency  11 | To include diverse, multisectoral expertise and people in prioritisation discussions and decision- making. | 371 |
| **Domain 7: Regional and collaborative entities.** | | |
| Competency  12 | Develop regional approaches to strategic planning, financing, training, operations and surveillance. | 372 |

**9. Financial support:** None declared.

**10. Conflict of interest:** DTSH and RKB were supported by Bryce Carmine and Anne Carmine (née Percival), through the Massey University Foundation and Percival Carmine Chair; DTSH by Royal Society Te Apārangi RDF- MAU1701; we thank the National Academies of Science, Engineering and Medicine for enabling this collaboration.

**Eighteenth: Unit of analysis.**

1. **References:** Capetola, T., Noy, S., & Patrick, R. (2022). Planetary health pedagogy: Preparing health promoters for 21st‐century environmental challenges. Health Promotion Journal of Australia, 33(Suppl 1), 17–21. https://doi.org/10.1002/hpja.641
2. **Contact author:** Teresa Capetola, teresa.capetola@deakin.edu.au.
3. **Aim**: “The aim of this paper is to offer two case studies for augmenting health promotion skills and competencies to meet ecologically mediated health challenges in the 21st century: the Sustainability Wheel of Fortune (Wheel) and a postgraduate micro credential on planetary health.”
4. **Focus:** Multiple interconnected drivers threaten the health and wellbeing of humans and the environment, including biodiversity loss, climate change, pollution, rapid urbanisation and displacement.
   a. Human well-being.
   b. Social well-being.
   c. Planetary well-being.
5. **Framework(title)**: The Sustainability Wheel of Fortune.
6. **Framework (Structure):** The framework has five competencies.
7. **Framework (Interpretation/ conceptualization):** The Sustainability Wheel of Fortune: A five-segmented visual representation of key knowledges and approaches required for transformative and interdisciplinary education for sustainability.
8. **Table.** The Sustainability Wheel of Fortune.

**(TOTAL FRAMEWORKS: 33) and (TOTAL DOMAINS:52)**

| **#** | **Competencies** | **Overall competencies** |
| --- | --- | --- |
| 1 | Social justice and equity: decisions need to be based on respect and regard for current and future humanity. Power differentials need to be challenged. | 373 |
| 2 | Interdisciplinary collaboration: multiple skills and perspectives are needed to tackle complex problems. | 374 |
| 3 | Limit climate change: urgent action is needed to reduce greenhouse gases (mitigation) and action is needed to prepare for climate change impacts (adaptation). | 375 |
| 4 | Ecosystem health: humanity’s future depends on protecting and maintaining healthy ecosystems and biosphere integrity. | 376 |
| 5 | Systems thinking: managing complex adaptive systems requires non-linear approaches. It pays attention to relationships and interconnections, and diversity, and resilience. | 377 |

**9. Financial support:** Open access publishing facilitated by Deakin University, as part of the Wiley - Deakin University agreement via the Council of Australian University Librarians.

**10. Conflict of interest:** The authors declare no conflict of interest

**Nineteenth: Unit of analysis.**

1. **References:** Bergstrom Katy., & Ozler, Berk. (2021). Improving the Well-Being of Adolescent Girls in Developing Countries. Policy Research Working Paper;No. 9827. © World Bank, Washington,DC. http://hdl.handle.net/10986/36478 License: [CC BY 3.0 IGO](http://creativecommons.org/licenses/by/3.0/igo).
2. **Contact author:** Bergstrom kbergstrom@worldbank.org and Berk bozler@worldbank.org.
3. **Aim**: “This paper conducts a large, narrative literature review of interventions that seek to (1) increase educational attainment, (2) delay childbearing, and/or (3) delay marriage for adolescent girls in developing countries.”
4. **Focus:** “Unfortunately, for many teenage girls in developing countries, adolescence entails a fleeting transition from childhood to adulthood, when they are suddenly expected to “behave as adults even though they are not biologically, cognitively, or emotionally ready to assume adult responsibilities”.Adolescence is also a time in which girls face a multitude of hazards ranging from school dropout, child marriage, teen pregnancy, physical and mental health problems, and gender based violence”. “Interventions that help adolescent girls reach their full potential by increasing their education, improving their skills, and delaying childbearing and marriage have the potential to create a virtuous cycle that improves health, especially child health, and women’s empowerment – ultimately leading to higher economic growth.”
   a. Human well-being.
   b. Social well-being.
   c. Economic well-being.
   d. Planetary well-being.
5. **Framework(title)**: Life-Skills Training, Mentoring, and Empowerment Programs.
6. **Framework (Structure):** Authors mentioned five competencies.
7. **Framework (Interpretation/ conceptualization):** Competencies are not defined.
8. **Table.** Life-Skills Training, Mentoring, and Empowerment Programs.

**(TOTAL FRAMEWORKS: 34) and (TOTAL DOMAINS:52)**

| **#** | **Competencies** | **Overall competencies** |
| --- | --- | --- |
| 1 | Workplace readiness. | 378 |
| 2 | Emotional regulation. | 379 |
| 3 | Interpersonal skills. | 380 |
| 4 | Attitudes regarding gender equality. | 381 |
| 5 | Leadership and entrepreneurship-skills. | 382 |

**9. Financial support:** This paper is a product of the Development Research Group, Development Economics and the Health, Nutrition and Population Global Practice. It is part of a larger effort by the World Bank to provide open access to its research and make a contribution to development policy discussions around the world. Policy

**10. Conflict of interest:** Not mentioned.

**Twentieth: Unit of analysis.**

1. **References:** Jordan, M. A. (2021). The Role of the Health Coach in a Global Pandemic. Global Advances in Health and Medicine, 10. <https://doi.org/10.1177/21649561211039456>.
2. **Contact author:** Meg A Jordan,Email: mjordan@ciis.edu.
3. **Aim**:“ Since a healthy lifestyle is fundamental for lowering risk to chronic diseases, public health initiatives to manage this and future pandemics should include strategies that assist individuals to improve health status through targeted behavior changes. This conceptual paper builds a case for certified professional health coaches as primary actors in future preventive strategies, with expanded skills in addressing social determinants of health and “next generational” cultural competencies.”
4. **Focus:** “While medical teams were perplexed about the novel SARS-CoV-2 infection, transmission and impairment of organ systems and immune function, viral infections spread worldwide. Complex intersectional issues of co-morbidities coupled with marginalized, diverse ethnic/racial populations emerged as significant risks to contracting severe COVID-19.”
   a. Human well-being.
   b. Social well-being.
   c. Economic well-being.
   d. Planetary well-being.
5. **Framework(title)**: Jordan et al´s framework.
6. **Framework (Structure):** The framework contains domains and competencies.
7. **Framework (Interpretation/ conceptualization):**
   a. Next-Generational Cultural Competence for Health Coaches: “This moral reckoning requires an overhaul of outmoded diversity trainings and stale cultural competency assessments that have stopped short of demanding deep-seated inquiries into ways most of us unconsciously engage in implicit bias, participate in white power systems, and prop up systemic structural racism. Additional training and education of health coaches in next generational cultural competency training should be developed to more effectively engage with and support individuals and families throughout underserved communities of colour, especially Black, Indigenous, and people of color (BIPOC) who may experience marginalization and exclusion from mainstream preventive health endeavours.”
   b. Coaching the Social Determinants of Health (SDoH): “While addressing SDoH is normally the purview of public health, epidemiology and social work, it would be worthwhile to expand the tools in the health coach’s toolkit to include skills in coaching SDoH. It is time to adopt a wider perspective to reverse longstanding health inequities. The coach approach provides a respectful, peer-like conversation that evokes versus admonishes, refrains from giving advice or lecturing, and leads with empathy, while building rapport and tracking progress. Expanding the coaching dialog to actively support an individual’s struggle with the harsh realities of securing employment, education, food, housing and safety.
8. Table. Jordan et al´s framework.

**(TOTAL FRAMEWORKS: 35) and (TOTAL DOMAINS:53)**

| **#** | **Domains and competencies** | **Overall competencies** |
| --- | --- | --- |
| **Domain 1: Next-Generational Cultural Competence for Health Coaches.** | | |
| Competency  1 | Assess your personal cultural effectiveness with valid tools (i.e., National Center for Cultural Competence measures growth toward self-identified competency goals). | 383 |
| Competency  2 | Examine unconscious bias and judgmental assumptions. | 384 |
| Competency  3 | Make an audit of personal privileges and subjugations. | 385 |
| Competency  4 | Become aware of historical racial/ethnic injustices in health care. | 386 |
| Competency  5 | Learn more about clients’ cultural backgrounds. | 387 |
| Competency  6 | Find out about terms: white power, structural racism, Blackness, wokeness. | 388 |
| Competency  7 | Practice inclusive language and messaging. | 389 |
| Competency  8 | Elicit with different perspectives with openness curiosity, sensitivity. | 390 |
| Competency  9 | Learn to hold difficult cross-racial dialogue. | 391 |
| Competency  10 | Develop skills in emotional self-regulation when actively listening to stories of trauma or injustice; find ways to process guilt, fragility or internalized superiority. | 392 |
| Competency  11 | Learn to center BIPOC voices from the margins as a new practice. | 393 |
| Competency  12 | Get involved in racial/ethnic justice organizations. | 394 |
| Competency  13 | Recruit and train health coaches from underrepresented groups. | 395 |
| Competency  14 | Develop skill in affirming the lived experiences of victims of racism and discrimination. | 396 |
| Competency  15 | Hear feedback nondefensively; sincerely apologize, offer to make amends Seek out friendships with those who look different than you. | 397 |
| Competency  16 | Commit to learning how to be an ally, read, listen to diverse voices. | 398 |
| Competency  17 | Engage in a lifelong process of learning and growth; practice self-compassion while doing so. | 399 |
| Competency  18 | Inspiration for these ideas is extended to Ibram X. Kendri (How to Be an Anti-Racist), Michelle Alexander (The New Jim Crow), Isabelle Wilkerson (Caste, The Origins of Our Discontent), Robin DiAngela (White Fragility). | 400 |
| **Domain 2: Coaching the Social Determinants of Health (SDoH).** | | |
| Competency  19 | Build a more complete health coaching plan that includes nonmedical SDoH factors. | 401 |
| Competency  20 | Generate a comprehensive updated list of local resources and introduce yourself. | 402 |
| Competency  21 | Facilitate in-session experimentation with role play for expressing self-advocacy. Grow adept at disseminating information with permission when required. | 403 |
| Competency  22 | Identify specific community networks that address cross-cultural support. | 404 |
| Competency  23 | Create a multi-dimensional SDoH wellness wheel to explore client’s level of satisfaction in each dimension. | 405 |
| Competency  24 | Housing: Explore tenants’ rights, address housing instability and security, discover value-oriented efforts to revalue residences. | 406 |
| Competency  25 | Food: Strengthen access to foods that support healthy eating; reduce food insecurity, advocate for local farmers markets and elimination of food deserts. | 407 |
| Competency  26 | Employment: Support efforts to rise above poverty, address fairness in workplace, explore job training, find recourse for discrimination issues. | 408 |
| Competency  27 | Education: Promote high School enrolment, retention, and graduation, explore grants for higher education; facilitate literacy training, explore support for early childhood education and development. | 409 |
| Competency  28 | Social and Community: Facilitate expansion into positive social networks and strengthen sense of belonging, explore civic participation in groups that work to reduce incarceration and discrimination. | 410 |
| Competency  29 | Neighbourhood and Built Environment: Engage with local leadership to reduce crime and violence; facilitate reduction of risk and injury, incarceration and recidivism, participate in neighbourhood watch, for crime-free residency, safe well-lighted walkways and transit; identify recreational, play settings, promote green spaces, community gardens. | 411 |
| Competency  30 | Health & Health Care: Facilitate access to quality primary care and improve health literacy. | 412 |
| Competency  31 | Support client autonomy to narrow the focus and implement small steps with accountable tracking. | 413 |
| Competency  32 | Facilitate learning about how to coordinate with public health, community agencies, and social support services as desired by client. | 414 |

**9. Financial support:** The author(s) received no financial support for the research, authorship, and/or publication of this article.

**10. Conflict of interest:** The author(s) declared no potential conflicts of interest with respect to the research, authorship, and/or publication of this article.

**Twenty-one: Unit of analysis.**

1. **References:** World Health Organization. Regional Office for Europe. (‎2020)‎. Health and well-being in the voluntary national reviews of the 2030 Agenda for Sustainable Development in the WHO European Region 2016–2020. World Health Organization. Regional Office for Europe. <https://iris.who.int/handle/10665/340007>. License: CC BY-NC-SA 3.0 IGO.
2. **Contact author:** World Health Organization Regional Office for Europe eurocontaWHO/ EURO:2020-2121-41876-5744 0ct@who.int.
3. **Aim**: “This report analyses the 60 voluntary national reviews (VNRs) submitted by 52 Member States in the WHO European Region between 2016 and 2020, examining how they incorporated and reported health and well-being issues. It identifies similarities and differences between Member States in their efforts to implement the 2030 Agenda for Sustainable Development, and how they are working towards achieving better, more equitable and sustainable health and well-being for all at all ages.
4. **Focus:** “This review highlights how the VNR process is one platform that can be used to promote and advance health and well-being issues in the WHO European Region. Working towards achieving the health and health-related SDGs requires comprehensive, context-specific, inclusive and participatory processes within Member States and for Member States to implement a set of coherent, evidence- informed policies that address health, well-being and all their determinants throughout the life-course and across all sectors of government and society.”
   a. Human well-being.
   b. Social well-being.
   c. Economic well-being.
   d. Planetary well-being.
5. **Framework(title)**: World Health Organization´s framework.
6. **Framework (Structure):** A sentence with competencies.
7. **Framework (Interpretation/ conceptualization)** Data science and analytic skills are not conceptualized.
8. **Table** World Health Organization´s framework

**(TOTAL FRAMEWORKS: 36) and (TOTAL DOMAINS:53)**

| **#** | **Competency** | **Overall competencies** |
| --- | --- | --- |
| 1 | Data science and analytic skills. | 415 |

9. **Financial support:** not provided
10. **Conflict of interest:** not provided

**Twenty-second: Unit of analysis.**

1. **References:** Commonwealth of Australia . (2017). National Strategic Framework for Aboriginal and Torres Strait Islander Peoples’ Mental Health and Social and Emotional Wellbeing (2017-2023). Department of the Prime Minister and Cabinet, the Australian Government.<https://www.niaa.gov.au/sites/default/files/documents/publications/mhsewb-framework_0.pdf>.
2. **Contact author:** Australian government, not provided the email.
3. **Aim**: “This renewed Framework is intended to guide and inform Aboriginal and Torres Strait Islander mental health and wellbeing reforms. It aims to respond to the high incidence of social and emotional wellbeing problems and mental ill-health, by providing a Framework for action. Further statistical information about Aboriginal and Torres Strait Islander mental health and related areas and service use is at Appendix 1. It also aims to contribute to the vision of the National Aboriginal and Torres Strait Islander Health Plan 2012-2023, which includes achieving the Council of Australian Governments’ Closing the Gap target for Aboriginal and Torres Strait Islander and non Indigenous life expectancy equality (as a measure of health equality) by 2031. Finally, the Australian Government has committed to continue to seek advice from Aboriginal and Torres Strait Islander mental health and related areas leaders and stakeholders to shape reform at the national level.4 This Framework is also intended to support this ongoing process.”
4. **Focus:** “For Aboriginal and Torres Strait Islander people, families and communities to achieve and sustain the highest attainable standard of social and emotional wellbeing and mental health supported by mental health and related services that are effective, high quality, clinically and culturally appropriate, and affordable”
   a. Human well-being.
   b. Social well-being.
5. **Framework(title)**: National Strategic Framework for Aboriginal and Torres Strait Islander Peoples’ Mental Health and Social and Emotional Wellbeing.
6. **Framework (Structure):** The Mental Health and Social and Emotional Wellbeing Framework in Action contains five actions areas: Strengthen the Foundations, Promote Wellness, Build Capacity and Resilience in People and Groups at Risk, Provide Care for People who are Mildly or Moderately Ill, and Care for People Living with a Severe Mental Illness. The framework is supported by cultural competencies.
7. **Framework (Interpretation/ conceptualization):** Cultural competencies are a skill set that can be gained by experience working with Aboriginal and Torres Strait Islander people and by training modules.
8. **Table** National Strategic Framework for Aboriginal and Torres Strait Islander Peoples’ Mental Health and Social and Emotional Wellbeing.

**(TOTAL FRAMEWORKS: 37) and (TOTAL DOMAINS:54)**

| # |  | **Overall competencies** |
| --- | --- | --- |
| **Domain: Cultural competencies.** | | |
| 1 | Cultural Awareness: Understanding the role of cultural difference and diversity. For non-Indigenous staff this means the capacity for self-reflection as to how the Western dominant culture impacts on both themselves and on Aboriginal and Torres Strait Islander people, and can impact the service setting they operate in. | 416 |
| 2 | Cultural Respect: Valuing Aboriginal and Torres Strait Islander people and cultures. This includes a commitment to self-determination and building respectful partnerships. | 417 |
| 3 | Cultural Responsiveness: Having the ability and skills to assist people of a different culture other than your own. | 418 |

**9. Financial support:** not provided

**10. Conflict of interest:** not provided

**Twenty-third: Unit of analysis.**

1. **References:** World Health Organization. Regional Office for Europe. (‎2013)‎. Health 2020: a European policy framework supporting action across government and society for health and well-being (‎short version)‎. World Health Organization. Regional Office for Europe. <https://iris.who.int/handle/10665/131300>.
2. **Contact author**: World Health Organization, the email is not provided.
3. **Aim**: “Health 2020 focuses on improving health for all and reducing health inequalities, through improved leadership and governance for health.”
4. **Focus: “**improving health for all and reducing health inequalities.” “The framework crystallizes what we have learned about the role of health in recent years. maximizing health through all stages of life is a fundamental right for all and not a privilege for a few. good health is an asset and a source of economic and social stability. It is key to reducing poverty and both contributes to and benefits from sustainable development. most important, good health can no longer be seen an outcome of one sector alone: sustainable and equitable improvements in health are the product of effective policy across all parts of government and collaborative efforts across all parts of society.” “A whole-of-society approach goes beyond institutions: it influences and mobilizes local and global culture and media, rural and urban communities and all relevant policy sectors, such as the education system, the transport sector, the environment and even urban design, as demonstrated in the case of obesity and the global food system.”
   a. Human well-being.
   b. Social well-being.
   c. Economic well-being.
   d. Planetary well-being.
5. **Framework(title)**: Improving leadership and participatory governance for health
6. **Framework (Structure):** The document provides five competencies for leadership.
7. **Framework (Interpretation/ conceptualization): “**Providing training professionals and political actors that participate in healthy cities networks take part in training that supports them in developing leadership and other skills to implement urban health policies.”
8. **Table** Improving leadership and participatory governance for health

**(TOTAL FRAMEWORKS: 38) and (TOTAL DOMAINS:54)**

| **#** | **Competencies** | **Overall competencies** |
| --- | --- | --- |
| 1 | Managing conflicts of interest. | 419 |
| 2 | Finding new ways of tackling intractable complex problems. | 420 |
| 3 | Supporting patient empowerment and self-care. | 421 |
| 4 | Working across sectors and leadership capacity. | 422 |
| 5 | Knowledge from the social, behavioural and policy sciences is proving increasingly important, including social marketing, behavioural economics and neuroscience. | 423 |

**9. Financial support:** not provided

**10. Conflict of interest:** not provided

**TOTAL**

- Frameworks ( 38: TOTAL)
- Domains (54:TOTAL)
- Competencies (423: TOTAL)
