# Supplementary material for: Towards a competency framework for wellbeing economy in public health: a scoping review
Source: Public Health Rev. 2026 Aug 5;47:1609947. doi: 10.3389/phrs.2026.1609947 (PMC13500908; doi:10.3389/phrs.2026.1609947)
Supplement: Supplementary file 6 [file Supplementaryfile6.docx]

´

**Supplementary material 6.** Domains and their interpretation.

**Table Domains**

| **#** | **Domains** | **Interpretation** | **Unit of analysis.** |
| --- | --- | --- | --- |
| 1 | Socioemotional and Psychosocial Well-being | Socioemotional well-being is a state characterized by positive emotions, positive relationships, and the ability to manage challenging emotions and situations. The components of socioemotional well-being for young children include | 2 |
| 2 | Digital literacy | To establish trust based on people’s ability to verify the credibility of online content and information sources | 3 |
| 3 | Information and data literacy | Not provided | 5 |
| 4 | Communication & collaboration | Not provided | 5 |
| 5 | Digital content creation | Not provided | 5 |
| 6 | Safety | The last two areas are “transversal” as safety and problem solving apply to any type of activity carried out using digital technology | 5 |
| 7 | Problem solving | : The last two areas are “transversal” as safety and problem solving apply to any type of activity carried out using digital technology | 5 |
| 8 | Data, information, and digital documents | Not provided | 5 |
| 9 | Communication and sharing | Not provided | 5 |
| 10 | Safety | Not provided | 5 |
| 11 | Online services | Not provided | 5 |
| 12 | Digital transformation: | Not provided | 5 |
| 13 | Strategy and business development | Not provided | 5 |
| 14 | Projects & further development | Not provided | 5 |
| 15 | Data & security | Not provided | 5 |
| 16 | Governance and co-operation | Not provided | 5 |
| 17 | Health policy research | Not provided. | 7 |
| 18 | Policy analysis | Not provided. | 7 |
| 19 | Educational competencies | Not provided. | 7 |
| 20 | Decision-making | Not provided. | 7 |
| 21 | Multidisciplinary work | Not provided. | 7 |
| 22 | Community alignment | align with community needs, strengths, and history – adapt to context: The community initiatives included were sensitive to the context in which they operated. During initiation and planning, the collection of subjective and objective data enabled a contextual understanding of community need. | 8 |
| 23 | Community ownership | encourage bottom-up energy to build community ownership: The first step to generate community ownership, was to keep the community voice and vision as the anchor point for all planning and leadership. | 8 |
| 24 | Engaged community | Include community in strategy and action to build trust: Five initiatives brought the community together to discuss the future, which was key to their planning and visioning, and may have played a role in engaging community members in leadership or working group positions. | 8 |
| 25 | Shared purpose | Establish based on a collective understanding of wellbeing: A shared vision that reflected the community voice and aligned with the local context was an important factor in the organisation of initiatives | 8 |
| 26 | Collaborative action | form and maintain relationships and partnerships: The selected initiatives had a locally based, collaborative leadership team | 8 |
| 27 | Transparent communication | Openly communicate with community and partners, including promotion activities: Active communication between the initiative and the broader community was used to engage the community for initial discussion; to involve members as leaders, volunteers, or champions to advertise the purpose and vision of the initiative; to publicise the plan; to advertise sponsored or organised activities; and to list key community contacts for support or involvement. | 8 |
| 28 | Continuous learning | Monitor and evaluate activities: Each initiative adapted over time as they learned how to operate and be effective | 8 |
| 29 | Resource management | secure and use resources flexibly: Several of the initiatives were described from the perspective of the funders, making it challenging to assess the financial resource dimension. Some initiatives were established only as funding was secured; others secured funding as they went along. Funding was often discussed, including receipt or application for funding and how relationships with funders were managed. | 8 |
| 30 | Complexity and system thinking | Many participants stressed the importance of a solid foundation of factual knowledge that takes the complexity of the topic into account and at the same time introduces a perspective of systems thinking. | 11 |
| 31 | Ethical dimension | An ethical discussion of issues such as gender-based discrimination, racism, sexism, capitalism, and modern medical treatments and their impact on the environment should be included. “Social justice” in terms of a “global justice perspective” and the different regional impacts of environmental change also need to be discussed. | 11 |
| 32 | Responsibility of health professionals | A reflection of one’s own position in society as well as options for action or even agency, including the role of a change agent as a healthcare professional, should be part of planetary health education (PHE). | 11 |
| 33 | Transformative competencies including practical skills | By acquiring knowledge about possible actions and reflecting about their own options for action | 11 |
| 34 | Space for reflection and resilience building | Learners need enough space for guided and unguided reflection inside and outside the classroom | 11 |
| 35 | Health literacy | Health literacy encompasses the knowledge, motivation and skills required to access, comprehend, assess and apply information to make informed decisions regarding healthcare, disease prevention and the enhancement of overall well-being over the life course | 13 |
| 36 | Socio-emotional and communication skills: | These skills are critical to building a resilient green and digital transition because these are uniquely human skills and, therefore, not easily automatable even with advances in Artificial intelligence in the near future. Moreover, the collective effort needed to promote the green transition rests on the capacity of communities to negotiate conflict and pool collective expertise to solve complex systemic problems. | 13 |
| 37 | Raising awareness about the role of metacognitive skills | metacognitive skills allow individuals to recognise the role of heuristics and biases in shaping decision making and make more informed use of knowledge and information. Metacognitive skills, in fact, empower individuals by helping them characterise knowledge and knowledge generation processes and can help individuals effectively work alongside others – whether humans or machines. They help individuals and communities solve complex problems and develop reasonable risk mitigation strategies both at individual and societal levels. | 13 |
| 38 | Government (All Levels) | This domain targets structural actions that can be taken by governments through the governing systems of public and private sectors. It refers to macro-economic, public, and social policies as well as underlying power structures | 14 |
| 39 | Organizational & Political Culture: | This domain targets those processes that affect the delivery and sustainability of government, organizational, and community actions. It involves consideration of organizational culture and power dynamics | 14 |
| 40 | Socioeconomic & Political Context | This domain targets social and political actions. It encompasses changes to the political and community landscape that, together, shape the availability of resources, opportunities for poverty reduction, possibilities for growth of the middle-class, and improvements in the distribution of power at the societal level | 14 |
| 41 | Social & Cultural Circumstances | This domain is about political, community, organizational, and individual actions that shape or recognize social and cultural contexts, hierarchies of power, and people’s social backgrounds and identities (e.g., immigration status, gender, sexual orientation, race/ethnicity) that accumulate to impact their financial circumstances | 14 |
| 42 | Life Circumstances | This domain targets political, community, organizational, and individual actions that impact people’s complex life circumstances, multiple roles, and power relationships (e.g., individual agency and power within a household) that come together – positively or negatively – to shape their financial situation. | 14 |
| 43 | Soft skills | Not provided. | 15 |
| 44 | Professional skills | Not provided. | 15 |
| 45 | Subject matter knowledge | Not provided. | 15 |
| 46 | Determinants of disease and One Health: | Multiple factors determine the health of individuals and communities, including location and environment, genetics, income, educational access and other social factors. One Health provides an important overarching framework to work within. While not all global health issues, such as vaccine preventable diseases like measles are seen as direct One Health issues, most health security threats are. One Health aims to reduce the overall threats to global health and its security, through reducing the risk of emergence at its source. | 17 |
| 47 | Adaptive and function- based frameworks | Not provided. | 17 |
| 48 | Evidence-based decision-making. | Evidence from research outside the assessment tools, however, must also be considered, for instance operational and implementation research can help decision- making to maximise strategic value of services and systems. | 17 |
| 49 | Engagement at multiple levels | Global health systems are complex, where one capacity has direct and indirect impacts on the effectiveness of another, and these may be influenced at local, national, regional and international levels | 17 |
| 50 | Communication and misinformation | Prioritisation involves consideration of social context, including engagement of communities, local media, social cohesion and misinformation management. Misinformation, particularly but not only on social media… Transparency in knowledge and information sharing on platforms that are easily accessible to the public may help build trust. | 17 |
| 51 | Diversity at the decision- making table | Prioritisation itself needs a good governance model. To create ‘stronger and more inclusive health emergency preparedness, response and resilience architecture’, prioritisation needs to operate within marginal economies, be accountable, transparent, participatory, equitable, consistent with the rule of law and evidence based. | 17 |
| 51 | Regional and collaborative entities | Frequently missing in prioritisation discussions is the importance of regional entities. Individual states can benefit from collaborative action with international and NGO groups, including private sector companies, to optimise collective efforts for information sharing, planning, surveillance, financial support, access to equipment and pharmaceuticals, technical training and capacity building. | 17 |
| 52 | Next-Generational Cultural Competence for Health Coaches | This moral reckoning requires an overhaul of outmoded diversity trainings and stale cultural competency assessments that have stopped short of demanding deep-seated inquiries into ways most of us unconsciously engage in implicit bias, participate in white power systems, and prop up systemic structural racism. Additional training and education of health coaches in next generational cultural competency training should be developed to more effectively engage with and support individuals and families throughout underserved communities of colour, especially Black, Indigenous, and people of color (BIPOC) who may experience marginalization and exclusion from mainstream preventive health endeavours | 20 |
| 53 | Coaching the Social Determinants of Health (SDoH) | While addressing SDoH is normally the purview of public health, epidemiology and social work, it would be worthwhile to expand the tools in the health coach’s toolkit to include skills in coaching SDoH. It is time to adopt a wider perspective to reverse longstanding health inequities.52 The coach approach provides a respectful, peer-like conversation that evokes versus admonishes, refrains from giving advice or lecturing, and leads with empathy, while building rapport and tracking progress. Expanding the coaching dialog to actively support an individual’s struggle with the harsh realities of securing employment, education, food, housing and safety. | 20 |
| 54 | Cultural competencies | skill set that can be gained by experience working with Aboriginal and Torres Strait Islander people and by training modules. | 22 |
