# Supplementary material for: Towards a competency framework for wellbeing economy in public health: a scoping review
Source: Public Health Rev. 2026 Aug 5;47:1609947. doi: 10.3389/phrs.2026.1609947 (PMC13500908; doi:10.3389/phrs.2026.1609947)
Supplement: Supplementary file 7 [file Supplementaryfile7.docx]

**Supplementary material 7.** Selection of sources of evidence

**Table 1 .** Literature that was excluded after full-text ( First mechanism: Databases )

| # | Citation | Reason for exclusion |
| --- | --- | --- |
| 1 | Breheny, K., Salway, R., House, D., Walker, R., Emm-Collison, L., Sansum, K., Williams, J. G., de Vocht, F., Jago, R., & Hollingworth, W. (2024). Quality of life, capability well-being, financial strain and physical activity in the short- and medium-term COVID-19 post-lockdown phases in the UK: a repeated cross-sectional study. *Public health research (Southampton, England)*, 1–19. Advance online publication. https://doi.org/10.3310/LYJG6305 | No framework with competencies |
| 2 | Camp, E. F., Braverman, I., Wilkinson, G., & Voolstra, C. R. (2024). Coral reef protection is fundamental to human rights. *Global change biology*, *30*(9), e17512. https://doi.org/10.1111/gcb.17512 | Study design: No methodology |
| 3 | Gawlik, K. S., Teall, A. M., Zeno, R., Newtz, C., Conrad, K., Kolcun, K., Bobek, H., Deerhake, A., Sullivan, K., Rengers, B., & O'Hara, S. (2024). Integrating wellness into curricula using the ten dimensions of wellness as a framework. *Journal of professional nursing : official journal of the American Association of Colleges of Nursing*, *50*, 73–82. https://doi.org/10.1016/j.profnurs.2023.11.006 | Study design: No methodology |
| 4 | Stein, P. J. S., Stein, M. A., Groce, N., Kett, M., Akyeampong, E. K., Alford, W. P., Chakraborty, J., Daniels-Mayes, S., Eriksen, S. H., Fracht, A., Gallegos, L., Grech, S., Gurung, P., Hans, A., Harpur, P., Jodoin, S., Lord, J. E., Macanawai, S. S., McClain-Nhlapo, C. V., Mezmur, B. D., … Van Susteren, L. (2024). Advancing disability-inclusive climate research and action, climate justice, and climate-resilient development. *The Lancet. Planetary health*, *8*(4), e242–e255. https://doi.org/10.1016/S2542-5196(24)00024-X | Study design: No methodology |
| 5 | Giri, M., & Hilado, A. (2024). Health Care and Humanitarian Considerations for Refugee and Immigrant Children. *Pediatric annals*, *53*(5), e159–e160. <https://doi.org/10.3928/19382359-20240409-03> | Study design: No methodology |
| 6 | Oster, C., & Bogomolova, S. (2024). Potential lateral and upstream consequences in the development and implementation of social prescribing in Australia. *Australian and New Zealand journal of public health*, *48*(1), 100121. https://doi.org/10.1016/j.anzjph.2023.100121 | Study design: No methodology |
| 7 | Ghosh, A. K., Azan, A., Basu, G., Bernstein, J., Gillespie, E., Gordon, L. B., Krishnamurthy, S., LeFrancois, D., Marcus, E. N., Tejani, M., Townley, T., Rimler, E., Whelan, H., & SGIM Environmental Health Interest Group, Medical Education Committee (2024). Building Climate Change into Medical Education: A Society of General Internal Medicine Position Statement. *Journal of general internal medicine*, *39*(13), 2581–2589. https://doi.org/10.1007/s11606-024-08690-1 | No framework with competencies |
| 8 | Fillol, A., Wallerich, L., Larose, M. P., Ferron, C., Rivadeneyra-Sicilia, A., Vandentorren, S., Brandler-Weinreb, J., & Cambon, L. (2024). The Influence of Educational Determinants on Children's Health: A Scoping Review of Reviews. *Public health reviews*, *45*, 1606372. https://doi.org/10.3389/phrs.2024.1606372 | No framework with competencies |
| 9 | Dupuy, C., Locquet, C., Brard, C., Dommergues, L., Faure, E., Gache, K., Lancelot, R., Mailles, A., Marchand, J., Payne, A., Touratier, A., Valognes, A., & Carles, S. (2024). The French National Animal Health Surveillance Platform: an innovative, cross-sector collaboration to improve surveillance system efficiency in France and a tangible example of the One Health approach. Frontiers in Veterinary Science, 11. https://doi.org/10.3389/fvets.2024.1249925 | Study design: No methodology |
| 10 | Riojas-Rodríguez, H., Rivera-Dommarco, J. A. ., Torres-Pereda, P., Unar-Munguía, M., Cortez-Lugo, M., & Zúñiga-Bello, P. (2024). Salud planetaria, una nueva salud pública global. *Salud Pública De México*, *66*(6 (nov-dec), 859-867. https://doi.org/10.21149/15410 | Language- Spanish- |
| 11 | Jolles, M. P., Fort, M. P., & Glasgow, R. E. (2024). Aligning the planning, development, and implementation of complex interventions to local contexts with an equity focus: application of the PRISM/RE-AIM Framework. International Journal for Equity in Health, 23(1), 41. https://doi.org/10.1186/s12939-024-02130-6 | Study design: No methodology |
| 12 | Desvars-Larrive, A., Burger, P., Khol, J. L., Posautz, A., Schernhammer, E., Kutalek, R., Puspitarani, G. A., Schlippe Justicia, L., Springer, D. N., Ernst, D., Sohm, C., Pontel de Almeida, A., Schobesberger, H., Knauf, S., & Walzer, C. (2024). Launching Austria’s One Health network: paving the way for transdisciplinary collaborations. One Health Outlook, 6(1), 23. <https://doi.org/10.1186/s42522-024-00116-6> | No framework with competencies |
| 13 | Rahman, R., Reid, C., Kloer, P., Henchie, A., Thomas, A., & Zwiggelaar, R. (2024). A systematic review of literature examining the application of a social model of health and wellbeing. European Journal of Public Health, 34(3), 467–472. https://doi.org/10.1093/eurpub/ckae008 | Reviews that must be registered on a platform |
| 14 | Saleem, S. M., & Jan, S. S. (2023). Navigating the infodemic: strategies and policies for promoting health literacy and effective communication. Frontiers in Public Health, 11, 1324330. https://doi.org/10.3389/fpubh.2023.1324330 | Study design: No methodology |
| 15 | Biryukov, V., Gozhenko, A., Gozhenko, O., Muszkieta, R., Hagner-Derengowska, M., Smoleńska, O., Michalska, A., & Zukow, W. (2024). Exploring the boundaries of medical anthropology in understanding human health. *Journal of Physical Education and Sport*, *24*, Article 2. https://doi.org/10.7752/jpes.2024.02037 | Study design: No methodology |
| 16 | Katz, S., Matairakula, U., Cinavilakeba, J., Dradra, T., Carter, R., Tikoibua, T., & Mitchell, B. G. (n.d.). Cultivating Wellbeing: Traditional Wisdom and Sustainability in Fiji’s Green Schools. Proceedings of the Nutrition Society, 83(OCE1). https://doi.org/10.1017/S0029665124000259 | Study design: No methodology |
| 17 | Mabry, R. M., Doctor, H. V., Khair, M. N. N., Abdelgalil, M., & Rashidian, A. (2024). Integrating health across the Sustainable Development Goals in the Eastern Mediterranean Region: Assessment of Voluntary National Reviews from 18 countries. *PLOS global public health*, *4*(7), e0003451. https://doi.org/10.1371/journal.pgph.0003451 | No framework with competencies |
| 18 | Wang, K., Zhang, X., & Zhou, Q. (2024). The influence of freshwater blue spaces on human health and well-being:A systematic review based on assessment method. *Environmental research*, *263*(Pt 3), 120242. https://doi.org/10.1016/j.envres.2024.120242 | No framework with competencies |
| 19 | Marchant, E. (2024). Re-energizing health literacy in Wales: a testbed for health, education and prosperity for all. Health Promotion International, 39(3). https://doi.org/10.1093/heapro/daae055 | Study design: No methodology |
| 20 | Hoy-Gerlach J, Townsend L. Reimagining Healthcare: Human-Animal Bond Support as a Primary, Secondary, and Tertiary Public Health Intervention. Int J Environ Res Public Health. 2023 Mar 27;20(7):5272. doi: 10.3390/ijerph20075272. PMID: 37047888; PMCID: PMC10094350. | Study design: No methodology |
| 21 | Santinha, G., Fernandes, A., Oliveira, R., & Rocha, N. P. (2023). Designing a Health Strategy at Local Level: A Conceptual Framework for Local Governments. International Journal of Environmental Research and Public Health, 20(13). https://doi.org/10.3390/ijerph20136250 | Study design: No methodology |
| 22 | Friel, S., Collin, J., Daube, M., Depoux, A., Freudenberg, N., Gilmore, A. B., Johns, P., Laar, A., Marten, R., McKee, M., & Mialon, M. (2023). Commercial determinants of health: future directions. Lancet (London, England), 401(10383), 1229–1240. <https://doi.org/10.1016/S0140-6736(23)00011-9> | Study design: No methodology |
| 23 | Hjelmskog, A., Toney, J., Scott, M., Crawford, J., & Meier, P. (2023). Using the Doughnut Economics framework to structure whole-system thinking in socioecological wellbeing with multidisciplinary stakeholders: an applied case study in Glasgow, Scotland. *Lancet (London, England)*, *402 Suppl 1*, S15. <https://doi.org/10.1016/S0140-6736(23)02057-3> | Study design: No methodology |
| 24 | Raymond, K., Haddock, R., Nathan, S., Harrison, R., & Meyer, L. (2023). Value-based public health: Moving beyond value-based health care to support a wellbeing economy. *Health promotion journal of Australia : official journal of Australian Association of Health Promotion Professionals*, *34*(3), 675–680. https://doi.org/10.1002/hpja.736 | Study design: No methodology |
| 25 | Yose, P., Thondhlana, G., & Fraser, G. (2023). Conceptualizing the socio-cultural impacts of marine plastic pollution on human well-being - A perspective. *Marine pollution bulletin*, *194*(Pt A), 115285. https://doi.org/10.1016/j.marpolbul.2023.115285 | Study design: No methodology |
| 26 | Urban mental health and early interventions: From interdisciplinary framework to transdisciplinary collaborations | Study design: No methodology |
| 27 | Stefanon, B. M., Tsetso, K., Tanche, K., & Morton Ninomiya, M. E. (2023). Effective health and wellness systems for rural and remote Indigenous communities: a rapid review. *International journal of circumpolar health*, *82*(1), 2215553. https://doi.org/10.1080/22423982.2023.2215553 | Reviews that must be registered on a platform |
| 28 | Heydari, A., Partovi, P., Zarezadeh, Y., & Yari, A. (2023). Exploring medical students' perceptions and understanding of the health impacts of climate change: a qualitative content analysis. *BMC medical education*, *23*(1), 774. https://doi.org/10.1186/s12909-023-04769-1 | No framework with competencies |
| 29 | Lauzon, C., Stevenson, A., Peel, K., & Brinsdon, S. (2023). A “bottom up” Health in All Policies program: Supporting local government wellbeing approaches. Health Promotion Journal of Australia : Official Journal of Australian Association of Health Promotion Professionals, 34(3), 660–666. https://doi.org/10.1002/hpja.712 | Study design: No methodology |
| 30 | Basso, F., & Krpan, D. (2023). The WISER framework of behavioural change interventions for mindful human flourishing. The Lancet. Planetary Health, 7(2), e106–e108. https://doi.org/10.1016/S2542-5196(22)00336-9 | Study design: No methodology |
| 31 | Baum, F., McGreevy, M. P., MacDougall, C. M., & Henley, M. (2023). Energy as a Social and Commercial Determinant of Health: A Qualitative Study of Australian Policy. International Journal of Health Policy and Management, 12, 7193. <https://doi.org/10.34172/ijhpm.2022.7193> | No framework with competencies |
| 32 | Deivanayagam, T. A., Osborne, R. E., & Pai, M. (2023). Breaking free from tunnel vision for climate change and health. PLOS Global Public Health, 3(3). https://doi.org/10.1371/journal.pgph.0001684 | Study design: No methodology |
| 33 | Kemp, A. H., & Fisher, Z. (2022). Wellbeing, Whole Health and Societal Transformation: Theoretical Insights and Practical Applications. Global Advances in Health and Medicine, 11. https://doi.org/10.1177/21649561211073077 | Study design: No methodology |
| 34 | Karmakar, S. (2022). Artificial Intelligence: the future of medicine, or an overhyped and dangerous idea? Irish Journal of Medical Science, 191(5), 1991–1994. <https://doi.org/10.1007/s11845-021-02853-3> | Study design: No methodology |
| 35 | Chyke A. Doubeni, David Nelson, Elizabeth Gross Cohn, Electra Paskett, Seleshi Ayalew Asfaw, Mehek Sumar, Syed M. Ahmed, Rhonda McClinton-Brown, Mark L. Wieland, Anita Kinney, Sergio Aguilar-Gaxiola, Lisa G. Rosas, & Cecilia M. Patino. (2022). Community engagement education in academic health centers, colleges, and universities. Journal of Clinical and Translational Science, 6. https://doi.org/10.1017/cts.2022.424 | Study design: No methodology |
| 36 | Liina Mansukoski, Alexandra Albert, Yassaman Vafai, Chris Cartwright, Aamnah Rahman, Jessica Sheringham, Bridget Lockyer, Tiffany C. Yang, Philip Garnett, & Maria Bryant. (2022). Development of Public Health Core Outcome Sets for Systems-Wide Promotion of Early Life Health and Wellbeing. International Journal of Environmental Research and Public Health, 19(7947), 7947. https://doi.org/10.3390/ijerph19137947 | No framework with competencies |
| 37 | Testing the Public Health 12 framework using a community-based systems thinking obesity prevention case study | No access to full text. |
| 38 | Donaldson, C., & Mitton, C. (n.d.). Health economics and emergence from COVID-19 lockdown: the great big marginal analysis. Health Economics, Policy and Law, 17(2), 227–231. https://doi.org/10.1017/S1744133120000304 | Study design: No methodology |
| 39 | Brand-Correa, L., Brook, A., Büchs, M., Meier, P., Naik, Y., & O’Neill, D. W. (2022). Economics for people and planet-moving beyond the neoclassical paradigm. The Lancet. Planetary Health, 6(4), e371–e379. https://doi.org/10.1016/S2542-5196(22)00063-8 | Study design: No methodology |
| 40 | Leconstant, C., & Spitz, E. (2022). Integrative Model of Human-Animal Interactions: A One Health-One Welfare Systemic Approach to Studying HAI. Frontiers in Veterinary Science, 9, 656833. https://doi.org/10.3389/fvets.2022.656833 | Study design: No methodology - |
| 41 | Röhr, S., Rodriguez, F. S., Siemensmeyer, R., Müller, F., Romero-Ortuno, R., & Riedel-Heller, S. G. (2022). How can urban environments support dementia risk reduction? A qualitative study. International Journal of Geriatric Psychiatry, 37(1). https://doi.org/10.1002/gps.5626 | No framework with competencies |
| 42 | Littleton, C., & Reader, C. (2022). To what extent do Australian child and youth health, and education wellbeing policies, address the social determinants of health and health equity?: a policy analysis study. *BMC public health*, *22*(1), 2290. https://doi.org/10.1186/s12889-022-14784-4 | No framework with competencies |
| 43 | Jagals, P., Ebi, K., & Tchounwou, P. B. (2021). Core Competencies for Health Workers to Deal with Climate and Environmental Change. International Journal of Environmental Research and Public Health, 18(8). https://doi.org/10.3390/ijerph18083849 | Study design: No methodology - |
| 44 | Pathak, N., & McKinney, A. (2021). Planetary Health, Climate Change, and Lifestyle Medicine: Threats and Opportunities. American Journal of Lifestyle Medicine, 15(5), 541–552. https://doi.org/10.1177/15598276211008127 | Study design: No methodology - |
| 45 | Sangtien Youthao, & Somsak Amornsiriphong. (2021). One Health information management: health system reforms to support social well-being in Thailand. International Journal of One Health, 7(1), 96–103. https://doi.org/10.14202/IJOH.2021.96-103 | No framework with competencies |
| 46 | Wright, C. Y., Moore, C. E., Chersich, M., Hester, R., Nayna Schwerdtle, P., Mbayo, G. K., Akong, C. N., & Butler, C. D. (2021). A Transdisciplinary Approach to Address Climate Change Adaptation for Human Health and Well-Being in Africa. International Journal of Environmental Research and Public Health, 18(8). https://doi.org/10.3390/ijerph18084258 | Study design: No methodology - |
| 47 | MacNeill, A. J., McGain, F., & Sherman, J. D. (2021). Planetary health care: a framework for sustainable health systems. The Lancet. Planetary Health, 5(2), e66–e68. <https://doi.org/10.1016/S2542-5196(21)00005-X> | Study design: No methodology - |
| 48 | Consolazio, D., Terraneo, M., & Tognetti, M. (2021). Social cohesion, psycho-physical well-being and self-efficacy of school-aged children in Lombardy: Results from HBSC study. Health & Social Care in the Community, 29(6), 1729–1737. https://doi.org/10.1111/hsc.13278 | No access to full text |
| 49 | Shane Clifton, Nicola Fortune, Gwynnyth Llewellyn, Roger J. Stancliffe, & Paul Williamson. (2020). Lived Expertise and the Development of a Framework for Tracking the Social Determinants, Health, and Wellbeing of Australians with Disability. Scandinavian Journal of Disability Research, 22(1), 137–146. https://doi.org/10.16993/sjdr.688 | No framework with competencies |
| 50 | Seeing Yourself in Your Room: The Relationship between Undergraduate Flourishing and Identification with Student Housing | No access to full text |
| 51 | Benis, A., Tamburis, O., Chronaki, C., & Moen, A. (2021). One Digital Health: A Unified Framework for Future Health Ecosystems. *Journal of medical Internet research*, *23*(2), e22189. https://doi.org/10.2196/22189 | Study design: No methodology - |
| 52 | Lainé, N., & Morand, S. (2020). Linking humans, their animals, and the environment again: a decolonized and more-than-human approach to "One Health". Relier à nouveau les humains, leurs animaux et l’environnement : une approche décolonisée et plus qu’humaine de « One Health ». *Parasite (Paris, France)*, *27*, 55. https://doi.org/10.1051/parasite/2020055 | Study design: No methodology - |
| 53 | Kerman, N., Lem, M., Witte, M., Kim, C., & Rhoades, H. (2020). A Multilevel Intervention Framework for Supporting People Experiencing Homelessness with Pets. *Animals : an open access journal from MDPI*, *10*(10), 1869. https://doi.org/10.3390/ani10101869 | Study design: No methodology - |
| 54 | Harris, P., Riley, E., Dawson, A., Friel, S., & Lawson, K. (2020). "Stop talking around projects and talk about solutions": Positioning health within infrastructure policy to achieve the sustainable development goals. *Health policy (Amsterdam, Netherlands)*, *124*(6), 591–598. <https://doi.org/10.1016/j.healthpol.2018.11.013> | No framework with competencies |
| 55 | A proposed framework based on literature review of online contextual mental health services to enhance wellbeing and address psychopathology during COVID-19**.** | No framework with competencies |
| 56 | Watts, P., Rance, S., McGowan, V., Brown, H., Bambra, C., Findlay, G., & Harden, A. (2020). The long-term health and wellbeing impacts of Healthy New Towns (HNTs): protocol for a baseline and feasibility study of HNT demonstrator sites in England. *Pilot and feasibility studies*, *6*, 4. https://doi.org/10.1186/s40814-020-0550-2 | No study design- Protocol- |
| 57 | Hensher, M., Canny, B., Zimitat, C., Campbell, J., & Palmer, A. (2020). Health care, overconsumption and uneconomic growth: A conceptual framework. *Social science & medicine (1982)*, *266*, 113420. https://doi.org/10.1016/j.socscimed.2020.113420 | No framework with competencies |
| 58 | Capolongo, S., Rebecchi, A., Napier, D., Ricciardi, G., Signorelli, C., Serra, F., Dotta, F., Pella, R., & Lenzi, A. (2020). New competences to manage Urban Health: Health City Manager core curriculum. European Journal of Public Health, 30(Supplement_5). https://doi.org/10.1093/eurpub/ckaa165.449 | Study design: No methodology - |
| 59 | Lemery, J., Balbus, J., Sorensen, C., Rublee, C., Dresser, C., Balsari, S., & Calvello Hynes, E. (2020). Training Clinical And Public Health Leaders In Climate And Health. *Health affairs (Project Hope)*, *39*(12), 2189–2196. https://doi.org/10.1377/hlthaff.2020.01186 | Study design: No methodology - |
| 60 | Billiot, S., & Mitchell, F. M. (2019). Conceptual interdisciplinary model of exposure to environmental changes to address indigenous health and well-being. *Public health*, *176*, 142–148. https://doi.org/10.1016/j.puhe.2018.08.011 | No framework with competencies |
| 61 | Tomalin, E., Sadgrove, J., & Summers, R. (2019). Health, faith and therapeutic landscapes: Places of worship as Black, Asian and Minority Ethnic (BAME) public health settings in the United Kingdom. *Social science & medicine (1982)*, *230*, 57–65. https://doi.org/10.1016/j.socscimed.2019.03.006 | No framework with competencies |
| 62 | Higgins, S. L., Thomas, F., Goldsmith, B., Brooks, S. J., Hassall, C., Harlow, J., Stone, D., Völker, S., & White, P. (2019). Urban freshwaters, biodiversity, and human health and well-being: Setting an interdisciplinary research agenda. Wiley Interdisciplinary Reviews: Water, 6(2). https://doi.org/10.1002/wat2.1339 | Study design: No methodology - |
| 63 | Serrate, P. C.-F. (2019). Universal Health in Cuba: Healthy Public Policy in All Sectors. MEDICC Review, 21(4), 74–77. | Study design: No methodology - |
| 64 | Pongsiri, M. J., Bickersteth, S., Colón, C., DeFries, R., Dhaliwal, M., Georgeson, L., Haines, A., Linou, N., Murray, V., Naeem, S., Small, R., & Ungvari, J. (2019). Planetary health: from concept to decisive action. The Lancet. Planetary Health, 3(10), e402–e404. <https://doi.org/10.1016/S2542-5196(19)30190-1> | Study design: No methodology - |
| 65 | Haigh, F., Kemp, L., Bazeley, P., & Haigh, N. (2019). Developing a critical realist informed framework to explain how the human rights and social determinants of health relationship works. BMC Public Health, 19(1), 1571. <https://doi.org/10.1186/s12889-019-7760-7> | Study design: No methodology - |
| 66 | Buckley, L., Curtin, M., Maguire, C., Walsh, G., Gibson, L., & Harford, K. (2019). GP88 Enhancing services for vulnerable children and families through public health nurses participation in a workforce capacity building initiative and interagency and partnership in a low-income irish community. Archives of Disease in Childhood, 104(Suppl 3), A65. <https://doi.org/10.1136/archdischild-2019-epa.153> | Study design: No methodology - |
| 67 | Carr, T., Turner, M. W., & Ramaswamy, R. (2019). Scoping review study to identify how communities in the USA, Australia, New Zealand and Canada use quality improvement (QI) approaches to address community health and well-being. *BMJ open*, *9*(12), e034302. https://doi.org/10.1136/bmjopen-2019-034302 | Protocol |
| 68 | Paixão, S. (2019). Environmental health in Portugal and in the world. Annals of Medicine, 51(sup1), 2626. https://doi.org/10.1080/07853890.2018.1560063 | Study design: No methodology - |
| 69 | Bloomfield A. (2019). What Does a Wellbeing Budget Mean for Health and Health Care?. *The Milbank quarterly*, *97*(4), 897–900. https://doi.org/10.1111/1468-0009.12428 | Study design: No methodology - |
| 70 | Gupta, A., Naidu, N. K., & Kakkar, R. (2019). Role of social marketing in promoting primary care to succeed in current era. *Journal of family medicine and primary care*, *8*(10), 3086–3089. https://doi.org/10.4103/jfmpc.jfmpc_514_19 | Study design: No methodology - |
| 71 | Boylan, S., Beyer, K., Schlosberg, D., Mortimer, A., Hime, N., Scalley, B., Alders, R., Corvalan, C., & Capon, A. (2018). A conceptual framework for climate change, health and wellbeing in NSW, Australia. *Public health research & practice*, *28*(4), 2841826. https://doi.org/10.17061/phrp2841826 | No framework with competencies |
| 72 | Greenbaum, V. J., Titchen, K., Walker-Descartes, I., Feifer, A., Rood, C. J., & Fong, H. F. (2018). Multi-level prevention of human trafficking: The role of health care professionals. *Preventive medicine*, *114*, 164–167. https://doi.org/10.1016/j.ypmed.2018.07.006 | Study design: No methodology - |
| 73 | Ford D. E. (2017). The Community and Public Well-being Model: A New Framework and Graduate Curriculum for Addressing Adverse Childhood Experiences. *Academic pediatrics*, *17*(7S), S9–S11. https://doi.org/10.1016/j.acap.2017.04.011 | Study design: No methodology - |
| 74 | Hirani, S. A., & Richter, S. (2017). The capability approach: a guiding framework to improve population health and the attainment of the Sustainable Developmental Goals. *Eastern Mediterranean health journal = La revue de sante de la Mediterranee orientale = al-Majallah al-sihhiyah li-sharq al-mutawassit*, *23*(1), 46–50. https://doi.org/10.26719/2017.23.1.46 | Study design: No methodology - |
| 75 | The determinants of subjective wellbeing: an analysis of a health and wellbeing survey in southeast england | Study design: No methodology - |
| 76 | Buse, K., Tanaka, S., & Hawkes, S. (2017). Healthy people and healthy profits? Elaborating a conceptual framework for governing the commercial determinants of non-communicable diseases and identifying options for reducing risk exposure. *Globalization and health*, *13*(1), 34. https://doi.org/10.1186/s12992-017-0255-3 | No framework with competencies |
| 77 | Viniegra-Velázquez L. (2016). El bien vivir: ¿cuidado de la salud o proyecto vital? Primera parte [To live well: health care or life project? Part I]. *Boletin medico del Hospital Infantil de Mexico*, *73*(2), 139–146. https://doi.org/10.1016/j.bmhimx.2016.01.001 | Study design: No methodology - |
| 78 | Martin, L. T., Plough, A., Carman, K. G., Leviton, L., Bogdan, O., & Miller, C. E. (2016). Strengthening Integration Of Health Services And Systems. *Health affairs (Project Hope)*, *35*(11), 1976–1981. https://doi.org/10.1377/hlthaff.2016.0605 | Study design: No methodology - |
| 79 | Shrivastava, P., Raivio, K., Kasuga, F., Tewksbury, J., Haines, A., & Daszak, P. (2016). Future Earth Health Knowledge-Action Network. *Public health reviews*, *37*, 25. https://doi.org/10.1186/s40985-016-0039-y | Study design: No methodology - |
| 80 | Graham, H., & White, P. C. (2016). Social determinants and lifestyles: integrating environmental and public health perspectives. *Public health*, *141*, 270–278. https://doi.org/10.1016/j.puhe.2016.09.019 | Study design: No methodology - |
| 81 | Nash D. B. (2015). Population health: where's the beef?. *Population health management*, *18*(1), 1–3. https://doi.org/10.1089/pop.2015.1811 | Study design: No methodology - |
| 82 | Reis, S., Morris, G., Fleming, L. E., Beck, S., Taylor, T., White, M., Depledge, M. H., Steinle, S., Sabel, C. E., Cowie, H., Hurley, F., Dick, J. M., Smith, R. I., & Austen, M. (2015). Integrating health and environmental impact analysis. *Public health*, *129*(10), 1383–1389. https://doi.org/10.1016/j.puhe.2013.07.006 | Study design: No methodology - |
| 83 | 'Gp champions' in England; family physicians and youth workers collaborate in a new model of youthfriendly primary care practice to address unmet need | Study design: No methodology - |
| 84 | Hinton, R., Kavanagh, D. J., Barclay, L., Chenhall, R., & Nagel, T. (2015). Developing a best practice pathway to support improvements in Indigenous Australians' mental health and well-being: a qualitative study. *BMJ open*, *5*(8), e007938. https://doi.org/10.1136/bmjopen-2015-007938 | No framework with competencies |
| 85 | Whitmee, S., Haines, A., Beyrer, C., Boltz, F., Capon, A. G., de Souza Dias, B. F., Ezeh, A., Frumkin, H., Gong, P., Head, P., Horton, R., Mace, G. M., Marten, R., Myers, S. S., Nishtar, S., Osofsky, S. A., Pattanayak, S. K., Pongsiri, M. J., Romanelli, C., Soucat, A., … Yach, D. (2015). Safeguarding human health in the Anthropocene epoch: report of The Rockefeller Foundation-Lancet Commission on planetary health. *Lancet (London, England)*, *386*(10007), 1973–2028. https://doi.org/10.1016/S0140-6736(15)60901-1 | Study design: No methodology - |
| 86 | Ruger, J. P., Hammonds, R., Ooms, G., Barry, D., Chapman, A., & Van Damme, W. (2015). From conceptual pluralism to practical agreement on policy: global responsibility for global health. *BMC international health and human rights*, *15*, 30. https://doi.org/10.1186/s12914-015-0065-8 | No study design- No methodology- |
| 87 | Lapinski, M. K., Funk, J. A., & Moccia, L. T. (2015). Recommendations for the role of social science research in One Health. *Social science & medicine (1982)*, *129*, 51–60. https://doi.org/10.1016/j.socscimed.2014.09.048 | Study design: No methodology - |
| 88 | Raising the profile of health and well-being education in teacher training: The challenges and successes of introducing a skills-based Health Day for pre-service teachers at the University of Southampton | No framework with competencies |
| 89 | One medicine - One health: For humans, animals and environment | Study design: No methodology - |
| 90 | Ottersen, O. P., Dasgupta, J., Blouin, C., Buss, P., Chongsuvivatwong, V., Frenk, J., Fukuda-Parr, S., Gawanas, B. P., Giacaman, R., Gyapong, J., Leaning, J., Marmot, M., McNeill, D., Mongella, G. I., Moyo, N., Møgedal, S., Ntsaluba, A., Ooms, G., Bjertness, E., Lie, A. L., … Scheel, I. B. (2014). The political origins of health inequity: prospects for change. *Lancet (London, England)*, *383*(9917), 630–667. <https://doi.org/10.1016/S0140-6736(13)62407-1> | Study design: No methodology - |
| 91 | Langford, R., Bonell, C. P., Jones, H. E., Pouliou, T., Murphy, S. M., Waters, E., Komro, K. A., Gibbs, L. F., Magnus, D., & Campbell, R. (2014). The WHO Health Promoting School framework for improving the health and well-being of students and their academic achievement. *The Cochrane database of systematic reviews*, *2014*(4), CD008958. https://doi.org/10.1002/14651858.CD008958.pub2 | No framework with competencies |
| 92 | Hancock T. (2002). Indicators of environmental health in the urban setting. *Canadian journal of public health = Revue canadienne de sante publique*, *93 Suppl 1*(Suppl 1), S45–S51. https://doi.org/10.1007/BF03405118 | No study design- No methodology- |
| 93 | Prilleltensky I. (2005). Promoting well-being: time for a paradigm shift in health and human services1. *Scandinavian journal of public health. Supplement*, *66*, 53–60. https://doi.org/10.1080/14034950510033381 | Study design: No methodology - |
| 94 | VanderPlaat, M., & Teles, N. (2005). Mainstreaming social justice: human rights and public health. *Canadian journal of public health = Revue canadienne de sante publique*, *96*(1), 34–36. https://doi.org/10.1007/BF03404011 | Study design: No methodology - |
| 95 | Health education, prevention, health promotion | No access to full text |
| 96 | Kauppinen, T., Nelimarkka, K., & Perttilä, K. (2006). The effectiveness of human impact assessment in the Finnish Healthy Cities Network  . *Public health*, *120*(11), 1033–1041. https://doi.org/10.1016/j.puhe.2006.05.028 | No framework with competencies |
| 97 | Kennedy E. T. (2006). Evidence for nutritional benefits in prolonging wellness. *The American journal of clinical nutrition*, *83*(2), 410S–414S. https://doi.org/10.1093/ajcn/83.2.410S | Study design: No methodology - |
| 98 | Carpiano R. M. (2006). Toward a neighborhood resource-based theory of social capital for health: can Bourdieu and sociology help?. *Social science & medicine (1982)*, *62*(1), 165–175. https://doi.org/10.1016/j.socscimed.2005.05.020 | Study design: No methodology - |
| 99 | Maibach, E. W., Roser-Renouf, C., & Leiserowitz, A. (2008). Communication and marketing as climate change-intervention assets a public health perspective. *American journal of preventive medicine*, *35*(5), 488–500. https://doi.org/10.1016/j.amepre.2008.08.016 | Study design: No methodology - |
| 100 | Berry, H. L., Bowen, K., & Kjellstrom, T. (2010). Climate change and mental health: a causal pathways framework. *International journal of public health*, *55*(2), 123–132. https://doi.org/10.1007/s00038-009-0112-0 | No framework with competencies |
| 101 | Hanlon, P., Carlisle, S., Reilly, D., Lyon, A., & Hannah, M. (2010). Enabling well-being in a time of radical change: Integrative public health for the 21st century. *Public health*, *124*(6), 305–312. https://doi.org/10.1016/j.puhe.2010.03.014 | Study design: No methodology - |
| 102 | Nurse, J., Basher, D., Bone, A., & Bird, W. (2010). An ecological approach to promoting population mental health and well-being--a response to the challenge of climate change. *Perspectives in public health*, *130*(1), 27–33. https://doi.org/10.1177/1757913909355221 | Study design: No methodology - |
| 103 | Zinsstag, J., Schelling, E., Waltner-Toews, D., & Tanner, M. (2011). From "one medicine" to "one health" and systemic approaches to health and well-being. *Preventive veterinary medicine*, *101*(3-4), 148–156. https://doi.org/10.1016/j.prevetmed.2010.07.003 | Study design: No methodology - |
| 104 | Hambling T, Weinstein P, Slaney D. A review of frameworks for developing environmental health indicators for climate change and health. Int J Environ Res Public Health. 2011 Jul;8(7):2854-75. doi: 10.3390/ijerph8072854. Epub 2011 Jul 13. PMID: 21845162; PMCID: PMC3155333. | Study design: No methodology - |
| 105 | Hanlon, P., Carlisle, S., Hannah, M., Lyon, A., & Reilly, D. (2012). A perspective on the future public health: an integrative and ecological framework. *Perspectives in public health*, *132*(6), 313–319. https://doi.org/10.1177/1757913912440781 | Study design: No methodology - |
| 106 | Global health education locally: A community servicelearning program to support refugees, engage medical students, and fill a gap in the community | Study design: No methodology - |
| 107 | Schuchter, J., & Jutte, D. P. (2014). A framework to extend community development measurement to health and well-being. *Health affairs (Project Hope)*, *33*(11), 1930–1938. https://doi.org/10.1377/hlthaff.2014.0961 | Study design: No methodology - |
| 108 | Does it do what it says on the tin? Health impact assessment (HIA) of the northern Ireland cardiovascular service framework | No access to full text |
| 109 | Partnerships in the South for a stronger international network | No access to full text |
| 110 | Fudge, E. A., & Robinson, P. (2009). A public health approach to promoting better mental health outcomes for children of parents with a psychiatric disability. Psychiatric Rehabilitation Journal, 33(2), 83–90. https://doi.org/10.2975/33.2.2009.83.90 | Study design: No methodology - |
| 111 | Middelbeek L., Kahlmeier S., & Wijnhoven T. (2010). WHO strategies to increase active living and reduce sedentary behaviour. International Journal of Pediatric Obesity, 5(SUPPL. 1), 10–11. https://doi.org/10.3109/17477160903387715 | Study design: No methodology - |
| 112 | Swanson, K. M., & Wojnar, D. M. (2004). Optimal healing environments in nursing. *Journal of alternative and complementary medicine (New York, N.Y.)*, *10 Suppl 1*, S43–S48. https://doi.org/10.1089/1075553042245935 | Study design: No methodology - |
| 113 | F. Ladak, N. Chan, & J. Pedersen. (2014). Students for Health Innovation and Education (SHINE): Fostering leadership among medical students and residents. Annals of Global Health, 80(3), 167–168. https://doi.org/10.1016/j.aogh.2014.08.034 | Study design: No methodology - |
| 114 | Odom, S. K., Jackson, P., Derauf, D., Inada, M. K., & Aoki, A. H. (2019). Pilinahā: An Indigenous Framework for Health. *Current developments in nutrition*, *3*(Suppl 2), 32–38. https://doi.org/10.1093/cdn/nzz001 | No framework with competencies |
| 115 | C. Robinette, L. Saffran, A. Ruple, & S.L. Deem. (2017). Zoos and public health: A partnership on the One Health frontier. One Health, 3(C), 1–4. https://doi.org/10.1016/j.onehlt.2016.11.003 | Study design: No methodology - |
| 116 | Chura, L. R., & Lock, S. L. (2016). HOW THE GLOBAL COUNCIL ON BRAIN HEALTH WILL TACKLE BEHAVIOR CHANGE: STRATEGIES FOR PROMOTING WELL-BEING ACROSS THE LIFESPAN. Alzheimer’s & Dementia, 12(7), P996. | Study design: No methodology - |
| 117 | Generation U: A government response involving a unique connected range of policy, programs and partnerships, to improve the health and wellbeing of the next generation | No access to full text |
| 118 | Francis, T., Peiris, R. G., Stanimirovic, A., Stogios, N., Syed, F., Rac, V. E., Nolan, R. P., & Sarfraz, M. (2024). The EvalUation of goal-diRected activities to prOmote well-beIng and heAlth (EUROIA) scale: Psychometric evaluation with Confirmatory Factor Analysis. Plos One, 19(3). https://doi.org/10.1371/journal.pone.0299854 | No framework with competencies |
| 119 | Ogutu, M. O., Kamui, E., Abuya, T., & Muraya, K. (2023). "We are their eyes and ears here on the ground, yet they do not appreciate us"-Factors influencing the performance of Kenyan community health volunteers working in urban informal settlements. *PLOS global public health*, *3*(8), e0001815. https://doi.org/10.1371/journal.pgph.0001815 | No framework with competencies |
| 120 | Flodgren, G., Helleve, A., Selstø, A., Fismen, A.-S., Blanchard, L., Rutter, H., & Klepp, K.-I. (2024). Youth involvement in policy processes in public health, education, and social work-A scoping review. Obesity Reviews : An Official Journal of the International Association for the Study of Obesity, e13874. https://doi.org/10.1111/obr.13874 | No framework with competencies |
| 121 | Abu-Ras, W., Aboul-Enein, B. H., Almoayad, F., Benajiba, N., & Dodge, E. (2024). Mosques and Public Health Promotion: A Scoping Review of Faith-Driven Health Interventions. Health Education & Behavior, 51(5), 677–690. https://doi.org/10.1177/10901981241252800 | No framework with competencies |
| 122 | Wahi, G., Kandasamy, S., Bangdiwala, S. I., Baumann, A., Crea-Arsenio, M., Desai, D., DiLiberto, D., Georgiades, K., Jackson-Best, F., Kwan, M., Montague, P., Newbold, K. B., Sherifali, D., Sim, A., de Souza, R. J., Anand, S. S., & Carels, V. (2023). Strengthening Community Roots: Anchoring Newcomers in Wellness and Sustainability (SCORE!): A protocol for the co-design and evaluation of a healthy active living program among a newcomer community in Canada. Plos One, 18(9). https://doi.org/10.1371/journal.pone.0288851 | protocol. |
| 123 | O’Neill, K., Hand, R., Diop, B., Weiss, H., Cruz Pfaeffle, A., Maragatham, P., Rice, K., & Naslund, J. A. (2023). Informing the development of the coaching online and community health (COACH) program: a qualitative study of clubhouse members living with serious mental illness. Translational Behavioral Medicine, 13(5), 343–353. https://doi.org/10.1093/tbm/ibad001 | No framework with competencies |
| 124 | Shepley, M. M., Ames, R. L., Leitão, R. M., & Coleman, G. (2023). Community-based participatory research on the impact of greenspace on violent crime. Environmental Research: Health, 1(2). https://doi.org/10.1088/2752-5309/aca2d2 | No framework with competencies |
| 125 | Sprengel, M., & Fritts, M. (2012). OA13.02. Utilizing mind-body practices in public schools: teaching self-regulation skills and fostering resilience in our next generation. BMC Complementary and Alternative Medicine : The Official Journal of the International Society for Complementary Medicine Research (ISCMR), 12(1), 1. https://doi.org/10.1186/1472-6882-12-S1-O50 | Study design: No methodology - |
| 126 | **J**. B. Spiegel, B. Ortiz Choukroun, A. Campaña, K. M. Boydell, J. Breilh, & A. Yassi. (2019). Social transformation, collective health and community-based arts: ‘Buen Vivir’ and Ecuador’s social circus programme. Global Public Health, 14(6-7), 899–922. https://doi.org/10.1080/17441692.2018.1504102 | No framework with competencies |
| 127 | David E. Beal, Vicki L. Simpson, Brandon J. Pitts, & Jennifer A. Coddington. (2024). Identifying barriers to implementation of the medicare annual wellness visit for patients of a rural federally qualified health center. Human Factors in Healthcare, 5, 100077–. https://doi.org/10.1016/j.hfh.2024.100077 | No framework with competencies |
| 128 | Whitehead, M., Pennington, A., Orton, L., Nayak, S., Petticrew, M., Sowden, A., & White, M. (2016). How could differences in 'control over destiny' lead to socio-economic inequalities in health? A synthesis of theories and pathways in the living environment. *Health & place*, *39*, 51–61. https://doi.org/10.1016/j.healthplace.2016.02.002 | No framework with competencies |
| 129 | Sarmiento Loayza L. (2022). Bienestar subjetivo, estado de salud, funcionalidad y participación social en personas mayores de Chile [Subjective well-being, health status, functional capacity and social participation in Chilean older people]. *Revista medica de Chile*, *150*(8), 1010–1017. https://doi.org/10.4067/S0034-98872022000801010 | Language |
| 130 | Tallman, P. S., Piland, N. C., Villarmarzo, M., Victoria-Lacy, L., & Valdés-Velásquez, A. (2024). Waterscapes meet socio-ecological models: A relational framework to examine water insecurity and human health and well-being. People and Nature, 6(3), 1036–1047. https://doi.org/10.1002/pan3.10635 | Study design: No methodology - |
| 131 | Blezer, S., Abujidi, N., & Sap, H. (2023). Urban living labs as innovation infrastructure for local urban intervention acceleration and student social learning: the impacts on community wellbeing in Heerlen. Frontiers in Public Health, 11, 1242151. https://doi.org/10.3389/fpubh.2023.1242151 | Study design: No methodology - |
| 132 | Noonan, R. J. (2024). Extrinsic goals benefit capitalism but not well-being. Rethinking the economy’s goal for a healthier future. Health Promotion International, 39(5). https://doi.org/10.1093/heapro/daae090 | Study design: No methodology - |
| 133 | Kruk, M. E., Lewis, T. P., Arsenault, C., Bhutta, Z. A., Irimu, G., Jeong, J., Lassi, Z. S., Sawyer, S. M., Vaivada, T., Waiswa, P., & Yousafzai, A. K. (2022). Improving health and social systems for all children in LMICs: structural innovations to deliver high-quality services. Lancet (London, England), 399(10337), 1830–1844. https://doi.org/10.1016/S0140-6736(21)02532-0 | Study design: No methodology - |
| 134 | Rosenberg, J., McDonough Ryan, P., O’Brien, C., Ganjavi, F., & Sharifi, M. (2022). Pilot Wellness Program With Adapted Social-Emotional Learning and COVID-19 Curriculum for Refugee Youth. Health Education & Behavior : The Official Publication of the Society for Public Health Education, 49(1), 17–25. https://doi.org/10.1177/10901981211048830 | No framework with competencies |
| 135 | Biglan, A., Flay, B. R., Embry, D. D., & Sandler, I. N. (2012). The Critical Role of Nurturing Environments for Promoting Human Well-Being. American Psychologist, 67(4), 257–271. | Study design: No methodology - |
| 136 | Kelly, M. P., & Carr, A. L. (2023). The ten steps for acting on health inequalities. Public Health in Practice, 6. https://doi.org/10.1016/j.puhip.2023.100422 | Study design: No methodology - |
| 137 | Von Heimburg, D., & Ness, O. (2021). Relational welfare: a socially just response to co-creating health and wellbeing for all. Scandinavian Journal of Public Health, 49(6), 639–652. https://doi.org/10.1177/1403494820970815 | Study design: No methodology - |
| 138 | Koch, P., Kersten, J. F., & Nienhaus, A. (2023). Monitoring a cohort of trainees: changes over time and associations between health literacy, health behaviour and health. Journal of Occupational Medicine and Toxicology, 18(1). https://doi.org/10.1186/s12995-023-00387-1 | No framework with competencies |
| 139 | Welden, E. A., Chausson, A., & Melanidis, M. S. (2021). Leveraging Nature-based Solutions for transformation: Reconnecting people and nature. People and Nature, 3(5), 966–977. https://doi.org/10.1002/pan3.10212 | Study design: No methodology - |
| 140 | Montesanti, S., Fitzpatrick, K., Fayant, B., & Pritchard, C. (2022). Identifying priorities, directions and a vision for Indigenous mental health using a collaborative and consensus-based facilitation approach. BMC Health Services Research, 22(1). https://doi.org/10.1186/s12913-022-07682-3 | No framework with competencies |
| 141 | Sara Hudson, Dennis Foley, & Margaret Cargo. (2022). Indigenous Social Enterprises and Health and Wellbeing: A Scoping Review and Conceptual Framework. International Journal of Environmental Research and Public Health, 19(14478), 14478. <https://doi.org/10.3390/ijerph192114478> | No framework with competencies |
| 142 | Melissa R. Marselle, Terry Hartig, Daniel T.C. Cox, Siân de Bell, Sonja Knapp, Sarah Lindley, Margarita Triguero-Mas, Katrin Böhning-Gaese, Matthias Braubach, Penny A. Cook, Sjerp de Vries, Anna Heintz-Buschart, Max Hofmann, Katherine N. Irvine, Nadja Kabisch, Franziska Kolek, Roland Kraemer, Iana Markevych, Dörte Martens, et al. (2021). Pathways linking biodiversity to human health: A conceptual framework. Environment International, 150, 106420. | No framework with competencies |
| 143 | Chastin, S. F., De Craemer, M., Lien, N., Bernaards, C., Buck, C., Oppert, J. M., Nazare, J. A., Lakerveld, J., O'Donoghue, G., Holdsworth, M., Owen, N., Brug, J., Cardon, G., & DEDIPAC consortium, expert working group and consensus panel (2016). The SOS-framework (Systems of Sedentary behaviours): an international transdisciplinary consensus framework for the study of determinants, research priorities and policy on sedentary behaviour across the life course: a DEDIPAC-study. *The international journal of behavioral nutrition and physical activity*, *13*, 83. <https://doi.org/10.1186/s12966-016-0409-3> | No framework with competencies |
| 144 | Macaulay, B., Roy, M. J., Donaldson, C., Teasdale, S., & Kay, A. (2018). Conceptualizing the health and well-being impacts of social enterprise: a UK-based study. *Health promotion international*, *33*(5), 748–759. https://doi.org/10.1093/heapro/dax009 | No framework with competencies |
| 145 | Ooms, T., Klaser, K., & Ishkanian, A. (2023). The role of academia practice partnerships in the well-being economy: Retracing synergies between health and social sciences using bibliometric analysis. *Health policy (Amsterdam, Netherlands)*, *138*, 104936. <https://doi.org/10.1016/j.healthpol.2023.104936> | No framework with competencies |
| 146 | Wallerich, L., Fillol, A., Rivadeneyra, A., Vandentorren, S., Wittwer, J., & Cambon, L. (2023). Environment and child well-being: A scoping review of reviews to guide policies. Health Promotion Perspectives, 13(3), 168–182. <https://doi.org/10.34172/hpp.2023.20> | No framework with competencies |
| 147 | Tamara Mackean, Madison Shakespeare, & Matthew Fisher. (2022). Indigenous and Non-Indigenous Theories of Wellbeing and Their Suitability for Wellbeing Policy. International Journal of Environmental Research and Public Health, 19(11693), 11693. <https://doi.org/10.3390/ijerph191811693> | No framework with competencies |
| 148 | Stephen Nwoba Ukpai. (2022). Water policy reform in the Nigeria water governance system: assessment of water resources management based on OECD Principles on Water Governance. Water Policy, 24(10), 1704–1722. <https://doi.org/10.2166/wp.2022.135> | No framework with competencies |
| 149 | Delany-Crowe, T., Marinova, D., Fisher, M., McGreevy, M., & Baum, F. (2019). Australian policies on water management and climate change: are they supporting the sustainable development goals and improved health and well-being?. *Globalization and health*, *15*(1), 68. <https://doi.org/10.1186/s12992-019-0509-3> | No framework with competencies |
| 150 | Tomlinson, P., Hewitt, S., & Blackshaw, N. (2013). Joining up health and planning: how Joint Strategic Needs Assessment (JSNA) can inform health and wellbeing strategies and spatial planning. *Perspectives in public health*, *133*(5), 254–262. https://doi.org/10.1177/1757913913488331 | No framework with competencies |
| 151 | Crealey, G., McQuade, L., O'Sullivan, R., & O'Neill, C. (2023). Arts and creativity interventions for improving health and wellbeing in older adults: a systematic literature review of economic evaluation studies. *BMC public health*, *23*(1), 2496. https://doi.org/10.1186/s12889-023-17369-x | No framework with competencies |
| 152 | Navas-Martín, M. Á., Cuerdo-Vilches, T., López-Bueno, J. A., Díaz, J., Linares, C., & Sánchez-Martínez, G. (2024). Human adaptation to heat in the context of climate change: A conceptual framework. *Environmental research*, *252*(Pt 1), 118803. https://doi.org/10.1016/j.envres.2024.118803 | No framework with competencies |
| 153 | Schultz, T. R., Edemba, D., Lecuyer, M. E., Garzone, A., Henise, S., Liljestrand, A., Hinds, P. S., & King, S. (2024). LGBTQ+ Population Well-Being: Health Equity Implementation Science in Action. Public Health Nursing (Boston, Mass.). <https://doi.org/10.1111/phn.13480> | No framework with competencies |
| 154 | Myhre, S. L., Scobie, M., Meriläinen, E., Kelman, I., & Gopinathan, U. (2024). Climate Change, Community Action, and Health in the Anglophone Caribbean: A Scoping Review. Public Health Reviews, 44. <https://doi.org/10.3389/phrs.2023.1605843> | No framework with competencies |
| 155 | Buttazzoni, A., Doherty, S., & Minaker, L. (2022). How Do Urban Environments Affect Young People’s Mental Health? A Novel Conceptual Framework to Bridge Public Health, Planning, and Neurourbanism. Public Health Reports (Washington, D.C. : 1974), 137(1), 48–61. <https://doi.org/10.1177/0033354920982088> Tiene metodologia y competencias | No framework with competencies |
| 156 | Baum, F., Newman, L., & Biedrzycki, K. (2014). Vicious cycles: digital technologies and determinants of health in Australia. Health Promotion International, 29(2), 349–360. <https://doi.org/10.1093/heapro/das062> | No framework with competencies |
| 157 | Waller, D., Brooks, F., Perry, L., Kang, M., & Steinbeck, K. (2021). Australian federal, state and territory policy on the health and wellbeing of young people: A scoping review. *Health policy (Amsterdam, Netherlands)*, *125*(8), 1065–1076. <https://doi.org/10.1016/j.healthpol.2021.05.017> | No framework with competencies |
| 158 | Footer, K., Windle, M., Ferguson, L., Hatcher, J., Lyons, C., Gorin, E., Stangl, A. L., Golub, S., Gruskin, S., & Baral, S. (2018). A Meta-Narrative Literature Synthesis and Framework to Guide Future Evaluation of Legal Empowerment Interventions. *Health and human rights*, *20*(2), 65–84. | No framework with competencies |
| 159 | Romero, D., Echeverria, S. E., Duffy, M., Roberts, L., & Pozen, A. (2018). Development of a wellness trust to improve population health: Case-study of a United States urban center. *Preventive medicine reports*, *10*, 292–298. https://doi.org/10.1016/j.pmedr.2018.03.009 | No framework with competencies |
| 160 | Lokmic-Tomkins, Z., Barbour, L., LeClair, J., Luebke, J., McGuinness, S. L., Limaye, V. S., Pillai, P., Flynn, M., Kamp, M. A., Leder, K., & Patz, J. A. (2024). Integrating planetary health education into tertiary curricula: a practical toolbox for implementation. *Frontiers in medicine*, *11*, 1437632. https://doi.org/10.3389/fmed.2024.1437632 | No framework with competencies |

**Table 2.** Results from organizations website search such as the World Health Organization, the Wellbeing Economy Alliance, among others ( Second mechanism: Website)

| # | Reference | Include or exclude |
| --- | --- | --- |
| 1 | Country deep dive on the well-being economy: Wales. Copenhagen: WHO Regional Office for Europe; 2024. Licence: CC BY-NC-SA 3.0 IGO. [WHO-EURO-2024-10150-49922-75119-eng.pdf](https://iris.who.int/bitstream/handle/10665/378205/WHO-EURO-2024-10150-49922-75119-eng.pdf?sequence=1) | Framework without competencies: Excluded |
| 2 | Shifting towards fiscally resilient, healthy societies: finding common ground between public health and finance sectors and central banks. Copenhagen: WHO Regional Office for Europe; 2024. Licence: CC BY-NC-SA 3.0 IGO. [WHO-EURO-2024-9549-49321-73721-eng.pdf](https://iris.who.int/bitstream/handle/10665/376415/WHO-EURO-2024-9549-49321-73721-eng.pdf?sequence=1) | Study design without methods: Excluded |
| 3 | World Health Organization. Regional Office for Europe. (‎2024)‎. Political statement of the WHO European Healthy Cities Network: towards thriving societies - advancing health in the well-being economy. World Health Organization. Regional Office for Europe. <https://iris.who.int/handle/10665/376153>. License: CC BY-NC-SA 3.0 IGO | Framework without competencies: Excluded |
| 4 | Country deep dive on the well-being economy: Iceland. Copenhagen: WHO Regional Office for Europe; 2023. Licence: CC BY-NC-SA 3.0 IGO [WHO-EURO-2023-7415-47181-69111-eng.pdf](https://iris.who.int/bitstream/handle/10665/369105/WHO-EURO-2023-7415-47181-69111-eng.pdf?sequence=1) | Included |
| 5 | World Health Organization. Regional Office for Europe. (‎2019)‎. Assessing the health impacts of a circular economy. World Health Organization. Regional Office for Europe. <https://iris.who.int/handle/10665/346222> | Framework without competencies: Excluded |
| 6 | Health in the well-being economy. Background paper: working together to achieve healthy, fairer, prosperous societies across the WHO European Region. Copenhagen: WHO Regional Office for Europe; 2023. Licence: CC BY-NC-SA 3.0 IGO. [WHO-EURO-2023-7144-46910-68439-eng.pdf](https://iris.who.int/bitstream/handle/10665/366228/WHO-EURO-2023-7144-46910-68439-eng.pdf?sequence=1) | Framework without competencies: Excluded |
| 7 | Deep dives on the well-being economy showcasing the experiences of Finland, Iceland, Scotland and Wales: summary of key findings. Copenhagen: WHO Regional Office for Europe; 2023. Licence: CC BY-NC-SA 3.0 IGO. [WHO-EURO-2023-7033-46799-68216-eng.pdf](https://iris.who.int/bitstream/handle/10665/366279/WHO-EURO-2023-7033-46799-68216-eng.pdf?sequence=3) | Framework without competencies: Excluded |
| 8 | Economic and social impacts and benefits of heaLth systems [WHO-EURO-2019-3521-43280-60657-eng.pdf](https://iris.who.int/bitstream/handle/10665/346019/WHO-EURO-2019-3521-43280-60657-eng.pdf?sequence=3) | Framework without competencies:: Excluded |
| 9 | World Health Organization. Regional Office for Europe. (‎2024)‎. Roadmap for health and well-being in Central Asia (‎2022–2025)‎: progress and opportunities. World Health Organization. Regional Office for Europe. <https://iris.who.int/handle/10665/377613>. License: CC BY-NC-SA 3.0 IGO | Framework without competencies: Excluded |
| 10 | World Health Organization. Regional Office for Europe. (‎2022)‎. Roadmap for health and well-being in Central Asia (‎2022–2025)‎. World Health Organization. Regional Office for Europe. <https://iris.who.int/handle/10665/364330>. License: CC BY-NC-SA 3.0 IGO | Framework without competencies: Excluded |
| 11 | World Health Organization. Regional Office for Europe. (‎2018)‎. Multisectoral and intersectoral action for improved health and well-being for all: mapping of the WHO European Region. Governance for a sustainable future: improving health and well-being for all: final report. World Health Organization. Regional Office for Europe. <https://iris.who.int/handle/10665/341715> | Framework without competencies: Excluded |
| 12 | World Health Organization. Regional Office for Europe. (‎2024)‎. Elevating health and well-being in the United Nations Sustainable Development Framework: updated guidance 2024. World Health Organization. Regional Office for Europe. <https://iris.who.int/handle/10665/379865>. License: CC BY-NC-SA 3.0 IGO | Study design without methods: Excluded |
| 13 | World Health Organization. Regional Office for Europe. (‎2023)‎. Annual progress report towards the implementation of the Roadmap for health and well-being in the Western Balkans (‎2021–2025)‎. World Health Organization. Regional Office for Europe. <https://iris.who.int/handle/10665/369671>. License: CC BY-NC-SA 3.0 IGO | Framework without competencies:: Excluded |
| 14 | World Health Organization. Regional Office for Europe. (‎2022)‎. Supporting mainstreaming and elevating health and well-being in the United Nations Sustainable Development Framework: guidance note. World Health Organization. Regional Office for Europe. <https://iris.who.int/handle/10665/357652>. License: CC BY-NC-SA 3.0 IGO | Framework without competencies: Excluded |
| 15 | World Health Organization. Regional Office for Europe. (‎2013)‎. Health 2020: a European policy framework supporting action across government and society for health and well-being (‎short version)‎. World Health Organization. Regional Office for Europe. <https://iris.who.int/handle/10665/131300> | Included |
| 16 | World Health Organization. Regional Office for Europe. (‎2020)‎. Health and well-being in the voluntary national reviews of the 2030 Agenda for Sustainable Development in the WHO European Region 2016–2020. World Health Organization. Regional Office for Europe. <https://iris.who.int/handle/10665/340007>. License: CC BY-NC-SA 3.0 IGO | Included |
| 17 | World Health Organization. Regional Office for Europe. (‎2015)‎. National healthy cities networks in the WHO European Region: promoting health and well-being throughout Europe. World Health Organization. Regional Office for Europe. <https://iris.who.int/handle/10665/344404> | Framework without competencies: Excluded |
| 18 | World Health Organization. Regional Office for Europe. (‎2021)‎. Roadmap for Health and Well-being in the Western Balkans (‎2021-2025)‎: European Programme of Work (‎2020–2025)‎ - United Action for Better Health. World Health Organization. Regional Office for Europe. <https://iris.who.int/handle/10665/345932>. License: CC BY-NC-SA 3.0 IGO | Framework without competencies: Excluded |
| 19 | Enhancing Access to and Sharing of Data in the Age of Artificial Intelligence [Enhancing Access to and Sharing of Data in the Age of Artificial Intelligence \| OECD](https://www.oecd.org/en/publications/enhancing-access-to-and-sharing-of-data-in-the-age-of-artificial-intelligence_23a70dca-en.html) | Study design without methods: Excluded |
| 20 | Scotland’s Wellbeing Economy Toolkit for local authorities [Scotland’s Wellbeing Economy Toolkit for local authorities \| OECD](https://www.oecd.org/en/publications/well-being-knowledge-exchange-platform-kep_93d45d63-en/scotland-s-wellbeing-economy-toolkit-for-local-authorities_481c492a-en.html) | Study design without methods: Excluded |
| 21 | Wellbeing Economy Toolkit Supporting place-based economic strategy and policy development.  [Executive Summary - Wellbeing economy toolkit: supporting place based economic strategy and policy development - gov.scot](https://www.gov.scot/publications/wellbeing-economy-toolkit-supporting-place-based-economic-strategy-policy-development/pages/1/) | Included as a unit |
| 22 | [wellbeing-economy-monitor-guidance-updated-february-2024.docx](https://view.officeapps.live.com/op/view.aspx?src=https%3A%2F%2Fwww.gov.scot%2Fbinaries%2Fcontent%2Fdocuments%2Fgovscot%2Fpublications%2Fadvice-and-guidance%2F2022%2F11%2Fwellbeing-economy-toolkit-supporting-place-based-economic-strategy-policy-development%2Fdocuments%2Fwellbeing-economy-monitor-guidance-updated-february-2024%2Fwellbeing-economy-monitor-guidance-updated-february-2024%2Fgovscot%253Adocument%2Fwellbeing-economy-monitor-guidance-updated-february-2024.docx&wdOrigin=BROWSELINK) |  |
| 23 | Scotland’s National Performance Framework Our Purpose, Values and National Outcomes [National Performance Framework \| National Performance Framework](https://nationalperformance.gov.scot/) [NPF_A4_Booklet.pdf](https://nationalperformance.gov.scot/sites/default/files/documents/NPF_A4_Booklet.pdf) |  |
| 24 | Concept note for the OECD Guidance on Reducing Poverty and Inequalities through Development Co-operation [pdf](https://one.oecd.org/document/DCD(2025)1/en/pdf) | Framework without competencies: Excluded |
| 25 | Working Party on Artificial Intelligence Governance Assessing potential future artificial intelligence risks, benefits and policy imperatives Perspectives from the OECD Expert Group on AI Futures [pdf](https://one.oecd.org/document/DSTI/CDEP/AIGO(2023)13/FINAL/en/pdf) | Included |
| 26 | Llena-Nozal, A., N. Martin and F. Murtin (2019), “The economy of well-being: Creating opportunities for people’s well-being and economic growth”, *OECD Statistics Working Papers*, No. 2019/02, OECD Publishing, Paris, <https://doi.org/10.1787/498e9bc7-en>. | Framework without competencies: Excluded |
| 27 | Developing Skills for Digital Government: A review of good practices across OECD governments [pdf](https://one.oecd.org/document/DELSA/ELSA/WD/SEM(2024)1/en/pdf) | Included |
| 28 | Gottschalk, F. and H. Borhan (2023), “Child participation in decision making: Implications for education and beyond”, *OECD Education Working Papers*, No. 301, OECD Publishing, Paris, <https://doi.org/10.1787/a37eba6c-en>. | Framework without competencies: Excluded |
| 29 | Blazey, A., M. Lelong and F. Giannini (2022), “The Equitable and Sustainable Well-being Framework in Italy: An Action Plan for its use in policy and budget decision making”, *OECD Working Papers on Public Governance*, No. 56, OECD Publishing, Paris, <https://doi.org/10.1787/4f48c504-en>. | Framework without competencies: Excluded |
| 30 | OECD (2022), “Building better societies through digital policy: Background paper for the CDEP Ministerial meeting”, *OECD Digital Economy Papers*, No. 338, OECD Publishing, Paris, <https://doi.org/10.1787/07c3eb90-en>. | Framework without competencies: Excluded |
| 31 | Lee, J. and Ž. Žarnic (2024), “The impact of digital technologies on well-being: Main insights from the literature”, *OECD Papers on Well-being and Inequalities*, No. 29, OECD Publishing, Paris, <https://doi.org/10.1787/cb173652-en>. | Include |
| 32 | Mahoney, J. et al. (2024), “Measuring social connectedness in OECD countries: A scoping review”, *OECD Papers on Well-being and Inequalities*, No. 28, OECD Publishing, Paris, <https://doi.org/10.1787/f758bd20-en>. | Framework without competencies: Excluded |
| 33 | OECD (2020), *How's Life? 2020: Measuring Well-being*, OECD Publishing, Paris, <https://doi.org/10.1787/9870c393-en>. | Framework without competencies: Excluded |
| 34 | Well-being at the heart of policy: lessons from national initiatives around the OECD [Microsoft Word - Brandt-Fleischer.docx](https://newforum.org/wp-content/uploads/2022/02/FNE-BP01-2022.pdf) | Framework without competencies: Excluded |
| 35 | New Zealand’s Living Standards Framework and He Ara Waiora Framework [**New Zealand’s Living Standards Framework and He Ara Waiora Framework \| OECD**](https://www.oecd.org/en/publications/well-being-knowledge-exchange-platform-kep_93d45d63-en/new-zealand-s-living-standards-framework-and-he-ara-waiora-framework_dfce8af5-en.html) | Framework without competencies: Excluded |
| 36 | New Zealand Treasury (2023), “He Ara Waoira”, <https://www.treasury.govt.nz/information-and-services/nz-economy/higher-living-standards/he-ara-waiora> | Framework without competencies: Excluded |
| 37 | New Zealand Treasury (2023), “Using the LSF and He Ara Waiora”, <https://www.treasury.govt.nz/information-and-services/nz-economy/higher-living-standards/using-lsf-and-he-ara-waiora> | Framework without competencies: Excluded |
| 38 | New Zealand Treasury (2022), “Our Living Standards Framework”, <https://www.treasury.govt.nz/information-and-services/nz-economy/higher-living-standards/our-living-standards-framework> | Framework without competencies: Excluded |
| 39 | New Zealand Treasury (2021), *The Living Standards Framework 2021*, <https://www.treasury.govt.nz/sites/default/files/2021-10/tp-living-standards-framework-2021.pdf> | Framework without competencies: Excluded |
| 40 | New Zealand Treasury (2022), *Te Tai Waiora: Wellbeing in Aotearoa New Zealand 2022*, <https://www.treasury.govt.nz/publications/wellbeing-report/te-tai-waiora-2022> | Framework without competencies: Excluded |
| 41 | New Zealand Treasury (2019), “Applying a wellbeing approach to agency planning and performance reporting”, <https://www.treasury.govt.nz/information-and-services/state-sector-leadership/guidance/reporting-financial/applying-wellbeing-approach-agency-planning-and-performance-reporting> | Framework without competencies: Excluded |
| 42 | OECD (2023), “Win-win solutions for well-being and mental health”, *OECD Policy Insights on Well-being, Inclusion and Equal Opportunity*, No. 11, OECD Publishing, Paris, <https://doi.org/10.1787/93b79ccf-en>. | Framework without competencies: Excluded |
| 43 | Barahona, S. et al. (2023), *Economic Policy Making to Pursue Economic Welfare*, OECD Publishing, Paris, <https://doi.org/10.1787/ccc5634c-en>. | Framework without competencies: Excluded |
| 44 | OECD (2023), *Built Environment through a Well-being Lens*, OECD Publishing, Paris, <https://doi.org/10.1787/1b5bebf4-en>. | Framework without competencies: Excluded |
| 45 | Korea’s Quality of Life Indicators [Korea’s Quality of Life Indicators \| OECD](https://www.oecd.org/en/publications/well-being-knowledge-exchange-platform-kep_93d45d63-en/korea-s-quality-of-life-indicators_af2e2bfc-en.html) | Framework without competencies: Excluded |
| 46 | Government of South Korea, Indicators website, <https://www.index.go.kr/unify/intro.do?page=qol> (accessed November 3 2023) | Language |
| 47 | KOSTAT (2023), *Quality of Life Indicators in Korea 2022*, Statistical Research Institute, KOSTAT, <https://kostat.go.kr/board.es?mid=b10105000000&bid=0060> | Framework without competencies: Excluded |
| 48 | Statistics Research Institute (2022), *국민* *삶의* *질 2022 [Quality of Life Indicators in Korea 2022],* https://sri.kostat.go.kr/board.es?mid=a90401000000&bid=11477&list_no=423793&act=view&mainXml=Y; | Framework without competencies: Excluded |
| 49 | Statistics Research Institute (2017), *국민* *삶의* *질* *지표* *개편 [National Report on Quality of Life],* <https://kostat.go.kr/board.es?mid=a90106000000&bid=12316&act=view&list_no=418664&tag=&nPage=1&ref_bid=> | Framework without competencies: Excluded |
| 50 | OECD (2024), “Measuring well-being “beyond GDP” in Asia, South-East Asia and Korea”, *OECD Papers on Well-being and Inequalities*, No. 22, OECD Publishing, Paris, <https://doi.org/10.1787/1487aa23-en>. | Framework without competencies: Excluded |
| 51 | Chile’s Social Well-being Survey [Chile’s Social Well-being Survey \| OECD](https://www.oecd.org/en/publications/well-being-knowledge-exchange-platform-kep_93d45d63-en/chile-s-social-well-being-survey_e3dc9746-en.html) | Framework without competencies: Excluded |
| 52 | “Chile’s Social Well-being Survey: Key Findings and some challenges”, presentation by Matías Cociña at OECD workshop, *Implementing a well-being approach to policy and international partnerships in Latin America*, 28-30 June 2022: <https://www.slideshare.net/StatsCommunications/from-dashboards-to-decisionmaking-adapting-complex-information-on-wellbeing-for-policy-use-matas-cocia> | Framework without competencies: Excluded |
| 53 | Government of Chile, Social Observatory Division (2021), *The Complementary Social Well-being Survey*, <https://www.cepal.org/sites/default/files/presentations/complementary-social-well-being-survey-chile-ministerio-desarrollo-social.pdf> | Framework without competencies: Excluded |
| 54 | Italy’s Equitable and Sustainable Well-being (Bes) Indicators [Italy’s Equitable and Sustainable Well-being (Bes) Indicators \| OECD](https://www.oecd.org/en/publications/well-being-knowledge-exchange-platform-kep_93d45d63-en/italy-s-equitable-and-sustainable-well-being-bes-indicators_2e947d9a-en.html) | Framework without competencies: Excluded |
| 55 | Italian Ministry of Economy and Finance (2023), *Relazione* *sugli* *Indicatori di Benessere* *Equo e Sostenible* *[2023 Equitable and Sustainable Well-being Report to the Parliament]*, <https://www.dt.mef.gov.it/export/sites/sitodt/modules/documenti_it/analisi_progammazione/documenti_programmatici/Relazione-BES-2023.pdf> | Language |
| 56 | Mexico’s Survey of Self-reported Well-being (ENBIARE) [Mexico’s Survey of Self-reported Well-being (ENBIARE) \| OECD](https://www.oecd.org/en/publications/well-being-knowledge-exchange-platform-kep_93d45d63-en/mexico-s-survey-of-self-reported-well-being-enbiare_bf3139e6-en.html) | Framework without competencies: Excluded |
| 57 | Finland’s National Action Plan for the Economy of Wellbeing 2023-25 and related initiatives [Finland’s National Action Plan for the Economy of Wellbeing 2023-25 and related initiatives \| OECD](https://www.oecd.org/en/publications/well-being-knowledge-exchange-platform-kep_93d45d63-en/finland-s-national-action-plan-for-the-economy-of-wellbeing-2023-25-and-related-initiatives_58931a45-en.html) | Framework without competencies: Excluded |
| 58 | Finnish Government (2023), *Action Plan to integrate the economy of wellbeing into decision-making and sustainability assessment*, [https://valtioneuvosto.fi/en/-//1271139/action-plan-to-integrate-the-economy-of-wellbeing-into-decision-making-and-sustainability-assessment](https://valtioneuvosto.fi/en/-/1271139/action-plan-to-integrate-the-economy-of-wellbeing-into-decision-making-and-sustainability-assessment) | Language |
| 59 | Finnish Government (2022), *Strategy of the National Commission on Sustainable Development 2022–2030 : A prosperous and globally responsible Finland that protects the carrying capacity of nature*, <https://julkaisut.valtioneuvosto.fi/handle/10024/164157> | Framework without competencies: Excluded |
| 60 | Finnish Institute for Health and Welfare (2022), *Building a knowledge-based wellbeing economy in Finland,* <https://thl.fi/documents/10531/10313388/THL_Hanke-esite_Building_a_knowledge-based_saavutettava.pdf/62cfe30d-df57-e3c9-93f5-e3ace6c9d1cb?t=1687932151700> | No access |
| 61 | UK Measures of National Well-being [UK Measures of National Well-being \| OECD](https://www.oecd.org/en/publications/well-being-knowledge-exchange-platform-kep_93d45d63-en/uk-measures-of-national-well-being_f2eac2e0-en.html) | Framework without competencies: Excluded |
| 62 | Spain’s Quality of Life Indicators [Spain’s Quality of Life Indicators \| OECD](https://www.oecd.org/en/publications/well-being-knowledge-exchange-platform-kep_93d45d63-en/spain-s-quality-of-life-indicators_f7c57897-en.html) | Framework without competencies: Excluded |
| 63 | Wales’s Children and Young People’s Plan [Wales’s Children and Young People’s Plan \| OECD](https://www.oecd.org/en/publications/well-being-knowledge-exchange-platform-kep_93d45d63-en/wales-s-children-and-young-people-s-plan_b0ee041b-en.html) | Framework without competencies: Excluded |
| 64 | Children in Wales (2022), *Welsh Government’s Draft Children and Young People’s Plan Consultation: Young Wales Findings Report on behalf of Welsh Government*, <https://www.childreninwales.org.uk/application/files/5916/4606/4043/CYP_Report_English.pdf> | Framework without competencies: Excluded |
| 65 | Children in Wales (2022), *Welsh Government’s Draft Children and Young People’s Plan Consultation: Young Wales Findings Report on behalf of Welsh Government*, <https://www.childreninwales.org.uk/application/files/5916/4606/4043/CYP_Report_English.pdf> | Framework without competencies: Excluded |
| 66 | OECD (2023), “Integrated policy making for child well-being: Common approaches and challenges ahead”, *OECD Papers on Well-being and Inequalities*, No. 16, OECD Publishing, Paris, <https://doi.org/10.1787/1a5202af-en>. | Framework without competencies: Excluded |
| 67 | Kudrna, L. et al. (2024), “Measuring affective components of subjective well-being: Updated evidence to inform national data collections”, *OECD Papers on Well-being and Inequalities*, No. 31, OECD Publishing, Paris, <https://doi.org/10.1787/6c72da70-en>. | Framework without competencies: Excluded |
| 68 | How’s Austria? [How’s Austria? \| OECD](https://www.oecd.org/en/publications/well-being-knowledge-exchange-platform-kep_93d45d63-en/how-s-austria_dc85db91-en.html) | Framework without competencies: Excluded |
| 69 | Scotland’s National Performance Framework and efforts to build a Wellbeing Economy [Scotland’s National Performance Framework and efforts to build a Wellbeing Economy \| OECD](https://www.oecd.org/en/publications/well-being-knowledge-exchange-platform-kep_93d45d63-en/scotland-s-national-performance-framework-and-efforts-to-build-a-wellbeing-economy_b58ad8e5-en.html) | Framework without competencies: Excluded |
| 70 | Scottish Government, Wellbeing Economy Governments (WEGo) website, <https://www.gov.scot/groups/wellbeing-economy-governments-wego/> (accessed October 30 2023) | Framework without competencies: Excluded |
| 71 | Portugal’s Well-being Index [Portugal’s Well-being Index \| OECD](https://www.oecd.org/en/publications/well-being-knowledge-exchange-platform-kep_93d45d63-en/portugal-s-well-being-index_ea1c6741-en.html) | Framework without competencies: Excluded |
| 72 | Statistics Portugal, Well-being Index Framework and Objectives website, <https://www.ine.pt/xportal/xmain?xpid=INE&xpgid=ine_contextobjetivos&xlang=en> (accessed October 27 2023) | Framework without competencies: Excluded |
| 73 | The Luxembourg Index of Well-being (LIW) [The Luxembourg Index of Well-being (LIW) \| OECD](https://www.oecd.org/en/publications/well-being-knowledge-exchange-platform-kep_93d45d63-en/the-luxembourg-index-of-well-being-liw_5c51a395-en.html) | Framework without competencies: Excluded |
| 74 | The Netherlands’ Monitor of Well-being and the Sustainable Development Goals [The Netherlands’ Monitor of Well-being and the Sustainable Development Goals \| OECD](https://www.oecd.org/en/publications/well-being-knowledge-exchange-platform-kep_93d45d63-en/the-netherlands-monitor-of-well-being-and-the-sustainable-development-goals_c0f07945-en.html) | Framework without competencies: Excluded |
| 75 | Agriculture in a Wellbeing Economy By Milo Costanza-van den Belt <https://weall.org/wp-content/uploads/WeAll-BRIEFINGS-PAPERS-agriculture.pdf> | Framework without competencies: Excluded |
| 76 | Failure Demand Counting the true costs of an unjust and unsustainable economic system <https://weall.org/wp-content/uploads/FailureDemand_FinalReport_September2021-1.pdf> | Framework without competencies: Excluded |
| 77 | Zero Waste in a Wellbeing Economy Lead Author: Malin Leth <https://weall.org/wp-content/uploads/WEAll-BRIEFING-PAPER-Zero-Waste.pdf> | Framework without competencies: Excluded |
| 78 | The Commons in a Wellbeing Economy Written by: Munro Fraser and Thomas Mande [WEAllBriefing_Commons_IN_15November2021-1-1.pdf](https://weall.org/wp-content/uploads/WEAllBriefing_Commons_IN_15November2021-1-1.pdf) | Framework without competencies: Excluded |
| 79 | Water in a Wellbeing Economy Written by: Rohit Rao and Hugh Coppell [WEAllBriefing_Water_IN_3August2021-1.pdf](https://weall.org/wp-content/uploads/WEAllBriefing_Water_IN_3August2021-1.pdf) | Framework without competencies: Excluded |
| 80 | Wellbeing Business 8 ways that businesses are challenging the corporate mindset to ensure social and ecological wellbeing for all [WEAll-BRIEFINGS-Wellbeing-Business_3August-1.pdf](https://weall.org/wp-content/uploads/WEAll-BRIEFINGS-Wellbeing-Business_3August-1.pdf) | Framework without competencies: Excluded |
| 81 | Measuring the Wellbeing Economy: How to Go Beyond-GDP [WEAll-BRIEFINGS-Measuring-the-Wellbeing-Economy_3Aug-1.pdf](https://weall.org/wp-content/uploads/WEAll-BRIEFINGS-Measuring-the-Wellbeing-Economy_3Aug-1.pdf) | Framework without competencies: Excluded |
| 82 | Five Pathways Toward Health-Environment Policy in a Wellbeing Economy [WEAll-POLICY-PAPER-Health-and-Environment_3August-1.pdf](https://weall.org/wp-content/uploads/WEAll-POLICY-PAPER-Health-and-Environment_3August-1.pdf) | Framework without competencies: Excluded |
| 83 | Wellbeing Economy Policy Design Guide How to design economic policies that put the wellbeing of people and the planet first [Wellbeing-Economy-Policy-Design-Guide_Mar12_FINAL-1.pdf](https://weall.org/wp-content/uploads/Wellbeing-Economy-Policy-Design-Guide_Mar12_FINAL-1.pdf) | Framework without competencies: Excluded |
| 84 | A Guide to the Alternatives to Business as Usual The Business of Wellbeing [The-Business-of-Wellbeing-guide-Web.pdf](https://weall.org/wp-content/uploads/2020/01/The-Business-of-Wellbeing-guide-Web.pdf) | Framework without competencies: Excluded |
| 85 | WEAll Briefing Papers: Little Summaries of Big Issues Understanding Wellbeing [WEAll-Briefing-Understanding-Wellbeing_July2020-1.pdf](https://weall.org/wp-content/uploads/WEAll-Briefing-Understanding-Wellbeing_July2020-1.pdf) | Framework without competencies: Excluded |
| 86 | Wellbeing Economics for the COVID-19 recovery Ten principles to build back better [WEAll BP WE for Covid-19_v007](https://weall.org/wp-content/uploads/Wellbeing_Economics_for_the_COVID-19_recovery_10Principles.pdf) | Framework without competencies: Excluded |
| 87 | WEAll Ideas: Little Summaries of Big Issues ‘7 Ideas for the G7’ By Amanda Janoo [Microsoft Word - Seven Ideas for the G7 2019 as at Aug 22 2019.docx](https://wellbeingeconomy.org/wp-content/uploads/2019/08/Seven-Ideas-for-the-G7-2019.pdf) | Framework without competencies: Excluded |
| 88 | “Decerf, Benoit. 2024. Multidimensional Well-Being Measurement Practices: A Review Focused on Improving Global Multidimensional Poverty Indicators. Policy Research Working Paper; 10800. © Washington, DC: World Bank. http://hdl.handle.net/10986/41703 License: [CC BY 3.0 IGO](https://creativecommons.org/licenses/by/3.0/igo/).” | Framework without competencies: Excluded |
| 89 | “Armijo, Estela; Asnake, Tina. 2025. The Effects of Early Childhood Development (ECD) Programs on the Socio-emotional Well-being of Children and Caregivers in Refugee and Forced Displacement Settings. Education Working Paper; No. 6, November 2024. © Washington, DC: World Bank. http://hdl.handle.net/10986/42756 License: [CC BY-NC 3.0 IGO](https://creativecommons.org/licenses/by-nc/3.0/igo).” | Included |
| 90 | “Bergstrom, Katy; Ozler, Berk. 2021. Improving the Well-Being of Adolescent Girls in Developing Countries. Policy Research Working Paper;No. 9827. © World Bank, Washington, DC. http://hdl.handle.net/10986/36478 License: [CC BY 3.0 IGO](http://creativecommons.org/licenses/by/3.0/igo).” | Included |
| 91 | “Witte, Caroline T.; Burger, Martijn J.; Ianchovichina, Elena. 2019. Subjective Well-Being and Peaceful Uprisings. Policy Research Working Paper;No. 8705. © World Bank, Washington, DC. http://hdl.handle.net/10986/31173 License: [CC BY 3.0 IGO](http://creativecommons.org/licenses/by/3.0/igo).” | Framework without competencies: Excluded |
| 92 | “Saadat, Seemeen; Cortez, Rafael A.. 2017. Health and Well-Being of Young People: A Multi-Sectoral Approach to Operational Planning. Health, Nutrition and Population Knowledge Brief;. © World Bank, Washington, DC. http://hdl.handle.net/10986/27134 License: [CC BY 3.0 IGO](http://creativecommons.org/licenses/by/3.0/igo).” | Framework without competencies: Excluded |
| 93 | “Chase, Claire; Damania, Richard. 2017. Water, Well-Being, and the Prosperity of Future Generations. Water Global Practice discussion paper;. © World Bank, Washington, DC. http://hdl.handle.net/10986/26203 License: [CC BY 3.0 IGO](http://creativecommons.org/licenses/by/3.0/igo).” | Framework without competencies: Excluded |
| 94 | “Hamilton, Kirk; Helliwell, John; Woolcock, Michael. 2016. Social Capital, Trust, and Well-being in the Evaluation of Wealth. Policy Research Working Paper;No. 7707. © World Bank, Washington, DC. http://hdl.handle.net/10986/24624 License: [CC BY 3.0 IGO](http://creativecommons.org/licenses/by/3.0/igo/).” | Framework without competencies: Excluded |
| 95 | “Murgai, Rinku; Narayan, Ambar. 2016. Looking Back on Two Decades of Poverty and Well-Being in India. Policy Research Working Paper;No. 7626. © World Bank, Washington, DC. http://hdl.handle.net/10986/24168 License: [CC BY 3.0 IGO](http://creativecommons.org/licenses/by/3.0/igo/).” | Framework without competencies: Excluded |
| 96 | “Auturupane, Harsha; Gunatilake, Ramani; Shojo, Mari; Ebenezer, Roshini. 2013. Educational, Economic Welfare and Subjective Well-Being in Afghanistan. South Asia Human Development Sector Discussion Paper;No. 63. © World Bank, Washington, DC. http://hdl.handle.net/10986/16284 License: [CC BY 3.0 IGO](http://creativecommons.org/licenses/by/3.0/igo).” | Framework without competencies: Excluded |
| 97 | Wietzke, Frank-Borge; McLeod, Catriona. 2012. Jobs, Well-Being, and Social Cohesion : Evidence from Value and Perception Surveys. Background Paper for the World Development Report 2013;. © World Bank, Washington, DC. http://hdl.handle.net/10986/12138 License: [CC BY 3.0 IGO](http://creativecommons.org/licenses/by/3.0/igo/).” | Framework without competencies: Excluded |
| 98 | “World Bank. 2025. Regulatory Sandboxes for Digital Health. © Washington, DC: World Bank. http://hdl.handle.net/10986/42711 License: [CC BY-NC 3.0 IGO](https://creativecommons.org/licenses/by-nc/3.0/igo).” | Framework without competencies: Excluded |

**Table 3.** Handsearching

| # |  |
| --- | --- |
| 1 | “Armijo, Estela; Asnake, Tina. 2025. The Effects of Early Childhood Development (ECD) Programs on the Socio-emotional Well-being of Children and Caregivers in Refugee and Forced Displacement Settings. Education Working Paper; No. 6, November 2024. © Washington, DC: World Bank. http://hdl.handle.net/10986/42756 License: [CC BY-NC 3.0 IGO](https://creativecommons.org/licenses/by-nc/3.0/igo).” |
| Downwards | 38 references,0 selected  Note: some references were not updated. Some references were not public domain. Other references were already covered in the review |
| Upwards | 0 references, 0 selected |
| **Overall:** | 38 |
| 2 | Lee, J. and Ž. Žarnic (2024), “The impact of digital technologies on well-being: Main insights from the literature”, *OECD Papers on Well-being and Inequalities*, No. 29, OECD Publishing, Paris, <https://doi.org/10.1787/cb173652-en>. |
| Downwards | 214 references, 1 included   I. OECD (2023), *OECD Skills Outlook 2023: Skills for a Resilient Green and Digital Transition*, OECD Publishing, Paris, <https://doi.org/10.1787/27452f29-en>. |
| Upwards | 0 references, 0 selected |
| **Overall:** | 252 |
| 3 | Sangha, K. K., Dinku, Y., Costanza, R., & Poelina, A. (2024). A comprehensive analysis of well-being frameworks applied in Australia and their suitability for Indigenous peoples. *International journal of qualitative studies on health and well-being*, *19*(1), 2321646. <https://doi.org/10.1080/17482631.2024.2321646> |
| Downwards | 53 references, 1 included   II. Commonwealth of Australia . (2017). National Strategic Framework for Aboriginal and Torres Strait Islander Peoples’ Mental Health and Social and Emotional Wellbeing (2017-2023). Department of the Prime Minister and Cabinet, the Australian Government. [National Strategic Framework for Aboriginal and Torres Strait Islander Peoples’ Mental Health and Social and Emotional Wellbeing 2017-2023](https://www.niaa.gov.au/sites/default/files/documents/publications/mhsewb-framework_0.pdf) |
| Upwards | 3 refences, 0 selected |
| **Overall:** | 308 |
| 4 | Developing Skills for Digital Government: A review of good practices across OECD governments 2024 [pdf](https://one.oecd.org/document/DELSA/ELSA/WD/SEM(2024)1/en/pdf) |
| Downwards | 57 references, 1 included   III. Vuorikari, R., Kluzer, S. and Punie, Y. (2022). DigComp 2.2: The Digital Competence Framework for Citizens - With new examples of knowledge, skills and attitudes, EUR 31006 EN, Publications Office of the European Union, Luxembourg, ISBN 978-92-76-48882-8, doi:10.2760/115376, JRC128415. |
| Upwards | 5 references, 0 included  Note some references were written in another language and others were not available. |
| **Overall:** | 370 |
| 5 | Working Party on Artificial Intelligence Governance Assessing potential future artificial intelligence risks, benefits and policy imperatives Perspectives from the OECD Expert Group on AI Futures 2024 [pdf](https://one.oecd.org/document/DSTI/CDEP/AIGO(2023)13/FINAL/en/pdf) |
| Downwards | 321 references, 0 selected |
| Upwards | 0 references |
| **Overall:** | 691 |
| 6 | Del Rio, M., Lasley, P., Tallon, L., Kauth, J.-M., Bare, G., McCurdy, L.E., & Etzel, R.A. (2024). Performance indicators corresponding to the critical competencies in children's environmental health. Journal of Environmental Health, 86 (10),24-29. |
| Downwards | 22 references, 1 selected   IV. Del Rio, M., Lasley, P., Tallon, L., Kauth, J.-M., Bare, G., McCurdy, L.E., & Etzel, R.A. (2023). Critical competencies in children’s environmental health. Journal of Environmental Health, 85(6), 26–29. |
| Upwards | 0 references |
| **Overall:** | 713 |
| 7 | Doshmangir, L., Alipouri Sakha, M., Mostafavi, H., Kabiri, N., Ghaffarifar, S., & Takian, A. (2024). Essential core competencies for health policy graduates: a multi-method consensus type study. *Health research policy and systems*, *22*(1), 136. <https://doi.org/10.1186/s12961-024-01221-8> |
| Downwards | 51 references, 0 references selected |
| Upwards | 0 references |
| **Overall:** | 764 |
| 8 | Country deep dive on the well-being economy: Iceland. Copenhagen: WHO Regional Office for Europe; 2023. Licence: CC BY-NC-SA 3.0 IGO [WHO-EURO-2023-7415-47181-69111-eng.pdf](https://iris.who.int/bitstream/handle/10665/369105/WHO-EURO-2023-7415-47181-69111-eng.pdf?sequence=1) |
| Downwards | 96 references, 0 selected |
| Upwards | 2 references, 0 selected |
| **Overall:** | 862 |
| 9 | Nykiforuk, C. I. J., Belon, A. P., de Leeuw, E., Harris, P., Allen-Scott, L., Atkey, K., Glenn, N. M., Hyshka, E., Jaques, K., Kongats, K., Montesanti, S., Nieuwendyk, L. M., Pabayo, R., Springett, J., & Yashadhana, A. (2023). An action-oriented public health framework to reduce financial strain and promote financial wellbeing in high-income countries. International Journal for Equity in Health, 22(1). https://doi.org/10.1186/s12939-023-01877-8 |
| Downwards | 51 references, 0 selected |
| Upwards | 7 references, 0 selected |
| **Overall:** | 920 |
| 10 | Porcelli, A., D’Onise, K., & Pontifex, K. (2023). Public health partner authorities-How a health in all policies approach could support the development of a wellbeing economy. Health Promotion Journal of Australia : Official Journal of Australian Association of Health Promotion Professionals, 34(3), 671–674. https://doi.org/10.1002/hpja.738 |
| Downwards | 12 references, 0 selected |
| Upwards | 2 references, 0 selected |
| **Overall:** | 934 |
| 11 | Capetola, T., Noy, S., & Patrick, R. (2022). Planetary health pedagogy: Preparing health promoters for 21st‐century environmental challenges. Health Promotion Journal of Australia, 33(Suppl 1), 17–21. https://doi.org/10.1002/hpja.641 |
| Downwards | 27 references, 0 selected |
| Upwards | 12 references, 1 selected   V. Simon, J., Parisi, S., Wabnitz, K., Simmenroth, A., & Schwienhorst-Stich, E.-M. (2023). Ten characteristics of high-quality planetary health education—Results from a qualitative study with educators, students as educators and study deans at medical schools in Germany. Frontiers in Public Health, 11. https://doi.org/10.3389/fpubh.2023.1143751 |
| **Overall:** | 973 |
| 12 | Wellbeing Economy Toolkit Supporting place-based economic strategy and policy development. 2022  [Executive Summary - Wellbeing economy toolkit: supporting place based economic strategy and policy development - gov.scot](https://www.gov.scot/publications/wellbeing-economy-toolkit-supporting-place-based-economic-strategy-policy-development/pages/1/) |
| Downwards | 0 references |
| Upwards | 0 references |
| **Overall:** | 973 |
| 13 | “Bergstrom, Katy; Ozler, Berk. 2021. Improving the Well-Being of Adolescent Girls in Developing Countries. Policy Research Working Paper;No. 9827. © World Bank, Washington, DC. http://hdl.handle.net/10986/36478 License: [CC BY 3.0 IGO](http://creativecommons.org/licenses/by/3.0/igo).” [World Bank Document](https://openknowledge.worldbank.org/server/api/core/bitstreams/d6340e66-f894-5c64-9026-b09ef6a9434a/content) |
| Downwards | 0 references |
| Upwards | 38 references, 0 selected |
| **Overall:** | 1011 |
| 14 | Jordan, M. A. (2021). The Role of the Health Coach in a Global Pandemic. Global Advances in Health and Medicine, 10. https://doi.org/10.1177/21649561211039456 |
| Downwards | 74 references, 0 selected |
| Upwards | 9 references, 0 selected |
| **Overall:** | 1094 |
| 15 | World Health Organization. Regional Office for Europe. (‎2020)‎. Health and well-being in the voluntary national reviews of the 2030 Agenda for Sustainable Development in the WHO European Region 2016–2020. World Health Organization. Regional Office for Europe. <https://iris.who.int/handle/10665/340007>. License: CC BY-NC-SA 3.0 IGO |
| Downwards | 87 references, 0 selected |
| Upwards | 3 references, 0 selected |
| **Overall:** | 1184 |
| 16 | World Health Organization. Regional Office for Europe. (‎2013)‎. Health 2020: a European policy framework supporting action across government and society for health and well-being (‎short version)‎. World Health Organization. Regional Office for Europe. <https://iris.who.int/handle/10665/131300> |
| Downwards | 0 references |
| Upwards | 12 references, 0 selected |
| **Overall:** | 1196 |
| 17 | Powell, N., Dalton, H., Lawrence-Bourne, J., & Perkins, D. (2024). Co-creating community wellbeing initiatives: what is the evidence and how do they work? *International Journal of Mental Health Systems*, *18*(1). <https://doi.org/10.1186/s13033-024-00645-7> |
| Downwards | 120 references, 0 selected |
| Upwards | 3 references, 0 selected |
| **Overall:** | 1319 |
| 18 | Sebong, P. H., Pardosi, J., Goldman, R. E., Suryo, A. P., Susianto, I. A., & Meliala, A. (2024). Identifying Physician Public Health Competencies to Address Healthcare Needs in Underserved, Border, and Outer Island Areas of Indonesia: A Rapid Assessment. Teaching and Learning in Medicine, 1–12. <https://doi.org/10.1080/10401334.2024.2353573> |
| Downwards | 63 references, 0 selected |
| Upwards | 1 reference, no access to that reference. |
| **Overall:** | 1383 |
| 19 | Hayman, D. T. S., Barraclough, R. K., Muglia, L. J., McGovern, V., Afolabi, M. O., N’Jai, A. U., Ambe, J. R., Atim, C., McClelland, A., Paterson, B., Ijaz, K., Lasley, J., Ahsan, Q., Garfield, R., Chittenden, K., Phelan, A. L., & Lopez Rivera, A. (2023). Addressing the challenges of implementing evidence-based prioritisation in global health. BMJ Global Health, 8(6). <https://doi.org/10.1136/bmjgh-2023-012450> |
| Downwards | 51 references, 0 selected |
| Upwards | 8 references, 0 selected |
| **Overall:** | 1442 |
| 20 | Betley, E. C., Sigouin, A., Pascua, P., Cheng, S. H., MacDonald, K. I., Arengo, F., Aumeeruddy-Thomas, Y., Caillon, S., Isaac, M. E., Jupiter, S. D., Mawyer, A., Mejia, M., Moore, A. C., Renard, D., Sébastien, L., Gazit, N., & Sterling, E. J. (2023). Assessing human well-being constructs with environmental and equity aspects: A review of the landscape. People and Nature, 5(6), 1756–1773. <https://doi.org/10.1002/pan3.10293> |
| Downwards | 131 references, 0 selected |
| Upwards | 41 references, 0 selected |
| 21 | Scotland’s National Performance Framework Our Purpose, Values and National Outcomes [National Performance Framework \| National Performance Framework](https://nationalperformance.gov.scot/) [NPF_A4_Booklet.pdf](https://nationalperformance.gov.scot/sites/default/files/documents/NPF_A4_Booklet.pdf) |
| Downwards | No references |
| Upwards | No references |
| 22 | Scottish Government. n.d. Local Wellbeing Economy Monitor: Guidance. <https://view.officeapps.live.com/op/view.aspx?src=https%3A%2F%2Fwww.gov.scot%2Fbinaries%2Fcontent%2Fdocuments%2Fgovscot%2Fpublications%2Fadvice-and-guidance%2F2022%2F11%2Fwellbeing-economy-toolkit-supporting-place-based-economic-strategy-policy-development%2Fdocuments%2Fwellbeing-economy-monitor-guidance-updated-february-2024%2Fwellbeing-economy-monitor-guidance-updated-february-2024%2Fgovscot%253Adocument%2Fwellbeing-economy-monitor-guidance-updated-february-2024.docx&wdOrigin=BROWSELINK> |
| Downwards | 17 references, 0 selected |
| Upwards | No references |
| **Overall:** | 1631 |

**Table 4.** Consultation: the main author.

| # | Reference | Include or exclude: Reason |
| --- | --- | --- |
| 1 | Sangha KK, Gordon IJ and Costanza R (2023) Editorial: Ecosystem services, policy, and human well-being. *Front. Ecol. Evol.* 11:1174160. doi: 10.3389/fevo.2023.1174160 | Study design without methods: Excluded |
| 2 | Sangha, K. K., Dinku, Y., Costanza, R., & Poelina, A. (2024). A comprehensive analysis of well-being frameworks applied in Australia and their suitability for Indigenous peoples. *International journal of qualitative studies on health and well-being*, *19*(1), 2321646. <https://doi.org/10.1080/17482631.2024.2321646> | Duplicated (database: Already): Excluded |
| 3 | Del Rio, M., Lasley, P., Tallon, L., Kauth, J.-M., Bare, G., McCurdy, L.E., & Etzel, R.A. (2023). Critical competencies in children’s environmental health. Journal of Environmental Health, 85(6), 26–29. | Duplicated (Handsearching: Already): Excluded |
